# Heat but Not Cold Tolerance Is Phylogenetically Constrained in Greenlandic Terrestrial Arthropods Under Future Global Warming

**DOI:** 10.1111/gcb.70687

**Published:** 2026-01-08

**Authors:** Jonas Bruhn Wesseltoft, Nadieh de Jonge, Michael Møller Hansen, Toke Thomas Høye, Michael Ørsted, Torsten Nygaard Kristensen

**Affiliations:** ^1^ Department of Chemistry and Bioscience Aalborg University Aalborg Denmark; ^2^ Department of Biology Aarhus University Aarhus Denmark; ^3^ Department of Ecoscience and Arctic Research Centre Aarhus University Aarhus Denmark

**Keywords:** Arachnida, Arctic, Bioblitz, collembola, CTmax, CTmin, Insecta, molecular species identification, thermal limits

## Abstract

The Arctic is currently warming at up to four times the global average. Likewise, climate variability within and across seasons is predicted to markedly increase in the future. This may challenge organisms in these pristine environments, making it critically important to understand their thermal biology and evolutionary potential. For Arctic ectotherms in particular, thermal tolerance limits and responses to climate change are mostly unknown. Knowledge on this is urgently needed to enable prediction of climate change impacts on future distributions of biodiversity in these rapidly changing environments. Here, we provide data on upper and lower thermal limits of 93 Greenlandic species of insects, arachnids, and collembolans caught and tested in the field and identified using barcode sequencing. This represents ~8% of described terrestrial Greenlandic arthropod species. We found pronounced differences in heat and cold tolerance among species and a strong phylogenetic signal for both heat tolerance and thermal scope (difference between upper and lower thermal limits). We argue that this indicates a limited capacity for coping with increasing and more variable future temperatures through evolutionary adaptation. Further, we modelled future increases in microhabitat temperatures in our sampling area. We found that > 25% of the investigated species may periodically experience stressful high temperatures in the future. These results suggest that climate change will likely result in substantial changes in distributions and abundances of terrestrial arthropods in Southern Greenland.

## Graphical abstract

In this study we describe the temperature limits for almost 8% of the Greenlandic arthropod fauna (insects and spiders). By combining this with a DNA analysis and a projection of future climates inhabited by these species we reveal that upwards of 25% of the investigated species risk exposure to stressful temperatures by the end of the century and that they are potentially limited in their ability to adapt evolutionarily to higher temperatures. This suggests that the climatic changes predicted over the next century are likely to have pronounced effects on pristine Greenlandic ecosystems.

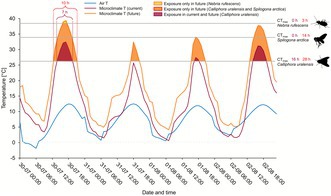

## 1 Introduction

As the Earth continues to warm, knowledge on the thermal tolerance limits of organisms is critical for predicting how ecosystems will respond and change (Jørgensen et al. 2022; Antão et al. 2022; Bellard et al. 2012; Muluneh 2021). Such changes in ecosystems will be partly driven by increasing mean temperatures and more frequent extreme weather events as this can cause many species to experience stressful temperatures exceeding their optimal range (Jørgensen et al. 2022; Sunday et al. 2014; Johansson et al. 2020). This can result in heat or cold stress, which have marked negative impacts on multiple life‐history traits and can ultimately be lethal (Hoffmann et al. 2023; van Heerwaarden and Sgrò 2021; Parratt et al. 2021). When populations and species experience climatic stress, they can either migrate, adapt through evolution (Sunday et al. 2014; Bennett et al. 2021), or via plasticity in physiological, behavioural and/or morphological traits (Sunday et al. 2014; Kristensen et al. 2020; Ørskov et al. 2019). However, recent findings have suggested that while animals, and ectotherms in particular, can adapt to colder environments through evolution and plasticity, their capacity to increase heat tolerance through these mechanisms is limited (Bennett et al. 2021; Kellermann et al. 2012; Hoffmann et al. 2013; Weaving et al. 2022). The warming rate in southern Greenland, where this study was done, is between two and three times higher than the global mean (Rantanen et al. 2022) making it of utmost importance to study the effects of climate change on the thermal biology of arthropods here (Høye 2020). Further, a low species diversity in this region (as in other polar regions) makes it a relatively simple system to investigate (Böcher et al. 2015). Globally, knowledge of thermal tolerance across species and genera of arthropods is limited (Herrando‐Pérez et al. 2023; Bennett et al. 2018), and there has recently been a call for more focus on thermal tolerance limits and adaptations to climate stress of arthropods, especially in polar and mountainous regions (Høye 2020; Bahrndorff et al. 2021, 2025; Matheson and McGaughran 2022). Historically, work on thermal biology of arthropods from high latitudes and altitudes has focused on their ability to cope with low temperatures (Høye 2020; Matheson and McGaughran 2022; Teets et al. 2012; Sømme 1981) and only recently have studies on responses to high temperature stress been performed (Bahrndorff et al. 2021; Noer et al. 2023; Daly et al. 2024; Beet et al. 2022; Morley et al. 2022). Efforts to reconcile the fragmented knowledge of thermal tolerances across species and latitudes into centralised databases clearly illustrate the lack of data, as less than 0.0003% of described terrestrial arthropod species have estimates of thermal stress tolerance levels in the database GLOBTHERM (Bennett et al. 2018). Thus, more data on more species are urgently needed to expand our understanding of consequences of climate changes globally.

Heat or cold tolerance of ectotherms is often reported as the critical thermal limit of a given species, that is, the highest (CT_max_) or lowest (CT_min_) temperature at which the organism displays ecologically relevant functions like locomotion (Overgaard et al. 2012). Information on thermal tolerance measures like these is crucial for predicting future distributions as they provide important information on the environmental conditions in which the given species can thrive. We propose that this is especially relevant to investigate in Southern Greenland where temperatures change rapidly with climate change and where microhabitat temperatures above 30°C and below 0°C are often registered during summer days and nights, respectively (Noer et al. 2022), with considerable variation due to varying vegetation cover and height, terrain, wind, solar radiation, soil moisture, etc. (Rebaudo et al. 2016; Kearney et al. 2014). During winter, temperatures as low as −30°C are regularly registered (Laird et al. 2024), although the temperature experienced by small organisms in their microhabitat may be buffered by snow cover, and in sheltered areas like crevices and beneath boulders (Coulson et al. 2023). Given the high temperature span that arthropods in this region are potentially exposed to within and across seasons, we propose that also the thermal scope is a relevant trait to investigate. We define thermal scope as the difference between mean CT_max_ and mean CT_min_ observed for a given species. This approach is similar to the one used in the literature (Overgaard et al. 2012; Pörtner et al. 2006; Tewksbury et al. 2008; Ruthsatz et al. 2022; Ørsted et al. 2022), where ‘thermal window’, ‘temperatures an animal can tolerate’ or ‘thermal tolerance breadth’ are often used to describe the same phenomenon. Information on CT_max_, CT_min_, and thermal scope for Greenlandic arthropods will elaborate our understanding of the vulnerability of species in a region experiencing extremely fast and pronounced climate changes.

In this study performed in Southern Greenland, we adopt a catch‐test‐and‐sequence methodology, enabling simultaneous determination of thermal limits using ramping assays (Overgaard et al. 2012; Cowles and Bogert 1944; Terblanche et al. 2007) and species identification utilizing cytochrome c oxidase I (COI) barcoding combined with morphological identification. Ramping assays, where animals are exposed to temperatures that are gradually increased or decreased are commonly used to obtain estimates of upper (CT_max_) or lower (CT_min_) temperatures that for example, insects can tolerate before they die or go into coma (Overgaard et al. 2012; Ruthsatz et al. 2022). Here we present heat (CT_max_) and/or cold (CT_min_) tolerance data as well as data on thermal scope (CT_max_—CT_min_) on 93 species of terrestrial Greenlandic insects, spiders and collembolans covering ~8% of the described terrestrial Greenlandic arthropod species. Our results reveal marked inter‐specific differences in thermal tolerance measures and a clear phylogenetic signal for both upper thermal limits and thermal scope. We discuss that this could point to evolutionary constraints in these traits and consider possible implications of the findings. We further show that with predicted local future microhabitat temperatures ca. 25% of the investigated species will likely suffer from future high temperature stress during summer peak temperatures. These results provide evidence that rapid climate changes will likely have strong impact on the future distribution and abundance of arthropods in Southern Greenland.

## 2 Materials and Methods

### 2.1 Sample Collection

Terrestrial arthropods (insects, arachnids and collembola) were collected in the field during summer 2023 in Narsarsuaq, Southern Greenland (61.160° N, 45.424° W). This region is characterized by cool temperatures, long winters and short summers (average −7°C in January and +11°C in July, Figure S1A). The area has recently experienced rapidly increasing temperatures with mean annual temperature anomalies exceeding +1°C on average (range −1.7 to +4.4°C) since 2000 compared to the 1979–2023 baseline (Figure S1B). Importantly, summers in the area are very thermally variable with maximum temperatures at ground level exceeding 30°C (Noer et al. 2022). During the sampling period in August 2023 ambient temperature was measured at 6 cm below ground and 2 and 15 cm above ground using TMS‐4 loggers (TOMST; Maclean et al. 2021) at 5 min resolution in two locations in the sampling area in Narsarsuaq (Figure S2 and Appendix S1). The study site is a heath‐like, grass‐ and shrub‐covered area, where the TMS‐4 loggers were placed among grasses and close to taller shrubs to reflect the overall diversity of vegetation height and structure. Each logger was covered with a white plastic cap to reduce the effect of radiative fluxes. Arthropods were collected indiscriminately from grasses and bushes using a sweep net or a pooter (a flexible tube used to catch arthropods directly from leaves or the ground). All individuals used in the experiment were caught in a 200 × 200 m area right next to our laboratory facilities. Animals were collected in the summer season from the 12th of August until the 17th of August 2023. All animals were collected during the day between 09:00 and 19:00, and all days throughout the study period showed similar temperature profiles (Figure S2). For each collection round we spent 30–60 min on sampling in the designated area. The sampling efforts were stochastic, in the way that we did not sample the area in a systematic way but attempted to cover the whole area every time we sampled arthropods.

After collecting animals in the field, they were added to a BugDorm (dimensions: W32.5 × D32.5 × H32.5). From there we collected individuals with a pooter and transferred them to 5 mL glass vials with plastic screw‐lids within 30 min after finalising the collection. Individuals transferred to the glass vials were chosen partly based on novel morphotypes to test as many species as possible (thus we did not choose individuals randomly among the sampled animals). The vials with arthropods were kept outside of the laboratory in the shade for a maximum of 1 h before being tested for either heat or cold tolerance. Animals were collected on five separate days constituting six batches. In each batch, between 80 and 138 individuals were tested. In total, 701 individuals were assessed for either heat or cold stress tolerance.

### 2.2 Critical Thermal Limits

To test for critical thermal limits, vials with arthropods were placed randomly in a rack before being submerged in a circulating water bath for Critical Thermal maximum (CT_max_) or Critical Thermal minimum (CT_min_) assessments (three batches of arthropods were tested for CT_max_ and three were tested for CT_min_; Cowles and Bogert 1944). Liquid in the water baths was kept at 20°C until vials with animals were submerged, whereafter the temperature was increased or lowered by 0.1°C per minute to assess CT_max_ or CT_min_, respectively. We chose to use this specific rate as this is comparable to the rate employed in many other ectotherm studies using ramping assays to assess heat or cold tolerance (Overgaard et al. 2012, 2011; Jørgensen et al. 2021), and because it matched observed rates of temperature change in the field during sampling in our study, suggesting that this is an ecologically relevant rate of temperature change (see discussion and Appendix S1). Vials were monitored continuously and the temperature of the onset of coma, that is, at which the last movement was observed in response to agitation (facilitated by softly knocking on the vials with a metal rod), was recorded as the thermal tolerance limit to heat or cold stress for that individual (Overgaard et al. 2012). Immediately following the critical thermal limit test, each individual was transferred to a 1.5 mL Eppendorf tube before being stored at −20°C until imaging and DNA extraction (to be used for species identification based on DNA barcoding).

### 2.3 Imaging

Imaging was performed using an Olympus SZX10 stereo microscope and Olympus DP74 colour camera (Olympus Corporation). Animals were thawed in small batches of between 1 and 10 individuals before being placed on a 1% agarose plate facilitating proper positioning of the animals. Each animal was pictured from multiple angles, including ventral, dorsal, and profile in addition to identifying markers, that is, wing webbings or cornicles. Additionally, each animal was measured from the tip of the head to the end of the abdomen, and a micro‐ruler was imaged at the same magnification for scale. Following imaging, each animal was returned to the Eppendorf tube and kept at 5°C until DNA extraction was performed on the following day. Representative images of each observed species can be found in Appendix S2 while images of all individuals can be accessed at Dryad depository (https://doi.org/10.5061/dryad.1g1jwsv6w).

### 2.4 DNA Extraction

All specimens were washed twice in 200 μL 2.5% bleach solution (Thermo Fisher Scientific) and twice in 800 μL UV treated ELGA water to minimize cross contamination from sample collection and imaging. Total genomic DNA was extracted from all individuals using the DNeasy Blood and Tissue kit (Qiagen) according to the manufacturer's instructions for extraction of DNA from insects. Agilent Tapestation 2200 was used to run Genomic DNA Screentape (Agilent Technologies) to assess DNA quality. Following DNA extraction and quality control, samples were stored at −80°C.

### 2.5 PCR, Cleanup and COI Barcode Sequencing

Samples of extracted DNA from all tested animals were diluted 1:10 in 2× UV treated nuclease free water. All samples were amplified using either the HCO1490/LCO2198 primer pair (Folmer et al. 1994) or the BF2/BR3 primer pair (Elbrecht et al. 2019; Appendix S3). For the former primer pair, a single touchdown PCR reaction was performed for each sample in a reaction volume of 25 μL (1X PCRBIO Ultramix (PCR Biosystems), 400 nM of each primer and 2 μL template DNA). The following thermocycler settings were used for the touchdown PCR: Initial denaturation at 95°C for 2 min followed by 16 cycles of: 95°C for 30 s, 62°C (−1°C each cycle) for 1 min, 72°C for 1 min, followed by 25 cycles of: 95°C for 30 s, 46°C for 1 min, 72°C for 1 min followed by a final extension at 72°C for 5 min. For the latter primer pair, a single PCR was performed for each sample in a reaction volume of 25 μL (1X PCRBIO Ultramix (PCR Biosystems), 400 nM of each primer and 2 μL template DNA). The following thermocycler settings were used for the PCR: Initial denaturation at 95°C for 2 min followed by 25 cycles of: 95°C for 30 s, 44°C for 1 min, 72°C for 45 s, followed by a final extension at 72°C for 5 min.

All samples were subsequently purified using the Monarch PCR & DNA Cleanup Kit (New England Biolabs) according to the manufacturer's instructions. DNA concentration of purified samples was determined using the Qubit HS dsDNA kit (Thermo Fisher Scientific) and a TECAN Infinite F200 Pro (Tecan Life Sciences), after which all samples that had successfully amplified were prepared for Sanger sequencing which was performed by Eurofins Genomics (Germany). Seventeen percent of samples (primarily Diptera) were either unsuccessfully amplified or resulted in a nonsense DNA‐sequence, and these were not analysed further.

### 2.6 Species Identification Using Barcoding

The obtained COI sequence for each animal was used for species identification using BLAST's blastn function (Camacho et al. 2009). An identity percentage above 98% and a report from at least two different accession numbers was considered reliable species identification. For sequences with an identity percentage lower than 98%, the highest identity was used following a visual comparison. Forty‐five samples necessitated visual confirmation and included individuals from 23 species, largely dominated by *Psyllidae* sp. (Appendix S3). Individuals from the species *Nysius groenlandicus* , *Nabis flavomarginatus*, and *Psammotettix lividellus* were identified to species level in the field (these species are easy to identify in the field based on morphological characteristics) and therefore largely excluded from imaging and sequencing. For the taxonomic analysis, one reference sequence from the BOLDsystems database was included in the analysis for each identified species, with a preference for sequences obtained from samples collected in sub‐Arctic regions. Every sample was manually curated by comparing recorded taxonomy to literature of local arthropod diversity (Böcher et al. 2015) under a principle of conservative taxonomic assignments. Subsequently, all assigned taxonomies were compared to our images of the sequenced animal (Böcher et al. 2015). In 49 cases, manual curation revealed obvious mismatches between images and recorded taxonomy, for example, wrong taxonomic order, and these samples were removed from further analysis. All sequences obtained were aligned using the MEGA software (Tamura et al. 2021) and the ClustalW algorithm with a gap opening quality of 15.00 and a gap extension penalty set at 6.66. Sequences that did not allow for alignment on account of short sequence length were removed from the analysis. All aligned sequences were shortened to consist of only a 410 bp region. Consensus sequences were generated from the aligned sequences obtained in this study using BioEdit (0.6). All phylogenetic trees were generated from aligned consensus sequences using MEGA software and the Neighbour‐joining tree function with 1000 bootstrap iterations.

### 2.7 Modelling Exposure to Microclimatic Temperatures Exceeding Thermal Thresholds

To investigate how the tested species may be affected by projected future temperatures, we compared species‐specific CT_max_ estimates to current microclimatic conditions, measured 15 cm above ground with TOMST loggers at 5 min intervals and averaged per hour, and projected future microclimatic temperatures. This height was chosen as this was where we mostly observed the animals in the vegetation during the day, and thus where we typically caught the animals. Using weather data from the National Centers for Environmental Prediction (van Heerwaarden and Sgrò 2021), hourly air temperatures were interpolated at the sampling location 2 m above the surface with the ‘NicheMapR’ package v3.2.1 (Kearney and Porter 2017) considering solar radiation, topology, soil properties, air and surface temperatures, and windspeed and ‐direction (Parratt et al. 2021; Bennett et al. 2021), in the period 19 July to 23 Aug 2023 (Ørsted et al. 2022). No reliable projections of future microclimates exist yet. Therefore, to obtain hourly future microclimate temperatures (*T*_micro_future_), we first calculated the difference in air temperatures between recent years (average monthly maximum and minimum temperatures 2000–2021) and projected at end‐of‐century conditions (2081–2100) under the SSP370 scenario (Figure S1). This difference, Δ*T*_air_, that is, future *T*_max_‐current *T*_max_ during daytime (8 AM–8 PM) and future *T*_min_‐current *T*_min_ during night, was added to the measured current microclimate (*T*_micro_current_) using the formula: *T*_micro_future_ = *T*_micro_current_ + *a*·e^(*b*·ΔTair)^, where *a* and *b* are coefficients for the exponential relationship between air and microclimate temperatures (different for day‐ and nighttime; Figure S3).

### 2.8 Statistical Analysis

Following species identification, outliers (for CT_max_ and CT_min_) within each species were identified and removed if having a median absolute deviation (MAD) of 3 or more, removing 53 samples (Leys et al. 2013). This was done to remove individuals with extremely low CT_max_ and high CT_min_ suggesting that they were harmed in the process of catching them and/or getting them into the test tube (all analyses were also performed without removing outliers and conclusions remained the same). Thermal scope was calculated for species with at least one measure of both CT_max_ and CT_min_ as the absolute difference between medians of bootstrap resampled data from each species using 10,000 iterations. Medians were used throughout on account of non‐normal distributions. Kruskal–Wallis tests were performed to compare medians within groups to determine potential differences. In case of significant differences, a post hoc Dunn's test was performed to compare groups. To investigate the presence of phylogenetic signals in each trait (CT_max_, CT_min_ and thermal scope), Pagel's Lambda was determined using the phytools R package running 1000 simulations (Revell 2024; Pagel 1999). Correlations between thermal tolerance traits were corrected for phylogenetic signals by performing Phylogenetic Generalized Least Squares (PGLS) models in the nlme R package (Pinheiro et al. 2024).

## 3 Results

### 3.1 Catch‐Test‐and‐Sequence Method Describes Thermal Limits for ~8% of Greenlandic Terrestrial Arthropods

COI barcode sequencing of the tested arthropods allowed us to obtain high resolution taxonomic information on 552 of the 701 collected animals (~79%) across 11 taxonomic orders (Table 1). A total of 99 species were described in this study comprising 8.25% of the described terrestrial arthropod fauna in Greenland (Böcher et al. 2015). CT_max_ and/or CT_min_ estimates were obtained for 93 of the recorded species with between 1 and 55 recorded individuals per species (Appendix S2). For six species we could not obtain measures of CT_min_ or CT_max_ either because they were physically too big to test in the vials that we had available ( *Apis mellifera* and *Vespula rufa*) or water entered the vials drowning the individuals during testing. The highest degree of species coverage was observed in the least diverse orders. For these orders, between 50% and 100% of the described Greenlandic terrestrial arthropods were observed in our study while the most diverse orders had a coverage between 8% and 15% in our data (Table 1). Thus, with the present work, we have established an inventory of thermal tolerance measures of south Greenlandic arthropods and a resource with photos and size measurements of investigated species for use in future studies.

**TABLE 1:** Coverage of thermal tolerances and species across taxonomic orders. All reference to Greenlandic (GL) arthropods is according to Böcher et al. (2015). % indicates percentage of described GL species found in the current study. Orders above 100% of described GL species occur because of lower taxonomic resolution for some individuals (e.g., Lamyctes sp.). See Appendix S2 for additional information.

Order	# Of described GL species	# And (%) of GL species recorded in current study	# Of species with CT_max_ measures obtained in current study	# Species with CT_min_ measures obtained in current study	# Of species where thermal scope could be calculated
Araneae	75	11 (15%)	9	7	5
Chilopoda	1	2 (200%)	2	0	0
Coleoptera	36 (native) 36 (introduced)	9 (25%) (native) 0 (introduced)	9	2	2
Diptera	~370	35 (10%)	27	17	10
Entomobryomorpha	2	1 (50%)	0	1	0
Hemiptera	41	11 (27%)	7	10	6
Hymenoptera	~200	15 (8%)	6	11	5
Lepidoptera	58	9 (16%)	8	4	4
Neuroptera	2	2 (100%)	1	1	0
Trichoptera	8	2 (25%)	0	2	0
Trombidiformes	2	2 (100%)	1	1	1
TOTAL	830^a^	99 (12%)	70	56	33

^a^ This number comprises the total number of Greenlandic species in the taxonomic orders described in this table and not the total number of described terrestrial arthropods in Greenland (which is ca. 1200).

### 3.2 Heat and Cold Tolerance Vary Drastically Within and Among Orders

**FIGURE 1:**
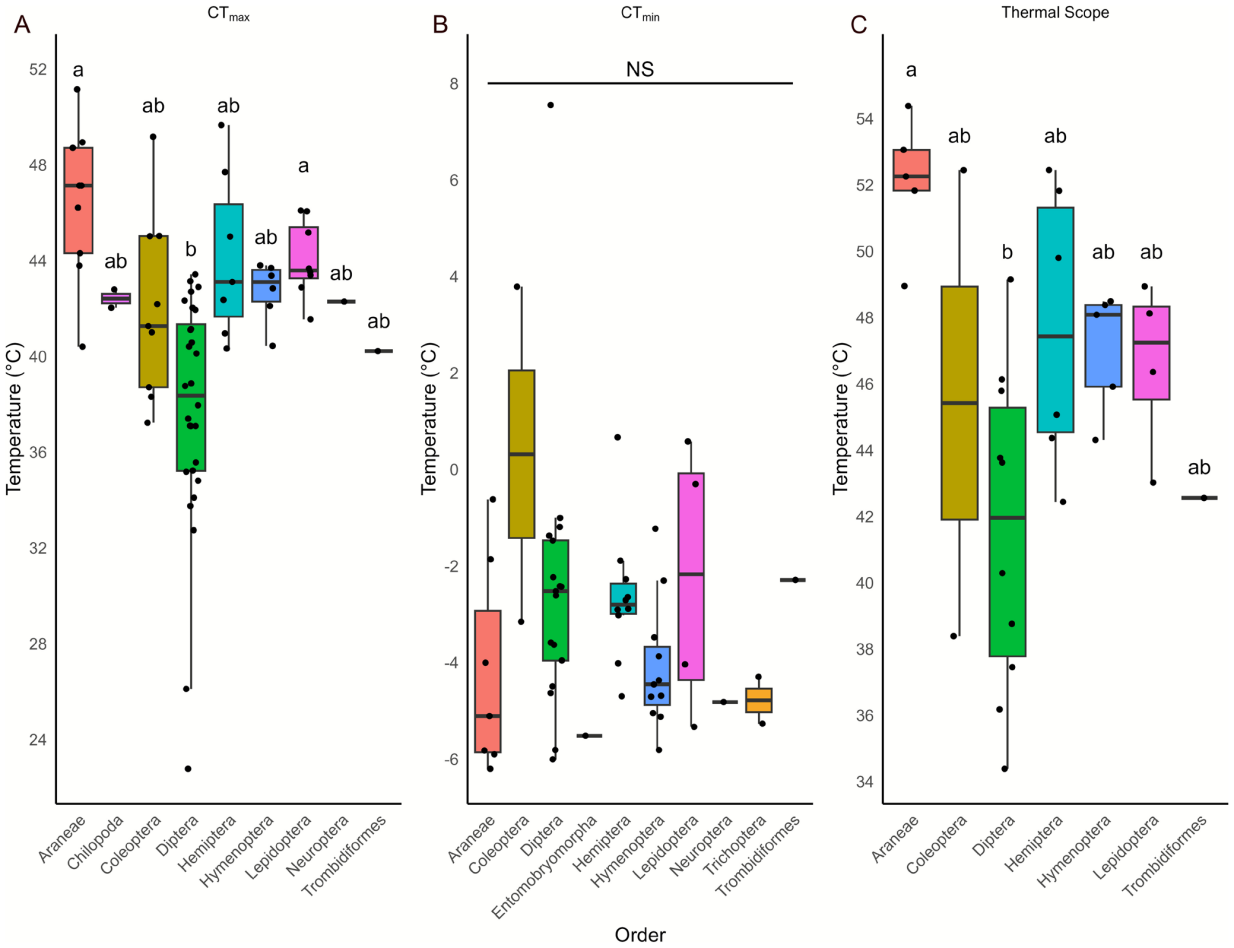
Boxplot of thermal stress tolerance measures for (A) CT_max_, (B) CT_min_ and (C) thermal scope for the different orders. Each black circle represents the median of a given species within the indicated order. Orders with shared letter denominators show no significant differences between them as per the post hoc Dunn's test.

We then tested the inter‐order differences of critical thermal limits and thermal scope and found that the median values of CT_max_ for each species differed significantly across taxonomic orders (Kruskal–Wallis, *Χ* ^2^ = 35.558, df = 8, *p* < 0.001; Figure 1A). The same was seen for thermal scope, which was also significantly different across orders (*Χ* ^2^ = 15.354, df = 6, *p* = 0.0177; Figure 1C). However, differences across orders were not observed for CT_min_ (*Χ* ^2^ = 12.969, df = 9, *p* = 0.164; Figure 1B).

**FIGURE 2:**
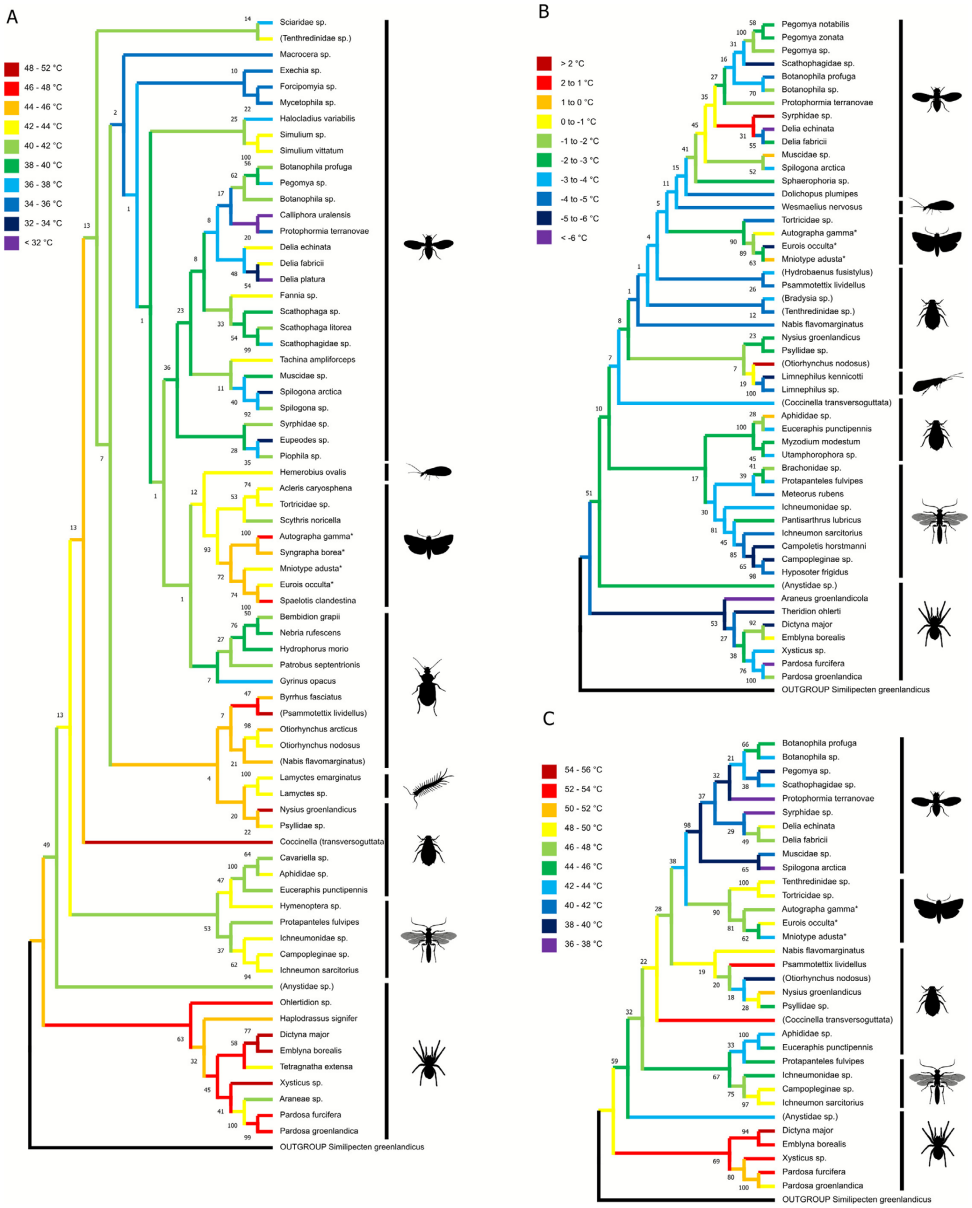
Neighbour‐joining Phylogenetic trees for (A) CT_max_, (B) CT_min_ and (C) thermal scope at species resolution level. Colours at branch tips indicate the median thermal limit or scope of the given species while colours of node branches represent mean of immediately connected branches. Species names in parentheses indicate phylogenetic placement outside taxonomic order. Species marked with a star indicate all individuals of the given species were recorded in the larval stage. Number by nodes are bootstrap values.

For CT_max_, Araneae ranked as having the highest median values across orders, while the lowest was observed for Diptera. A post hoc pairwise Dunns test revealed that Araneae and Lepidoptera had significantly higher median CT_max_ compared to species of Diptera (*p* = < 0.001 and *p* = 0.0028 respectively). Other orders did not differ in CT_max_ (Figure 1A). For thermal scope, Araneae and Diptera had the highest and lowest median values, respectively, and only these two were found to be significantly different (*p* = 0.0021; Figure 1C).

### 3.3 Strong Phylogenetic Signals for Upper Thermal Limits and Thermal Scope in Arctic Arthropods

We found a disparity between phylogenetic groups in CT_max_ and thermal scope (Figure 2A, C). This corresponded with the results reported above showing that Araneae had the highest CT_max_, with some species having individuals still being active at temperatures above 50°C. In contrast, dipterans had the lowest CT_max_, typically below 35°C. The comparatively higher CT_max_ for lepidopterans seen in Figure 1, was also observed here across species (and life stages; Figure 2A). For CT_min_, no apparent trend was observed (Figure 2B), which aligned with results showing no significant effect of order for CT_min_ (Figure 1B). Finally, for thermal scope (Figure 2C), Araneae species had the highest values, while Lepidoptera had a lower measure of thermal scope, and the dipterans had the lowest values. To test for phylogenetic signal in each of these thermal limit measures, Pagel's *λ* was calculated, which revealed a strong and highly significant phylogenetic signal for thermal scope (*λ* = 0.99, *p* < 0.001) and CT_max_ (*λ* = 0.49, *p* < 0.001), suggesting that closely related species have a more similar thermal scope and CT_max_ than more distantly related species. In contrast, no phylogenetic signal was observed for CT_min_ (*λ* < 0.001, *p* = 1).

**FIGURE 3:**
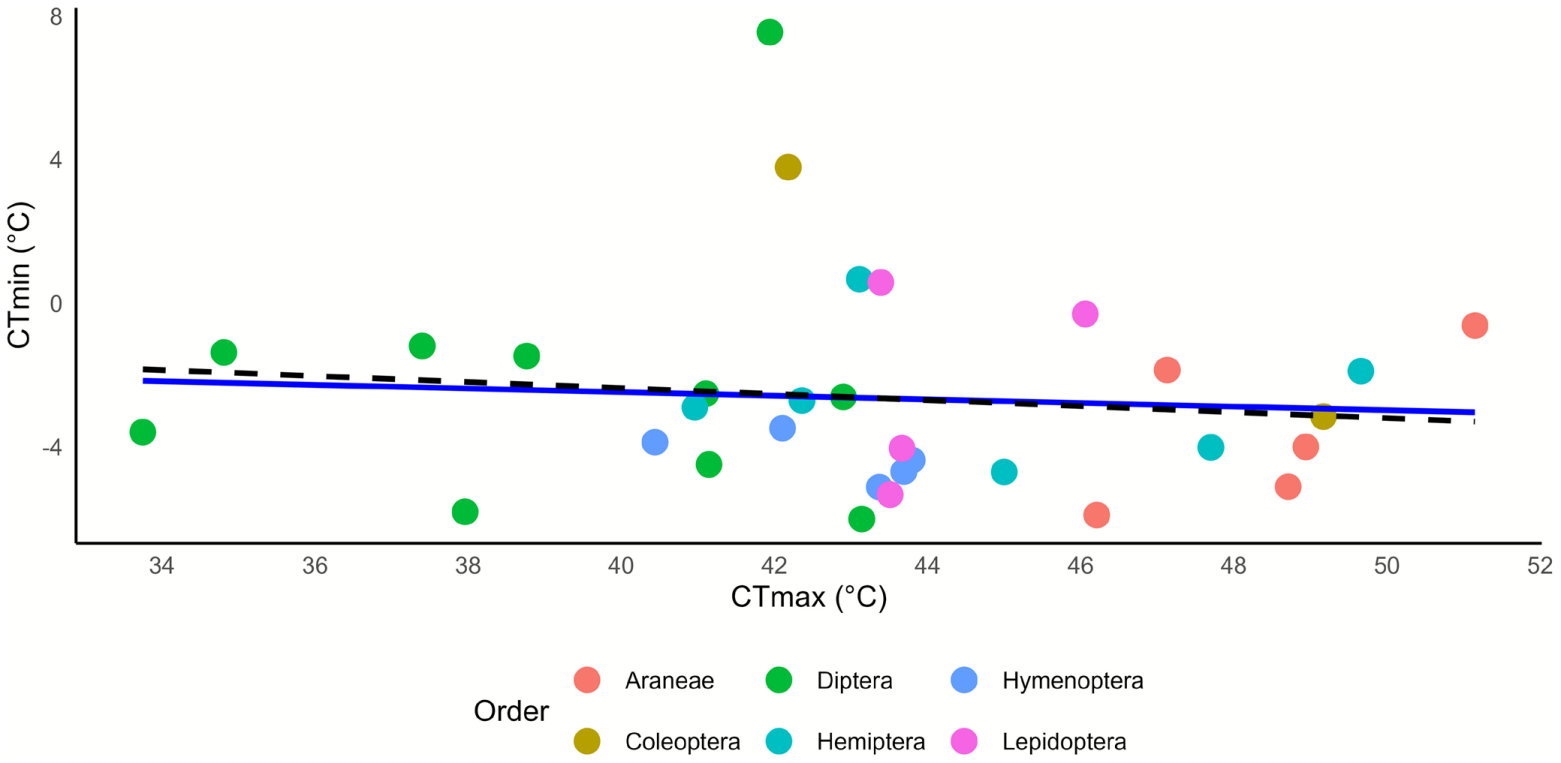
Correlation analyses of thermal limits. Each dot represents the median of a species in an order designated by the colour of the dot. The solid line represents a linear model not accounting for phylogeny, while the dashed line represents a Phylogenetic Generalized Least Squares (PGLS) model.

### 3.4 Independent Evolution of Upper and Lower Thermal Limits

A Phylogenetic Generalized Least Squares model (PGLS) revealed no correlation between CT_min_ and CT_max_ with a slope of −0.08 (SE = 0.01, *t* = −0.77, *p* = 0.44; Figure 3), indicating independent evolution of the two traits. Additionally, no significant relationships were observed between the medians of length of specimens (body size) and both CT_max_ and CT_min_ at the species level (*R* ^2^ < 0.001, *p* = 0.89 and *R* ^2^ = 0.010, *p* = 0.47), respectively.

### 3.5 Projected Future Microclimatic Temperatures May Exceed Upper Thermal Tolerance Limits

**FIGURE 4:**
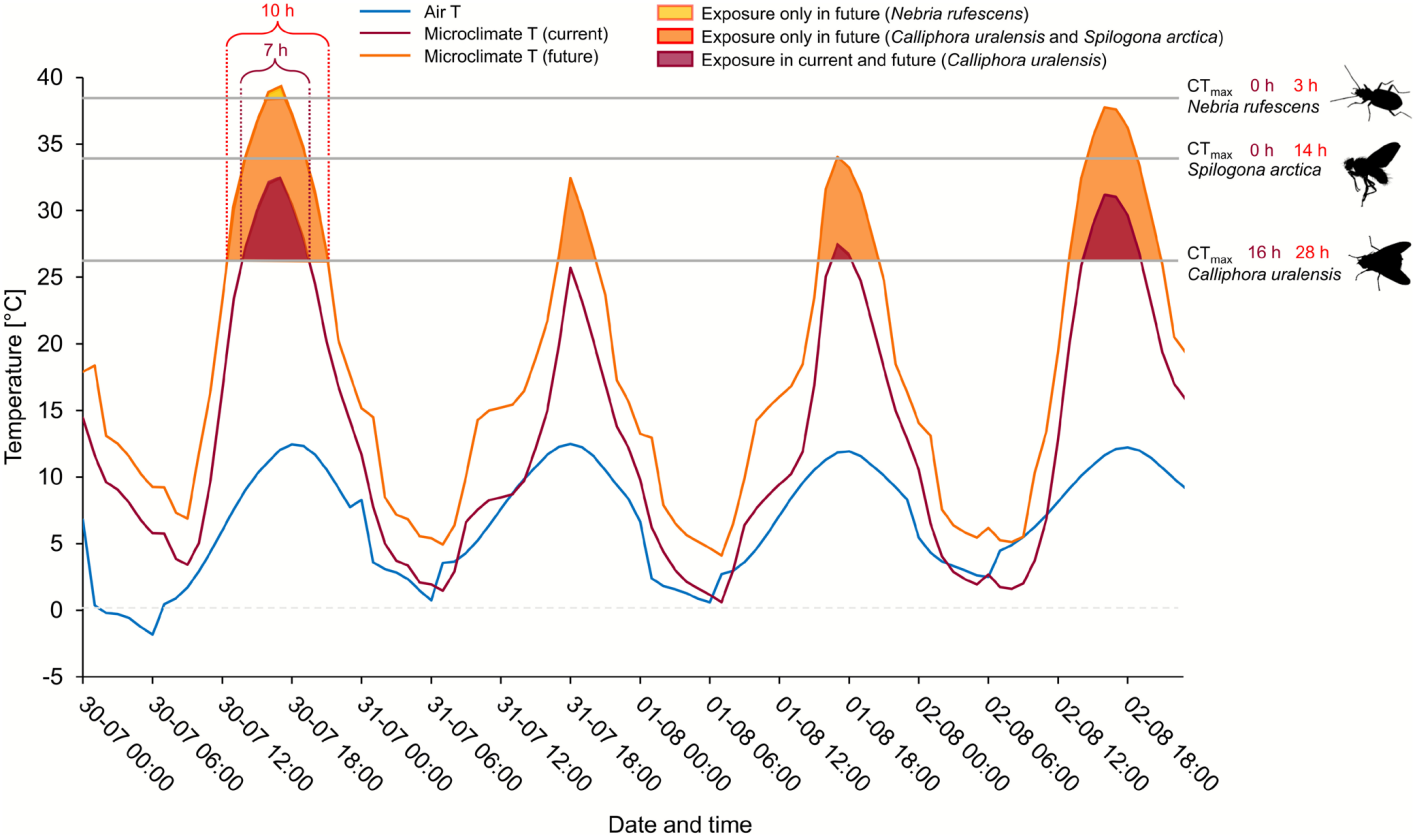
Example of exposure of three study species ( *Calliphora uralensis* , Spilogona arctica, and Nebria rufescens) to microclimatic temperatures exceeding their CT_max_ (grey horizontal lines). Solid lines represent air temperature (blue) and microclimate temperatures under current conditions (maroon: Hourly average obtained from TOMST loggers) and projected future conditions (red, see methods and Figures S1–S3) in the period 30 July to 2 August 2023. During these four days, exposure to temperatures above CT_max_ is highlighted under current and future conditions (maroon and red areas, respectively), while *N. rufescens* is only exposed to T>CT_max_ under future microclimatic conditions (yellow area). The duration of T>CT_max_ for *C. uralensis* is shown as an example highlighting that not only are some species going to experience more days with temperatures above CT_max_, but the duration of exposure will also lengthen (from 7 to 10 h). The sum of exposure to temperatures above CT_max_ for the exemplary 4 days is shown under current and predicted future conditions.

Observed hourly microclimate temperatures measured in 2023 far exceeded air temperatures and predicted temperatures based on microclimatic models (Kearney and Porter 2017) for the same area and period (Figure 4, Table S1). While the microclimatic model was able to predict a similar mean temperature for the investigated period between 19 July to 23 Aug 2023, it failed in achieving the extreme temperature peaks during both night and day (Table S1), and thus, neither air temperature nor microclimatic models were sufficient in describing the temperatures experienced by arthropods in the area.

Additionally, temperatures measured at 2–5 cm in height appeared to be higher than those measured at 15 cm (Table S1). Using CT_max_ estimates obtained in this study, we showed that during the entire summer period of 2023 two species ( *Delia platura* and *Calliphora uralensis* ) were likely to have experienced microclimate temperatures above their CT_max_ (Table S2). During this period, these stressful temperatures occurred regularly for the two species at 89% and 77% of days, respectively.

When associating the CT_max_ measures to the projected microclimatic temperatures (end‐of‐century SSP370 scenario), the number of species that will potentially experience temperatures above their upper thermal limit will increase dramatically to 18 (25% of species with CT_max_ estimates in our study) and on average they will be exposed to potentially stressful temperatures (defined as time spent exposed to temperatures above their estimated CT_max_) 35.83% of days across the investigated period (Table S2).

## 4 Discussion

In this study, we investigated thermal tolerances of a wide range of terrestrial arthropods from Narsarsuaq in Southern Greenland. Our results (1) contribute to reducing the knowledge gap on thermal tolerance limits in the Greenlandic terrestrial arthropods, (2) highlight the power of incorporating molecular species identification in arthropod field surveys, (3) suggest evidence of evolutionary constraints in heat tolerance and thermal scope in Arctic arthropods, and (4) reveal that periodically, a quarter of the investigated species are likely to experience temperatures above their CT_max_ under predicted future microclimatic conditions.

By combining rapid thermal tolerance measurements with COI barcoding, we provide a framework for rapid biodiversity and eco‐physiological field assessments. Using this method, we offer new insights into the capacity of 93 Greenlandic arthropod species to withstand low and high temperatures constituting ~8% of the described Greenlandic terrestrial arthropod species diversity. This also represents a significant increase in the number of arthropod species where information on thermal tolerances is available globally (Bennett et al. 2018). Although molecular methods for species identification are routinely used (Takahashi et al. 2023; Creer et al. 2016; Parker et al. 2017), we propose that there is an unutilized potential in incorporating barcoding approaches with phenotypic and physiological data for a wide range of species and in different contexts. This combination offers the possibility of covering much of the knowledge gap with relatively little effort, as in this case, where the field work was performed within a week. This is particularly important when working with limited resources or in areas with adverse working conditions or a short growing season, as is our case in a polar region (Hansen et al. 2017).

We observed a clear and significant trend that members of the order Araneae had both the highest CT_max_ and the lowest CT_min_. Alongside Araneae, Lepidoptera species were also found to have a high CT_max_, but this was mainly driven by the larval individuals from the Noctuidae family, which have previously been shown to have higher CT_max_ in the larval stage as compared to the adult life stage (Bawa et al. 2021). Bahrndorff et al. (2021) reported a CT_max_ value for adult *Eurois occulta* which was 3°C lower than reported here, while presenting comparable values for several other species across different orders also tested here including *N. groenlandicus, P. lividellus* and *N. flavimarginatus* (Bahrndorff et al. 2021). For the genus *Pardosa*, we obtained higher estimates of CT_max_ and lower estimates of CT_min_ compared to findings in Anthony et al. (2019). Lepidoptera and Araneae typically have high upper thermal limits with estimates in the literature in the range 45°C–50°C (Bawa et al. 2021; Chidawanyika and Terblanche 2011) and 42°C–49°C (Anthony et al. 2019; Hanna and Cobb 2007), respectively. Our results for these taxa were in alignment with these findings and generally upper thermal limits across the other taxonomic orders correspond to what has previously been reported in the literature. A general finding in our study was that Diptera had low and variable heat tolerance with CT_max_ values in the range 22°C–43°C. This aligned with literature findings (Hoffmann et al. 2023; Bahrndorff et al. 2021; Weaving et al. 2023; Lighton 2007; Folk et al. 2007; Leclerc et al. 2022) highlighting the large intra‐order variability and the importance of high taxonomic resolution in thermal tolerance studies. Likewise, Coleoptera and Hymenoptera have been shown to have CT_max_ between 38°C and 45°C (Jones et al. 2021; Käfer et al. 2012; Oyen et al. 2016), aligning with our findings. These results also revealed that, although various taxa have diverged in their upper thermal limits, such divergence is not evident for lower thermal limits. One explanation for this may be the extreme low temperatures that all arthropods have evolved to cope with for hundreds of thousands of years in this region (Vasskog et al. 2015), where cold tolerance has been a limiting factor for arthropod distribution and therefore under strong selection. In comparing and interpreting CT_max_ and CT_min_ values across studies, it is however important to be aware that conditions including ramping rates, start temperature and pretest rearing temperatures can have a marked impact on the estimates (Overgaard et al. 2012; Sørensen et al. 2012). The ramping rate used in this study (0.1°C per minute) was chosen to be so fast that it reduced potential hardening effects but also slow enough to avoid marked discrepancies between water temperature and animal body temperature. Additionally, microclimate temperatures in our study area increased during the warmest parts of the day at a rate of 0.1°C per minute or more, thus we argue that the chosen rate is ecologically relevant (Appendix S1). Typically, the slower the ramping rate the lower the estimated CT_max_ (Sørensen et al. 2012), likely because at a given temperature, slow compared to fast ramping leads to more cellular damage because animals are experiencing longer periods at stressful temperatures prior to reaching CT_max_. Thus, if we had used a slower ramping rate, lower CT_max_ values would have been expected, and this would have led to an even more concerning projected future scenario in which a larger number of species could potentially face stressful high temperatures. It is also important to consider the measures of CT_min_ and CT_max_ recorded in this study in the context of the sampling season. That is, all individuals presented here were collected and tested during the hottest period of the year. This would likely be reflected in the recorded cold and heat tolerance measures. The importance of this is likely highest for CT_min_ as this trait is considered to be relatively more plastic than upper thermal limits in arthropods, and capable of changing significantly, both seasonally and daily, depending on the environment (Böcher et al. 2015; Noer et al. 2022; Schou et al. 2017). Further, even though CT_min_ values might be different (likely lower) during winter for the investigated species our estimates still provide much needed insight into cold tolerances of a wide taxonomic range of arthropods.

Estimates of upper thermal limits and thermal scope obtained in this study were highly dependent upon the phylogenetic relationship across the tested species (Figure 2A,C), meaning that closely related species had more similar measures of upper thermal tolerance than distantly related species. This result raises the question of whether upper thermal tolerance and thermal scope in Arctic arthropods are constrained in their capacity to adapt evolutionarily to higher temperatures. While a strong phylogenetic signal can be indicative of evolutionary constraints, it is important to consider the large taxonomic range included in this study (da Silva et al. 2023). Closely related species with very different modes of life and thus different life histories (Conti et al. 2023; Vives‐Ingla et al. 2023) like the brush and herb‐living mesh‐weavers ( *Dictyna major* and *Emblyna borealis* ) and the ground‐dwelling wolf spiders ( *Pardosa groenlandica* and *Pardosa furcifera* ; Böcher et al. 2015) in our study were revealed to have almost identical upper thermal limits. This similarity may be driven by either evolutionary constraints or shared evolutionary history. Multiple studies have found evolutionary constraints in upper thermal limits for a wide range of taxonomic groups (Kellermann et al. 2012; Hoffmann et al. 2013; Sandblom et al. 2016). High stressful temperatures during summer are likely a relatively novel environmental stress in Greenland and thus not a trait that has been under strong selection. We therefore speculate that genetic variation for the ability to cope with heat stress has been lost due to for example DNA decay, which has been suggested as a process occurring when a gene is no longer under selection and accumulates mutations, and also when there is direct selection for loss of function (McBride 2007; Hoffmann and Willi 2008). Alternatively, the similar thermal limits may reflect shared life‐history traits; even if adults occupy different microclimates, other life stages may not, thereby imposing similar thermal requirements. More research on the implication of the observed phylogenetic signal found in this study is needed, but we argue that the observed phylogenetic signal could represent evolutionary constraints.

Finally, by projecting future diurnal microclimatic temperatures according to climate predictions, we highlight the issue of increased exposure to stressful warm microhabitat temperatures for arthropods from southern Greenland. A quarter of the tested species reported here will potentially experience future temperatures periodically exceeding their upper critical thermal limit. Although behavioural thermoregulation and plasticity may protect arthropods against future warmer and more variable temperatures (Koltz et al. 2018), these adaptations typically come with a behavioural and metabolic cost, resulting in a potential decrease in the fitness of the individuals (Ma et al. 2018). Additionally, studies on species from other latitudes reveal that physiological adjustments (i.e., plasticity) can be highly maladaptive in rapidly changing and unpredictable thermal environments (Kristensen et al. 2008). Studies have also shown that sublethal temperatures markedly below and above CT_max_ and CT_min_, respectively, can result in significantly reduced fitness like male infertility which has been shown to be reliable predictors of changes in species distribution (van Heerwaarden and Sgrò 2021; Parratt et al. 2021; Ørsted et al. 2024). The recently re‐introduced Thermal Death Time (TDT) framework (Ørsted et al. 2022; Jørgensen et al. 2021; Rezende et al. 2014; Molina et al. 2024), describes how thermal damage accumulates exponentially with exposure time, suggesting that CT_max_ and CT_min_ represent the absolute upper and lower thermal limits and that individuals can suffer potentially lethal heat stress at temperatures well below their CT_max_, and above CT_min_ thresholds, respectively. Thus, employing more advanced descriptions of the thermal landscape of Arctic arthropods represents an interesting avenue for further exploration, especially since, to our knowledge, these TDT relationships have not been characterized for any Arctic arthropods yet (Vives‐Ingla et al. 2023; Sandblom et al. 2016; McBride 2007). Using this sort of framework to estimate other thermal limits, for example, the temperature resulting in thermal failure in 1 h (which is lower than CT_max_) as employed in other studies (Ørsted et al. 2022, 2025; Jørgensen et al. 2021), or including CT_max_/CT_min_ values obtained at lower ramp rates, would likely have resulted in a larger number of species being expected to experience temperatures exceeding their thermal limits. This suggests that our estimate based on CT_max_ measures, revealing that upwards of a quarter of the investigated species will likely suffer from increasing future summer temperatures in Southern Greenland, is a conservative estimate. This is also supported by the fact that the microclimatic temperature model is based on a conservative estimation, as the temperature at 15 cm height was observed to be among the lowest extreme temperature values measured in the area both in 2023 and 2018 (Table S1).

We set up our microclimate temperature measurements to best represent the microspatial heterogeneity in the area. However, we are likely not capturing all the potential local extremes or variations. Nevertheless, since all arthropods are collected from the grass across the same area, we believe that the presented microclimate measurements constitute representative measures for the species assessed here. Whether these findings are representative for other Arctic regions and microhabitats requires further studies.

Here we contribute to markedly expanding the number of arthropod species for which we have information on thermal stress tolerance. Other considerable and timely efforts have been made to understand the thermal limits for a range of taxonomic groups and the ecological consequences thereof (Kellermann et al. 2012; Bennett et al. 2018; Bahrndorff et al. 2025; Pottier et al. 2022; Diamond et al. 2024). Such studies are essential to expanding our limited knowledge base, but it is of equal importance to coordinate a joint effort to integrate all this information into large, united databases, as information is currently scattered and hard to access for researchers and practitioners. To enable more powerful analyses that reveal trends not only locally but also globally and over longer time scales, it is essential to ensure that data are comparable and easily accessible. This would be highly valuable for future studies aiming at predicting changes in diversity, abundance, and distribution of arthropods in a changing climate and how this will impact complex ecological networks.

## 5 Conclusion

Changes in the abundance and distribution of Arctic terrestrial arthropods are important to understand because these taxa play key ecological roles, including pollination, nutrient cycling, seed dispersal (Eggleton 2020), and acting as pests or disease vectors for plants, animals and even humans (Gubler 1998; Ng and Perry 2004). Our study provides important insights into the thermal tolerances of a considerable proportion of Greenlandic arthropods (93 out of the estimated 1200 species) and reveals clear phylogenetic signals for both CT_max_ and thermal scope. We propose that the phylogenetic signal we observe suggests an evolutionary constraint on these traits. Together with our finding that a quarter of the species will likely experience microclimates exceeding their upper thermal limits for extended periods, this indicates that climate change will have profound impacts on Arctic arthropods. Future studies should examine whether the patterns identified here are consistent across Arctic regions and seasons, and the approach we use provides a useful framework for achieving this.

## Author Contributions

**Jonas Bruhn Wesseltoft:** conceptualization, data curation, formal analysis, investigation, methodology, visualization, writing – original draft, writing – review and editing. **Nadieh de Jonge:** formal analysis, methodology, writing – review and editing. **Michael Møller Hansen:** methodology, writing – review and editing. **Toke Thomas Høye:** writing – review and editing. **Michael Ørsted:** formal analysis, funding acquisition, investigation, supervision, visualization, writing – original draft, writing – review and editing. **Torsten Nygaard Kristensen:** conceptualization, formal analysis, investigation, methodology, project administration, supervision, writing – original draft, writing – review and editing.

## Funding

The work was funded by the European co‐funded Partnership BiodivClim‐191 ASICS (0156‐00024B to TNK), and by the Novo Nordisk Foundation (grant NNF23OC0082599 to MØ). Laboratory facilities in Narsarsuaq, Greenland, were supported by the Greenland Integrated Observing System (GIOS) (the Danish National Fund for Research Infrastructure (NUFI)).

## Disclosure

Licenses: All animals were collected in accordance with regulations from the government of Greenland under the non‐exclusive license no. G23‐006 for utilization of Greenland genetic resources as granted to Aalborg University for use between 01/05‐2023 until 31/09‐2023.

## Conflicts of Interest

The authors declare no conflicts of interest.

## Supporting information


**Appendix S1:** Microclimatic temperature profile for Narsarsuaq.

**Appendix S2:** Registry of identified taxons and representative images.

**Appendix S3:** Raw data for thermal tolerances and COI sequences.

**Appendix S4:** gcb70687‐sup‐0004‐AppendixS4.docx.

## Data Availability

The data that support the findings of this study are openly available in Dryad at (https://doi.org/10.5061/dryad.1g1jwsv6w) and Figshare at (https://doi.org/10.6084/m9.figshare.28435652.v2).
