# Supplementary material for: Heat but Not Cold Tolerance Is Phylogenetically Constrained in Greenlandic Terrestrial Arthropods Under Future Global Warming
Source: Glob Chang Biol. 2026 Jan 8;32(1):e70687. doi: 10.1111/gcb.70687 (PMC12783425; doi:10.1111/gcb.70687)

Table S 1: CTmax, CTmin, thermal scope and length measures of all species of which at least one measure of thermal limit was recorded. Asterisk indicate species of which image was not obtained. No SE is noted for single measures.

Species	Order	Count	CTMax (Median + SE) (˚C)	CTmin (Median + SE) (˚C)	Scope (Median + SE) (˚C)	Length (Median + SE) (mm)
Araneae sp.	Araneae	2	40.4 ± 5.4	--	--	4.85 ± 0.35
Araneus groenlandica	Araneae	1	--	- 6.2	--	3.3
Dictyna major	Araneae	11	49.05 ± 0.26	- 5.11 ± 0.34	54.39 ± 0.68	2.1 ± 0.2
Emblyna borealis	Araneae	5	51.21 ± 0.09	- 0.62 ± 0.73	51.83 ± 0.52	1.2 ± 0.1
Haplodrassus signifer	Araneae	1	44.31	--	--	8.5
Ohlertidion sp.	Araneae	1	47.13	--	--	2.8
Pardosa furcifera	Araneae	3	46.21	- 6.05 ± 0.15	52.26 ± 0.10	8.0 ± 0.60
Pardosa groenlandica	Araneae	4	47.09 ± 0.04	- 1.86	48.95 ± 0.05	9.65 ± 0.74
Tetragnatha extensa	Araneae	1	43.79	--	--	8.5
Theridion ohlerti	Araneae	1	--	- 5.82	--	2.5
Xysticus sp.	Araneae	27	49.08 ± 0.25	- 4.0 ± 0.41	53.08 ± 0.54	3.45 ± 0.27
Lamyctes emarginatus	Chilopoda	1	42.03	--	--	6.5
Lamyctes sp.	Chilopoda	2	42.8 ± 0.44	--	--	6.1 ± 0.4
Bembidion grapii	Coleoptera	3	40.97 ± 0.02	--	--	4.2 ± 0.12
Byrrhus fasciatus	Coleoptera	1	45.02	--	--	7.0
Coccinella transversoguttata	Coleoptera	4	49.3 ± 0.13	-3.16 ± 0.12	52.45 ± 0.12	5.55 ± 0.26
Gyrinus opacus	Coleoptera	3	37.23 ± 0.2	--	--	6.0 ± 0.37
Hydrophorus morio*	Coleoptera	1	38.71	--	--	3.8
Nebria rufescens	Coleoptera	3	38.31 + 0.0	--	--	5.50 ± 0.17
Otiorhynchus arcticus	Coleoptera	2	45.04 ± 0.0	--	--	6.55 ± 0.45
Otiorhynchus nodosus	Coleoptera	3	42.18 ± 0.0	3.79	38.39 ± 0.0	8.0 ± 0.17

Patrobus septentrionis	Coleoptera	2	41.27 ± 1.2	--	--	8.5 ± 0.5
Botanophila profuga	Diptera	9	41.23 ± 0.1	-4.56 ± 0.07	45.79 ± 0.11	5.4 ± 0.22
Botanophila sp.	Diptera	9	41.11 ± 0.38	-2.52 ± 1.52	43.63 ± 2.15	4.95 ± 0.13
Bradysia sp.	Diptera	1		-3.63		1.2
Calliphora uralensis	Diptera	2	26.12 ± 3.85			13.5 ± 0.5
Delia echinata	Diptera	2	43.14	-6.01	49.15 ± 0.00	4.45 ± 0.05
Delia fabricii	Diptera	33	42.92 ± 0.14	-3.18 ± 0.52	46.12 ± 0.52	6.20 ± 0.13
Delia platura	Diptera	1	22.78	--	--	4.20
Dolichopus groenlandicus	Diptera	1	--	-1.00	--	5.10
Dolichopus plumipes	Diptera	1	--	-4.63	--	5.30
Eupeodes sp.	Diptera	1	32.75	--	--	9.50
Exechia sp.	Diptera	1	35.57	--	--	4.0
Fannia sp.	Diptera	1	42.70	--	--	4.0
Forcipomyia sp.	Diptera	1	35.23	--	--	2.1
Halocladius variabilis	Diptera	3	37.10 ± 1.74	--	--	3.1 ± 0.33
Hydrobaenus fusistylus	Diptera	1	--	-3.96	--	3.0
Macrocera sp.	Diptera	1	34.10	--	--	4.7
Muscidae sp.	Diptera	16	38.82 ± 0.22	- 1.47 ± 2.91	40.29 ± 4.47	5.0 ± 0.20
Mycetophila sp.	Diptera	1	35.18	--	--	4.7
Pegomya notabilis	Diptera	2	--	-2.24 ± 0.26	--	6.75 ± 0.75
Pegomya sp.	Diptera	4	37.40	-1.36 ± 0.44	38.76 ± 0.56	5.40 ± 0.27
Pegomya zonata	Diptera	1	--	-2.43	--	5.80
Protophormia terranovae	Diptera	6	34.81 ± 1.08	-1.37 ± 1.1	36.18 ± 1.43	10.00 ± 1.08
Scathophaga litorea	Diptera	1	40.12	--	--	7.0
Scathophaga sp.	Diptera	2	38.82 ± 0.06	--	--	6.4 ± 0.6
Scathophagidae sp.	Diptera	2	37.96	-5.81	43.77 ± 0.00	5.4 ± 0.6
Sciaridae sp.	Diptera	2	37.09 ± 0.07	--	--	2.05 ± 0.05

Simulium sp.	Diptera	5	42.33 ± 0.39	--	--	2.70 ± 0.09
Simulium vittatum	Diptera	2	42.03 ± 0.26	--	--	2.55 ± 0.25
Sphaerophoria sp.	Diptera	1	--	-2.42	--	10.0
Spilogona arctica	Diptera	3	33.75	-3.71 ± 0.12	37.46 ± 0.08	5.50 ± 0.54
Spilogona sp.	Diptera	1	40.41 ± 0.11	--	--	5.30 ± 1.00
Syrphidae sp.	Diptera	4	41.94 ± 0.63	7.55	34.39 ± 0.8	7.50 ± 0.29
Tachina ampliforceps	Diptera	2	43.43 ± 0.44	--	--	16.0 ± 3.0
Lepidocyrtus sp.	Entomobryomorpha	1	--	-5.52	--	1.50
Aphididae sp.	Hemiptera	2	43.11	0.67	42.44 ± 0.00	1.75 ± 0.25
Cavariella sp.	Hemiptera	4	40.33 ± 0.05	--	--	1.50 ± 0.15
Euceraphis punctipennis	Hemiptera	7	40.96	-3.41 ± 0.28	44.37 ± 0.49	2.80 ± 0.18
Euceraphis sp.	Hemiptera	1	--	-2.89	--	3.90
Myzodium modestum	Hemiptera	1	--	- 2.65	--	1.70
Nabis flavomarginatus	Hemiptera	41	45.07 ± 0.2	-4.72 ± 0.14	49.80 ± 0.32	8.0 ± 0.09
Nysius groenlandicus	Hemiptera	50	49.62 ± 0.09	-2.14 ± 0.22	51.82 ± 0.33	3.90 ± 0.05
Psammotettix lividellus	Hemiptera	14	48.19 ± 0.18	-4.28 ± 0.38	52.45 ± 0.45	3.30 ± 0.1
Psyllidae sp.	Hemiptera	10	42.36 ± 0.28	-2.71	45.07 ± 0.26	1.80 ± 0.11
Pterocomma sp.	Hemiptera	1	--	-2.27	--	2.30
Utamphorophora sp.	Hemiptera	1	--	-3.02	--	2.0
Brachonidae sp.	Hymenoptera	1	--	-1.23	--	2.50
Campoletis horstmanni	Hymenoptera	1	--	-5.81	--	6.5
Campopleginae sp.	Hymenoptera	3	43.37	5.12 ± 1.09	48.49 ± 0.77	5.0 ± 0.52

Hymenoptera sp.	Hymenoptera	1	42.84	--	--	2.5
Hyposoter frigidus	Hymenoptera	2	--	-4.45 ± 0.57	--	6.05 ± 0.25
Hyposoter sp.	Hymenoptera	1	--	-5.05	--	9.0
Ichneumon sarcitorius	Hymenoptera	9	43.80 ± 0.06	-4.37 ± 0.70	48.17 ±0.85	12.0 ± 0.17
Ichneumonidae sp.	Hymenoptera	5	45.91 ± 0.61	-3.81 ± 0.47	42.11 ± 0.25	5.0 ± 0.1
Meteorus rubens	Hymenoptera	1	--	-4.71	--	4.0
Pantisarthrus lubricus	Hymenoptera	1	--	-2.30	--	4.50
Protapanteles fulvipes	Hymenoptera	2	40.44	-3.87	44.31 ± 0.00	3.60 ± 0.40
Tenthredinidae sp.	Hymenoptera	8	43.69 ± 0.33	- 4.69	48.38 ± 0.53	4.70 ± 0.46
Acleris caryosphena	Lepidoptera	1	42.88	--	--	7.0
Autographa gamma	Lepidoptera	2	46.06	-0.30	46.36 ± 0.00	15.50
Eurois occulta	Lepidoptera	9	43.51 ± 0.30	-5.43 ± 0.1	48.94 ± 0.58	4.80 ± 0.57
Mniotype adusta	Lepidoptera	4	43.60 ± 0.48	0.58	43.02 ± 0.61	12.00 ± 1.68
Scythris noricella	Lepidoptera	2	41.55 ± 8.9	--	--	6.10 ± 0.1
Spaelotis clandestina	Lepidoptera	1	46.09	--	--	18.0
Syngrapha borea	Lepidoptera	1	45.17	--	--	3.60
Tortricidae sp.	Lepidoptera	44	43.73 ± 0.29	-4.38 ± 0.27	48.13 ± 0.47	7.0 ± 0.13
Hemerobius ovalis	Neuroptera	1	42.29	--	--	5.80
Wesmaelius nervosus	Neuroptera	1	--	-4.82	--	4.70
Limnephilus kennicotti	Trichoptera	1	--	-5.27	--	7.0
Limnephilus sp.	Trichoptera	2	--	-4.3 ± 0.11	--	10.0 ± 0.0
Anystidae sp.	Trombidiformes	4	40.22	-2.34 ± 0.42	42.56 ± 0.55	1.15 ± 0.05

Araneae

Araneae sp.

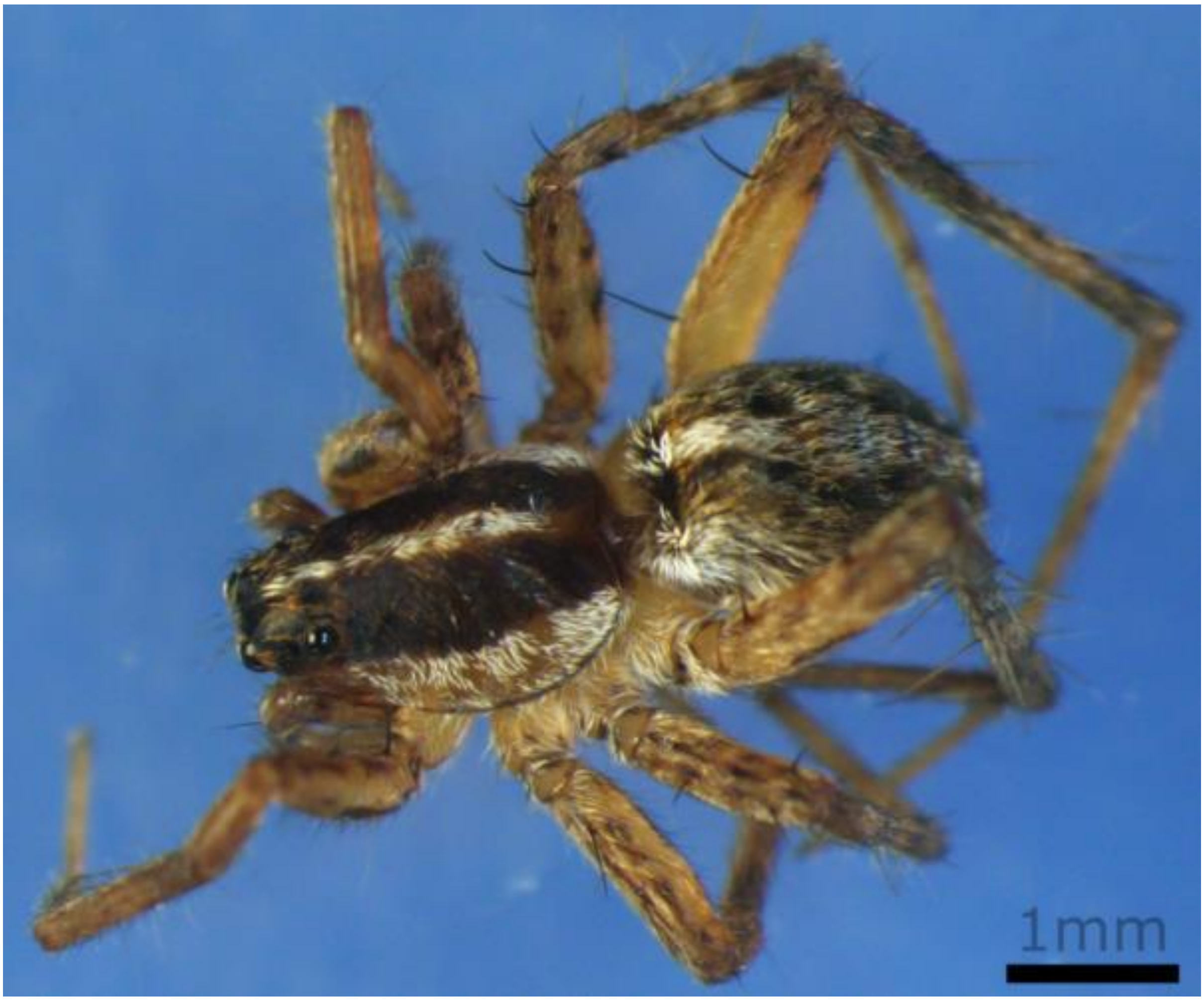

Araneus groenlandica

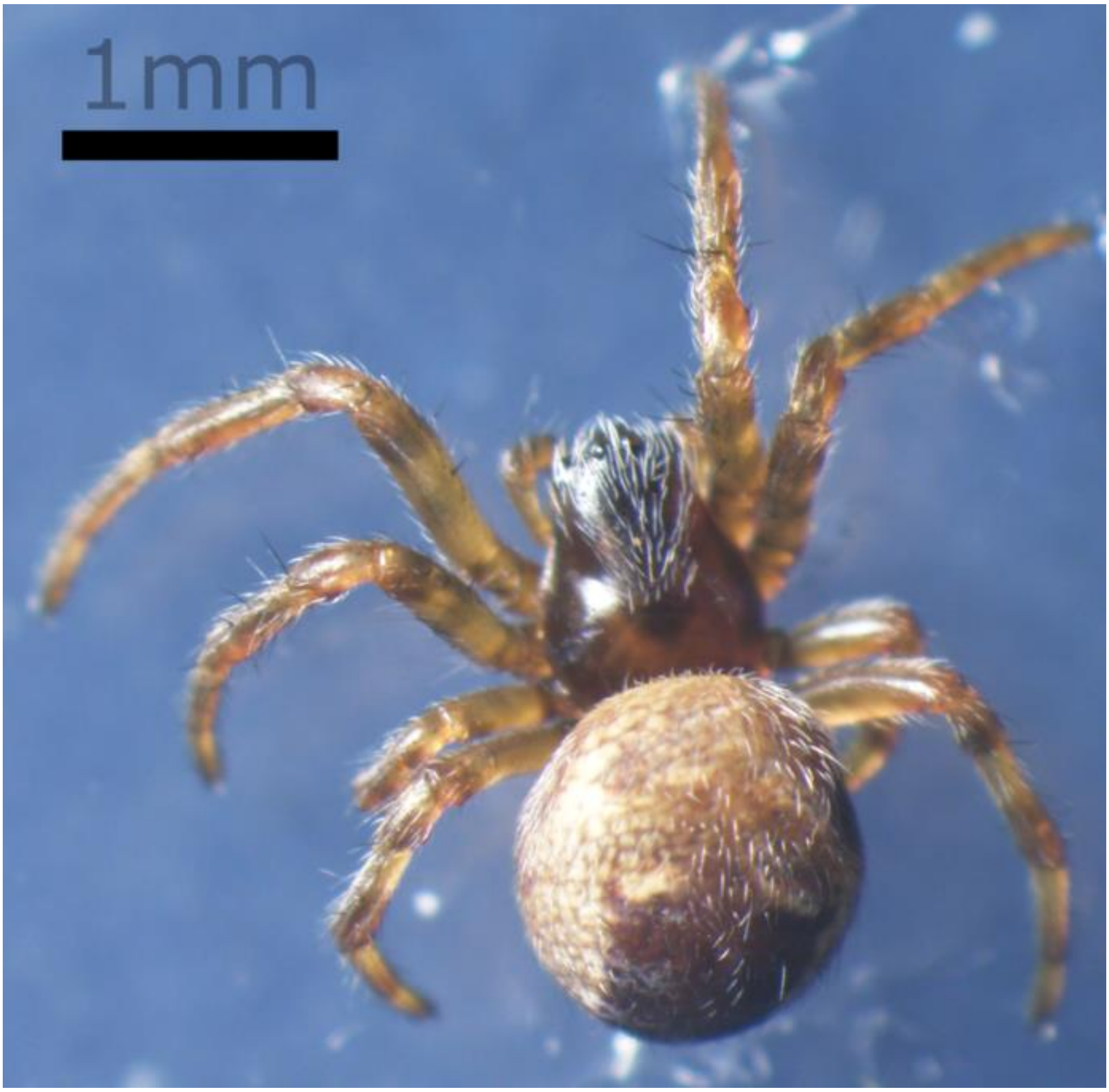

Dictyna major

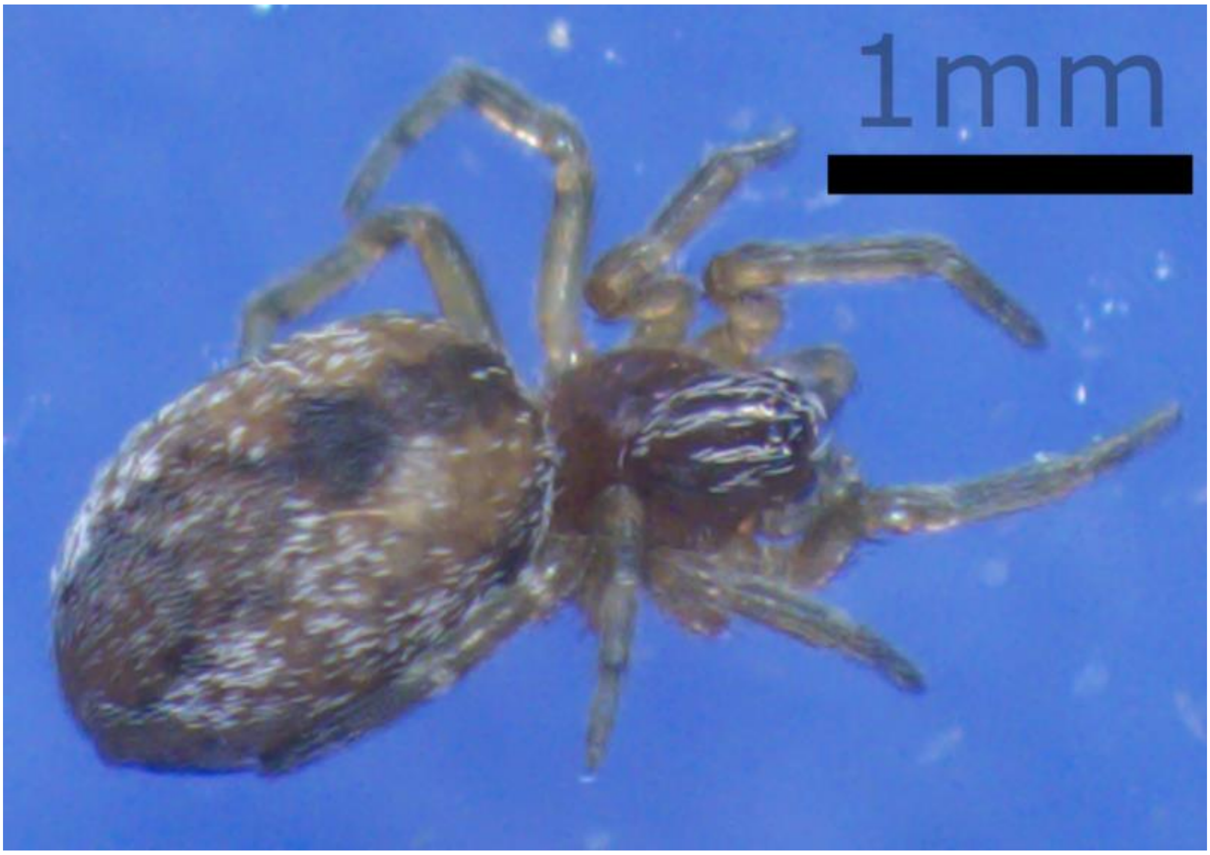

Emblyna borealis

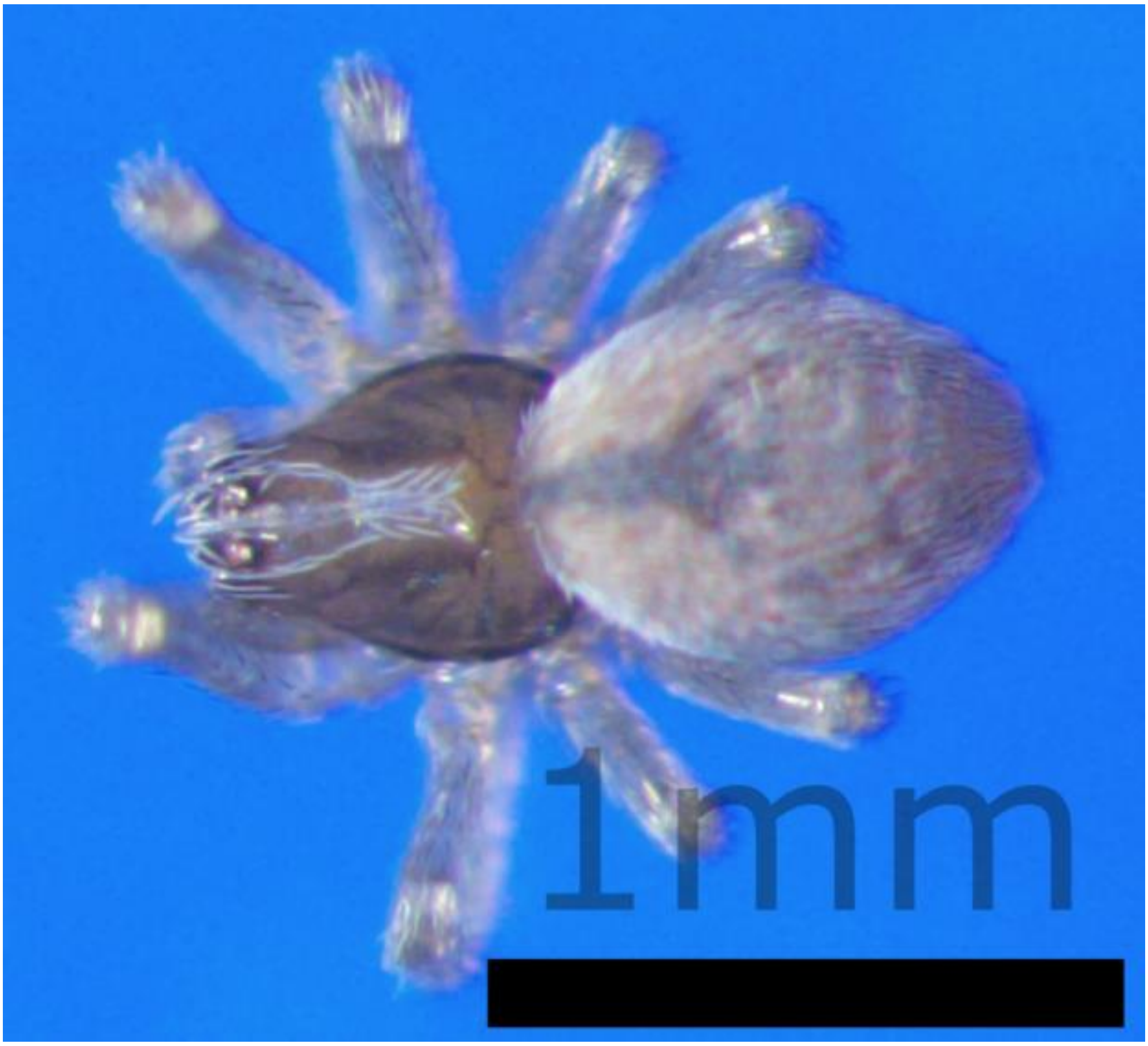

Haplodrassus signifier

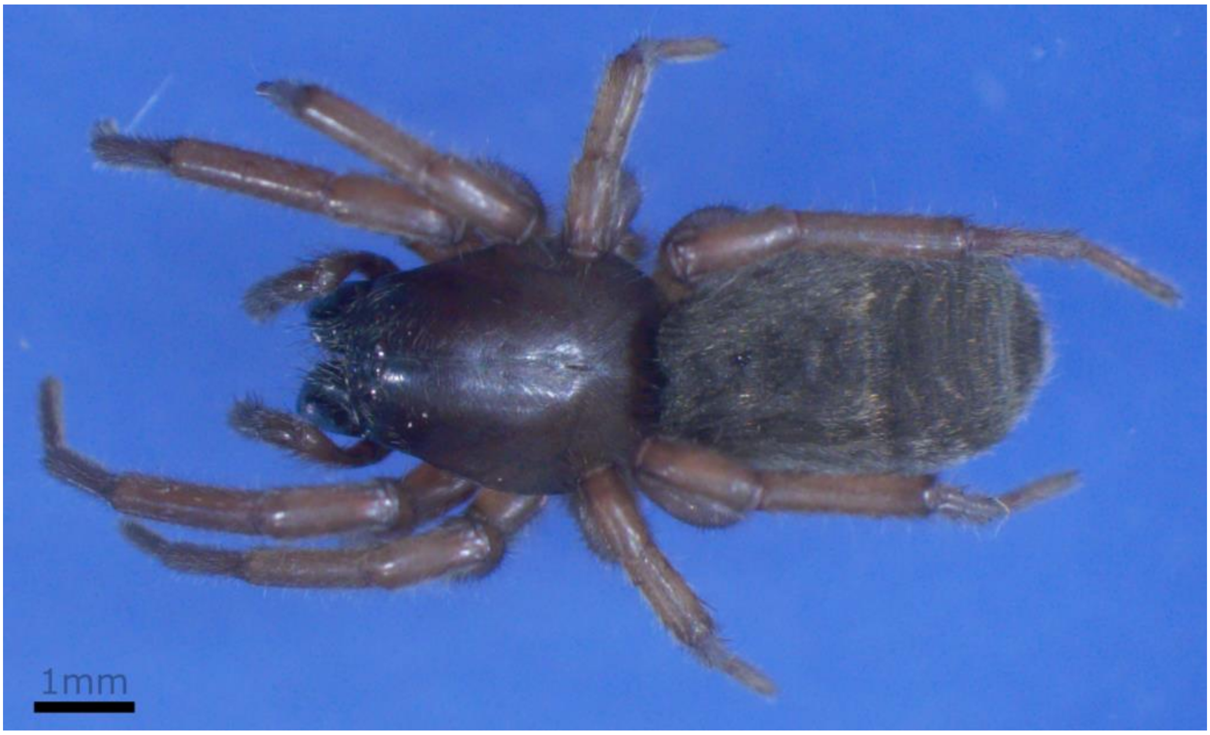

Ohlertidion sp.

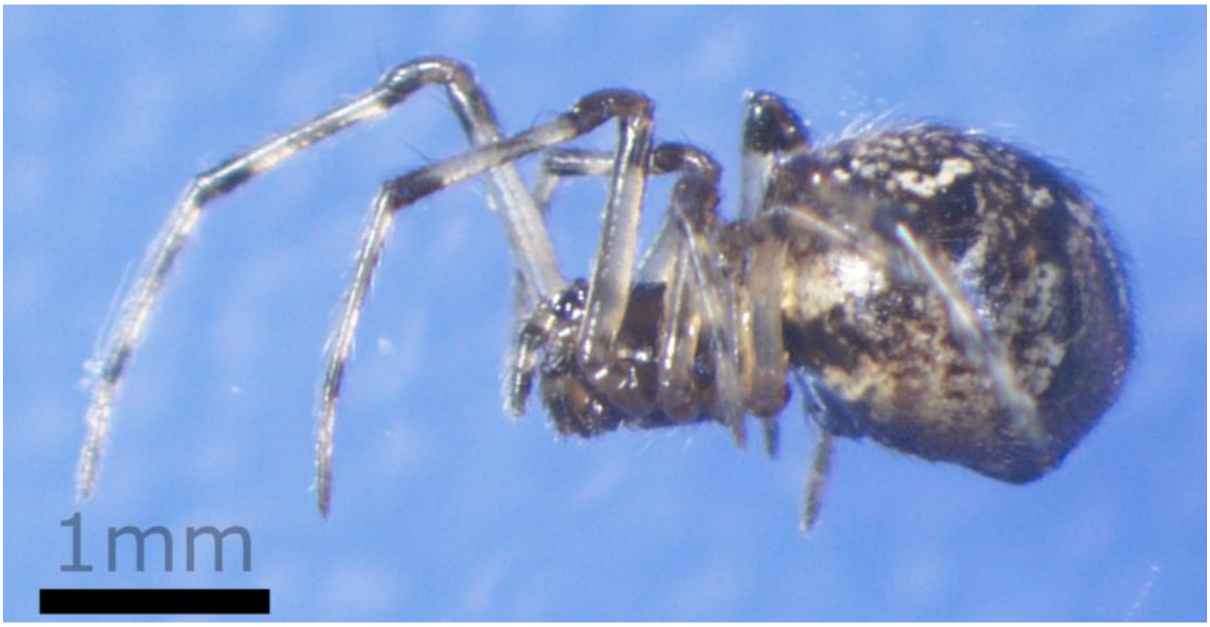

Pardosa furcifera

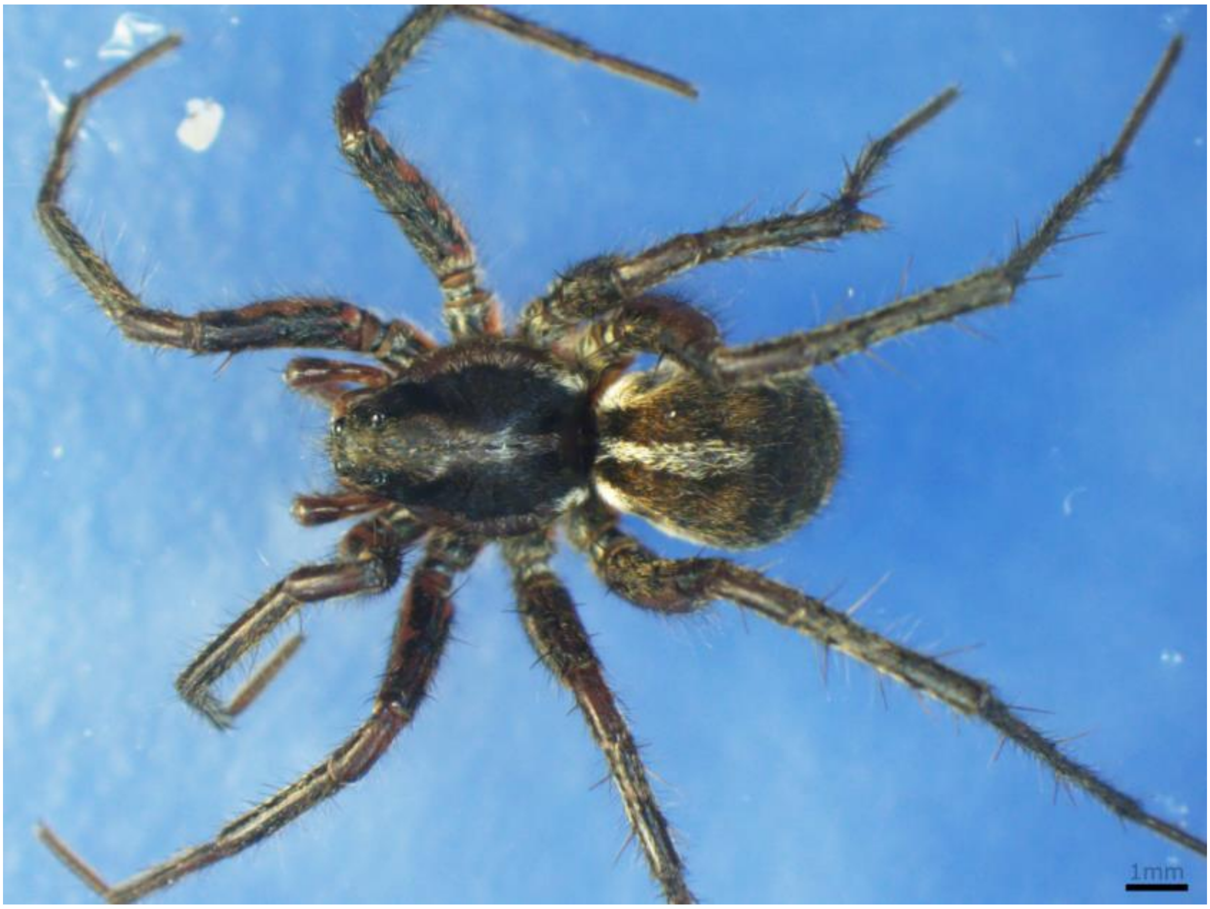

Pardosa groenlandica

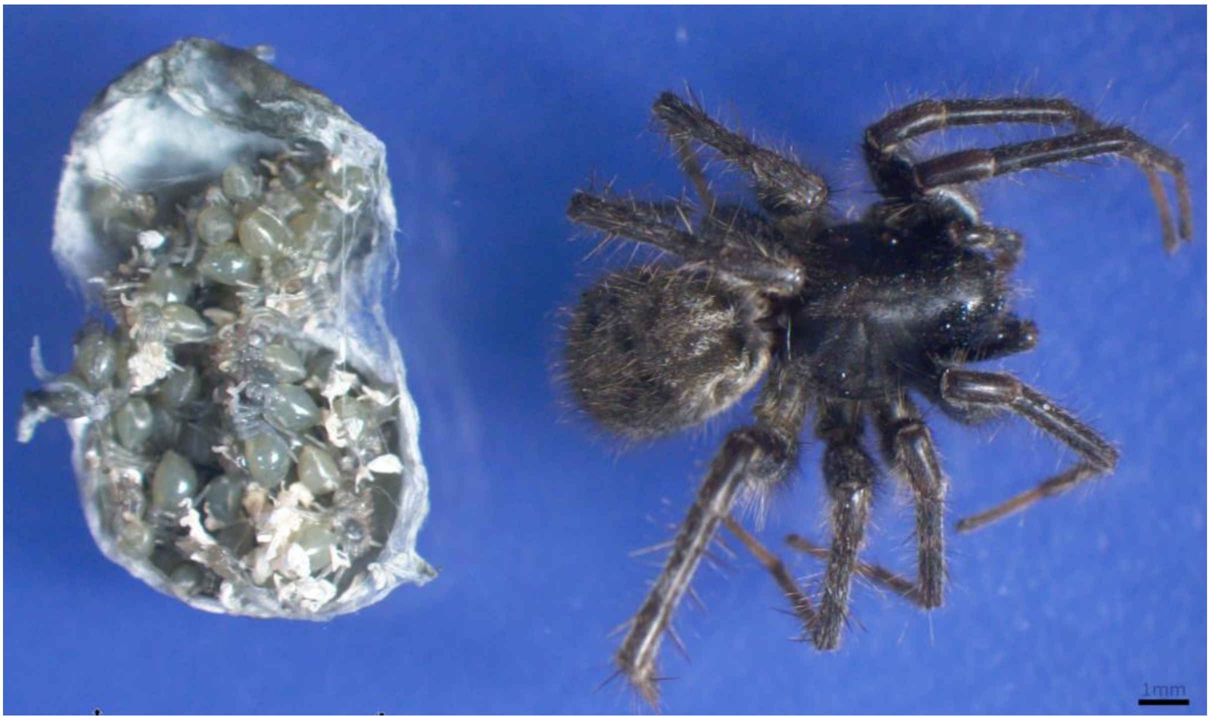

Tetragnatha extensa

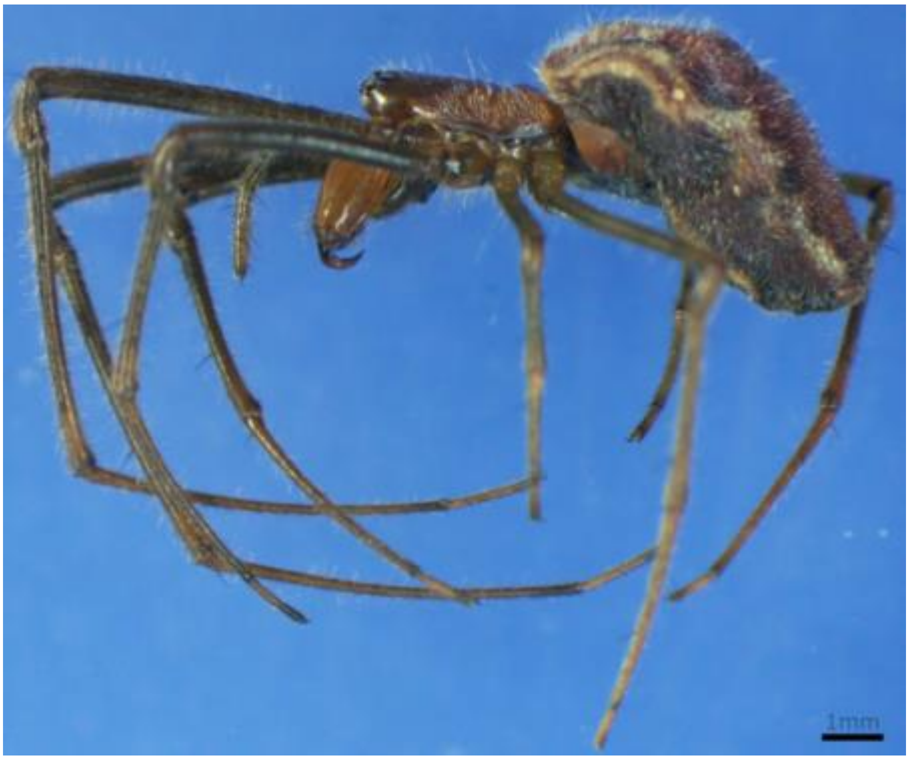

Theridion ohlerti

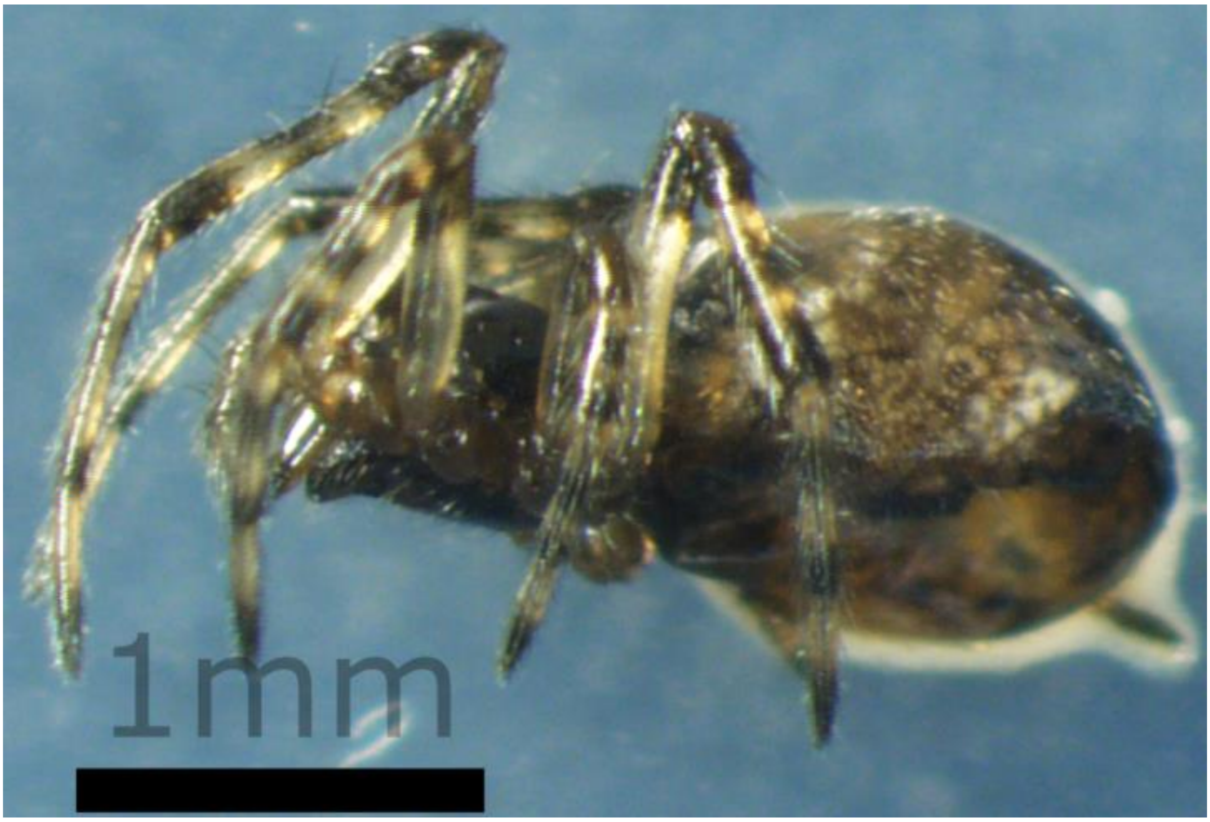

Xysticus sp.

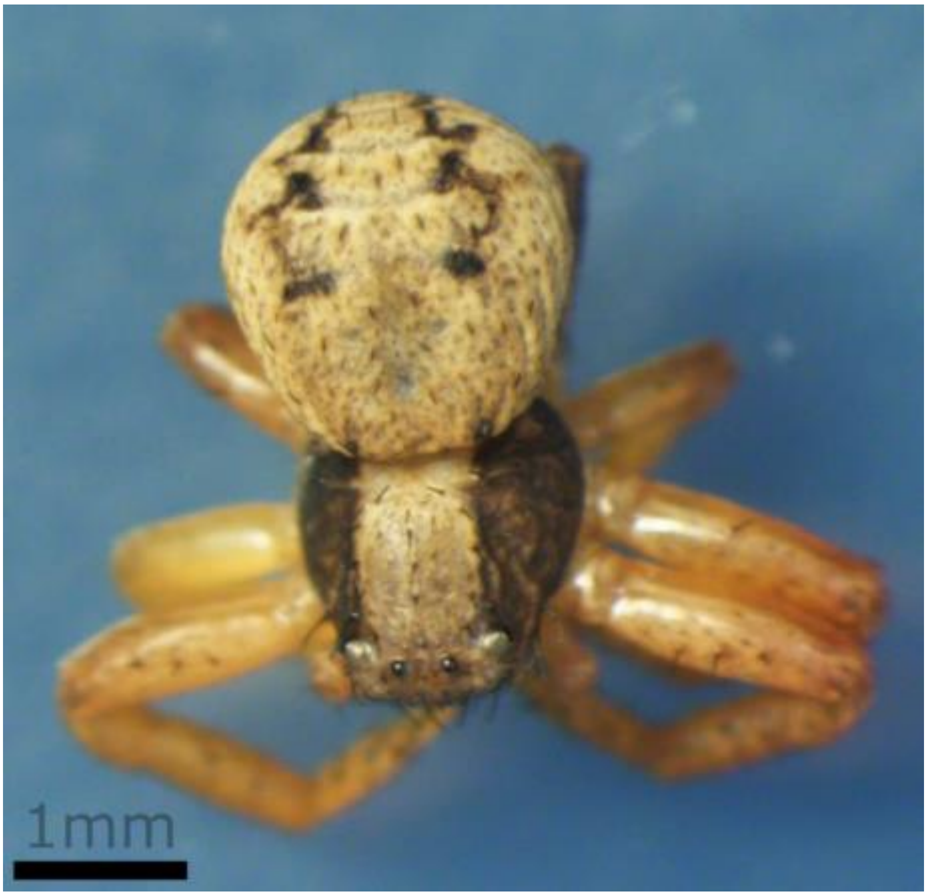

Chilopoda

Lamyctes emarginatus

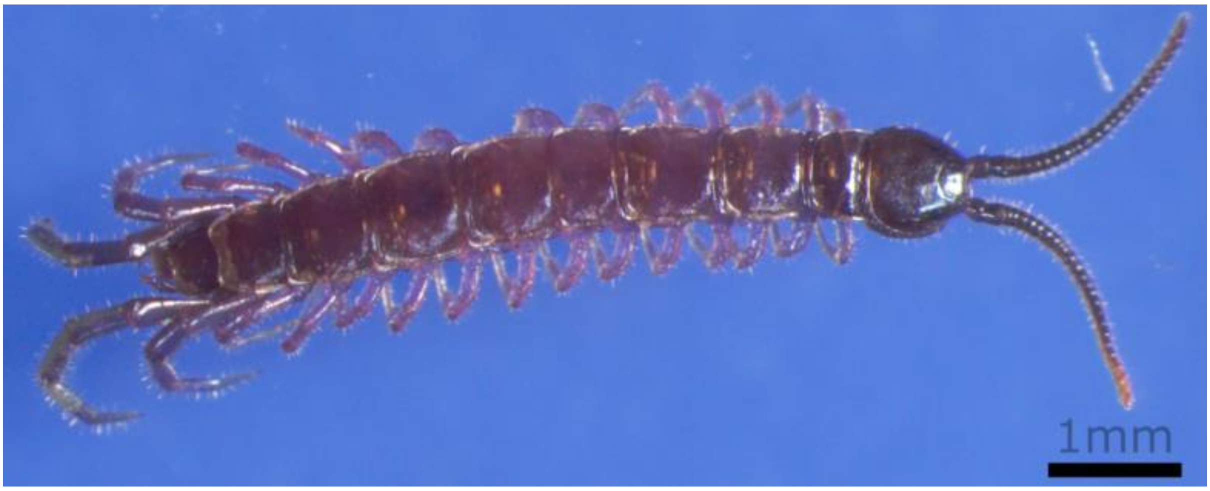

Lamyctes sp.

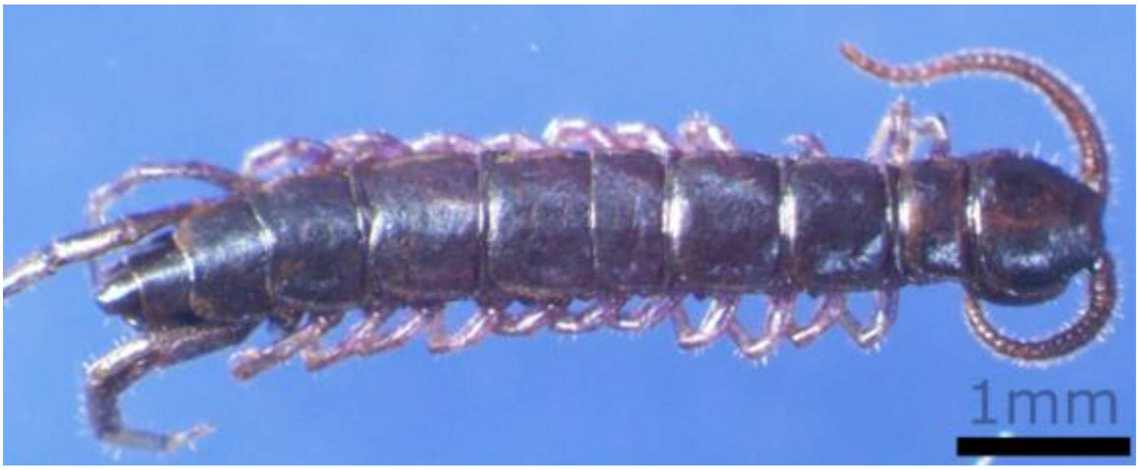

Coleoptera

Bembidion grapii

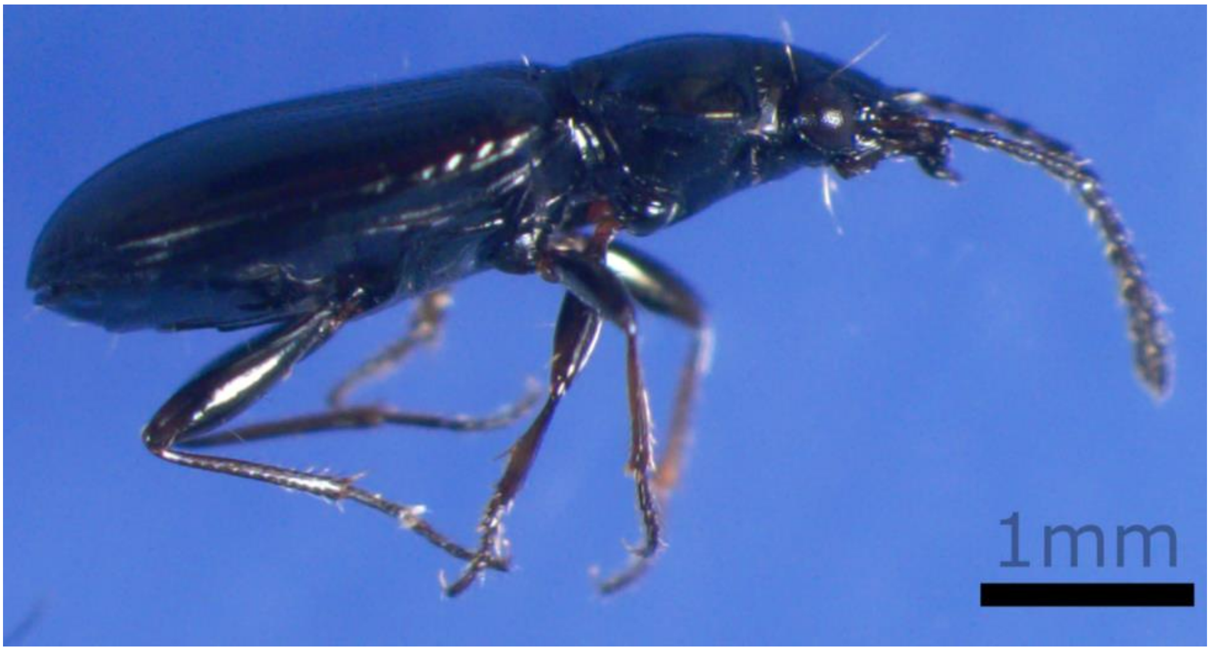

Byrrhus fasciatus

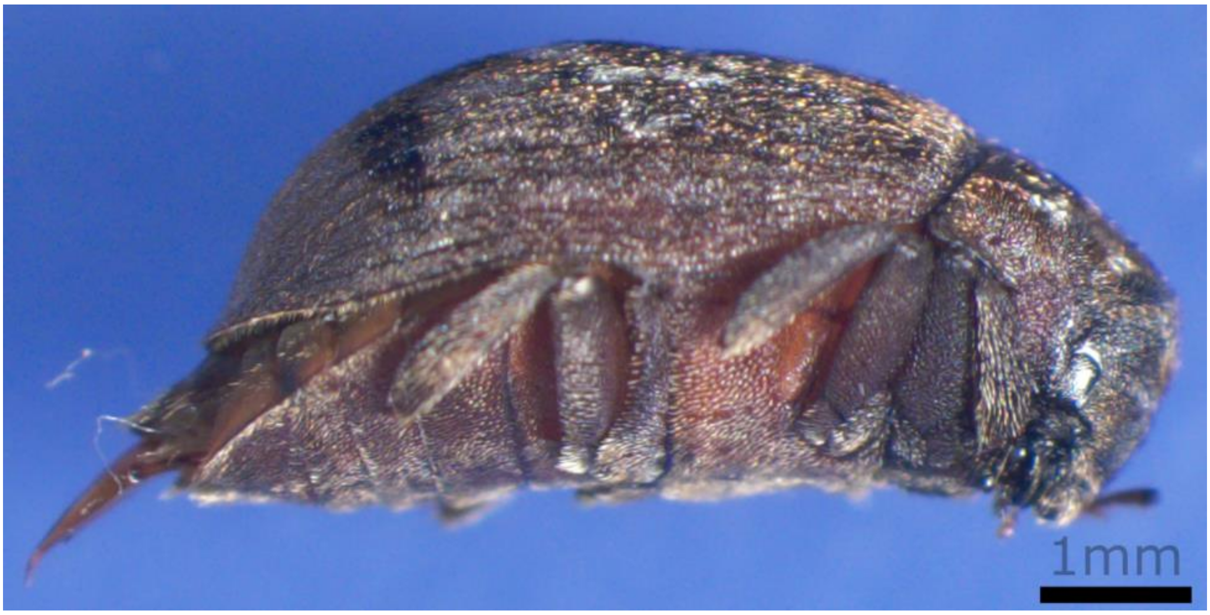

Coccinella transversoguttata

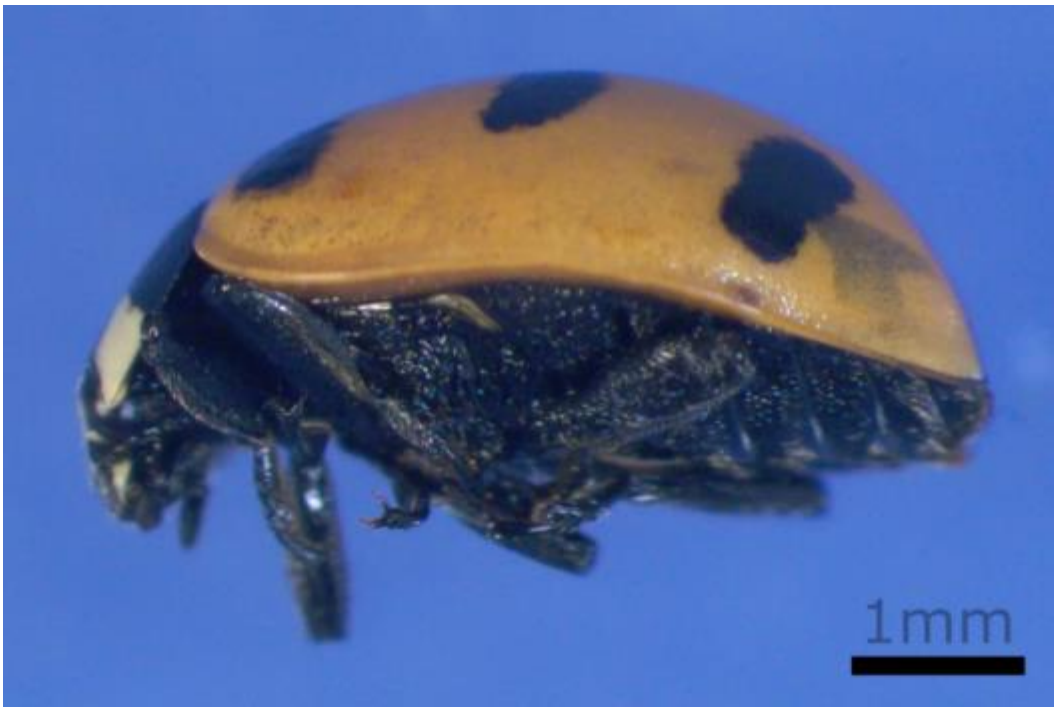

Gyrinus opacus

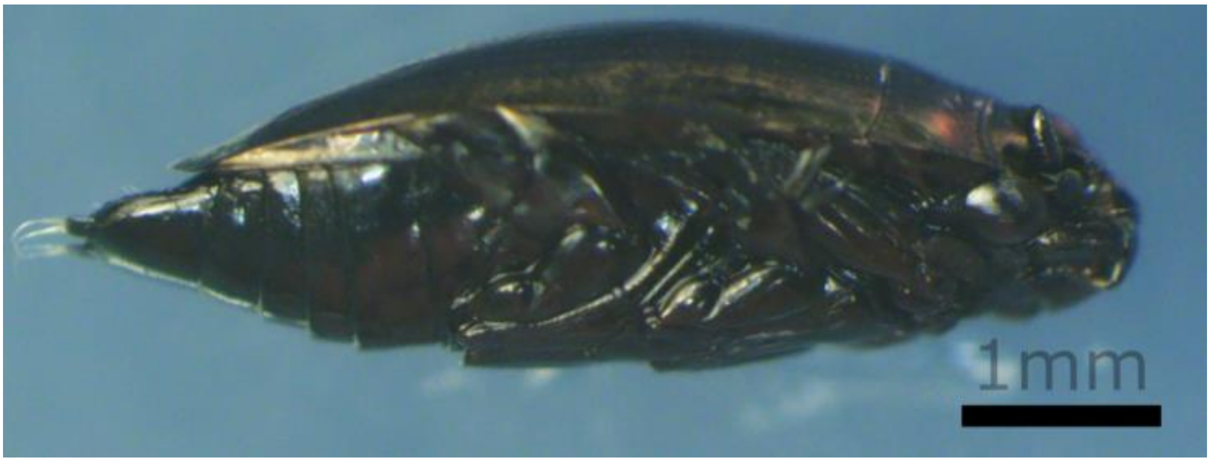

Hydrophorus morio

No image was captured.

Nebria rufescens

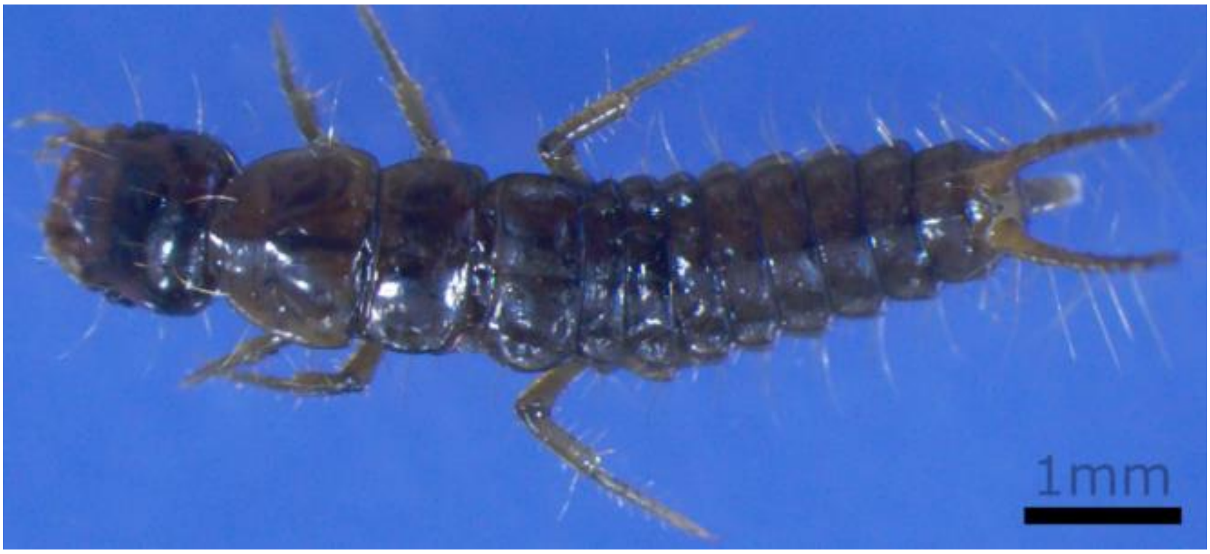

Otiorhynchus arcticus

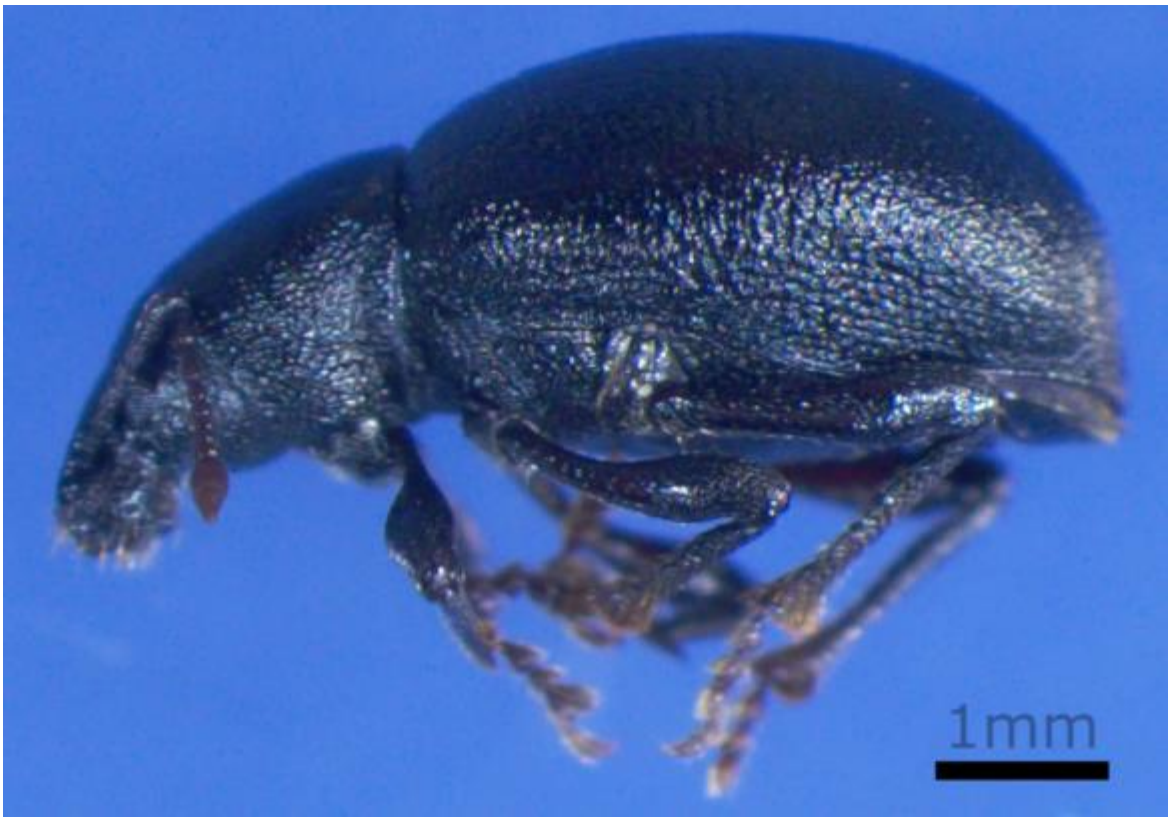

Otiorhynchus nodosus

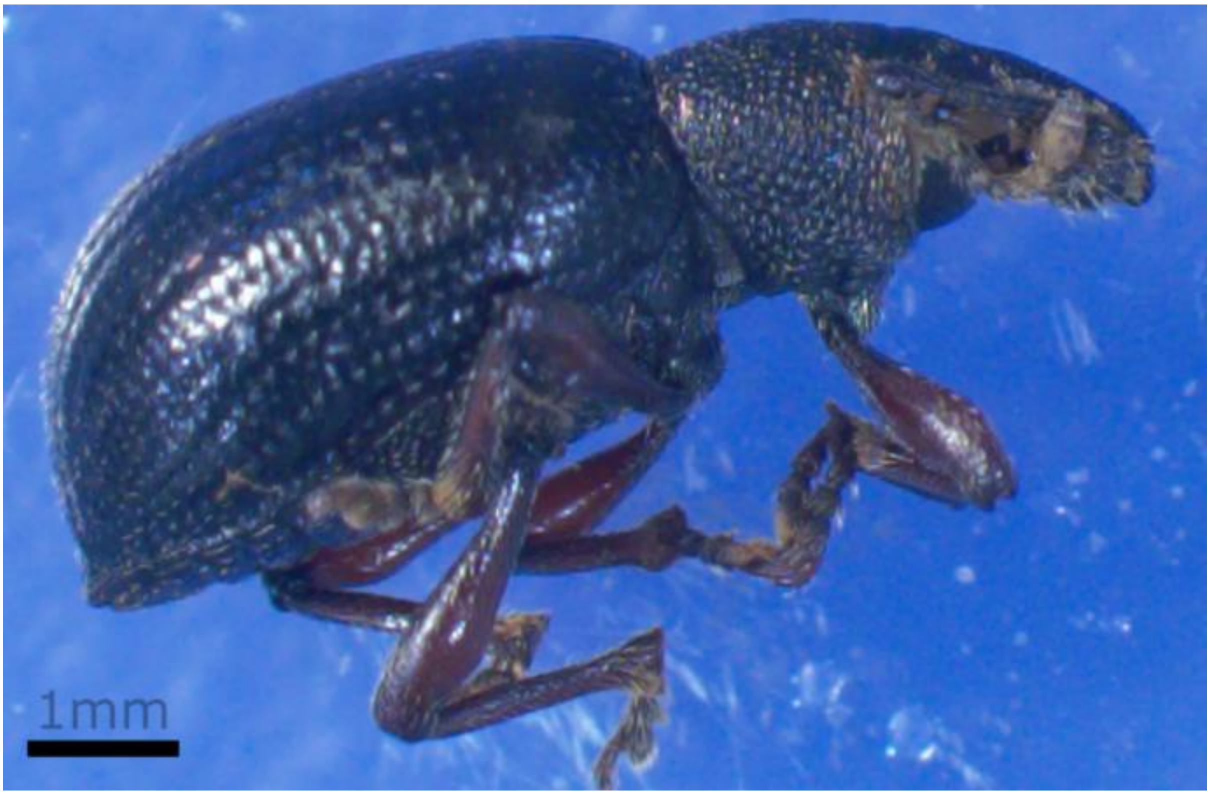

Patrobus septentrionis

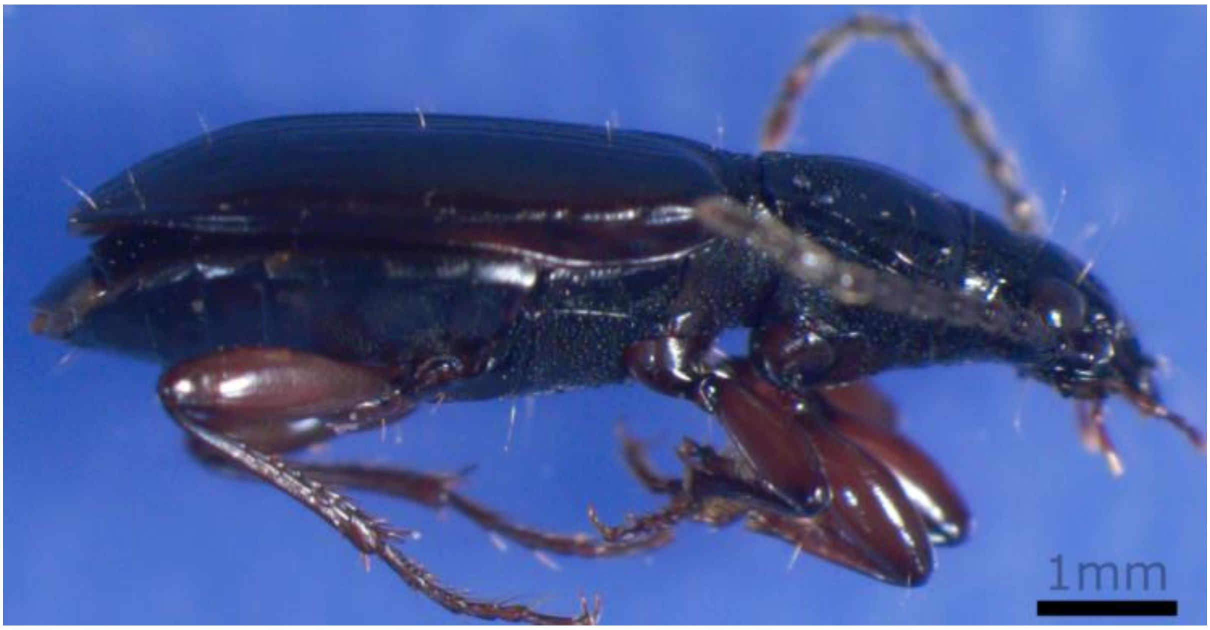

Diptera

Botanophila profuga

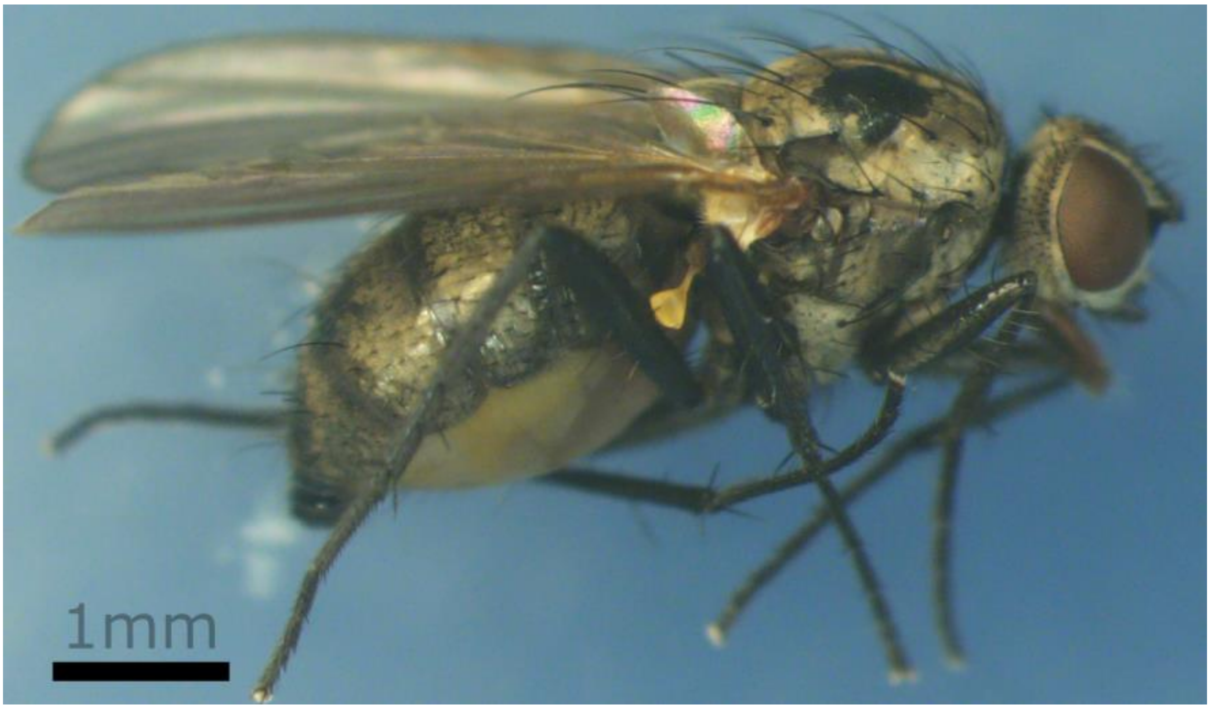

Botanophila sp.

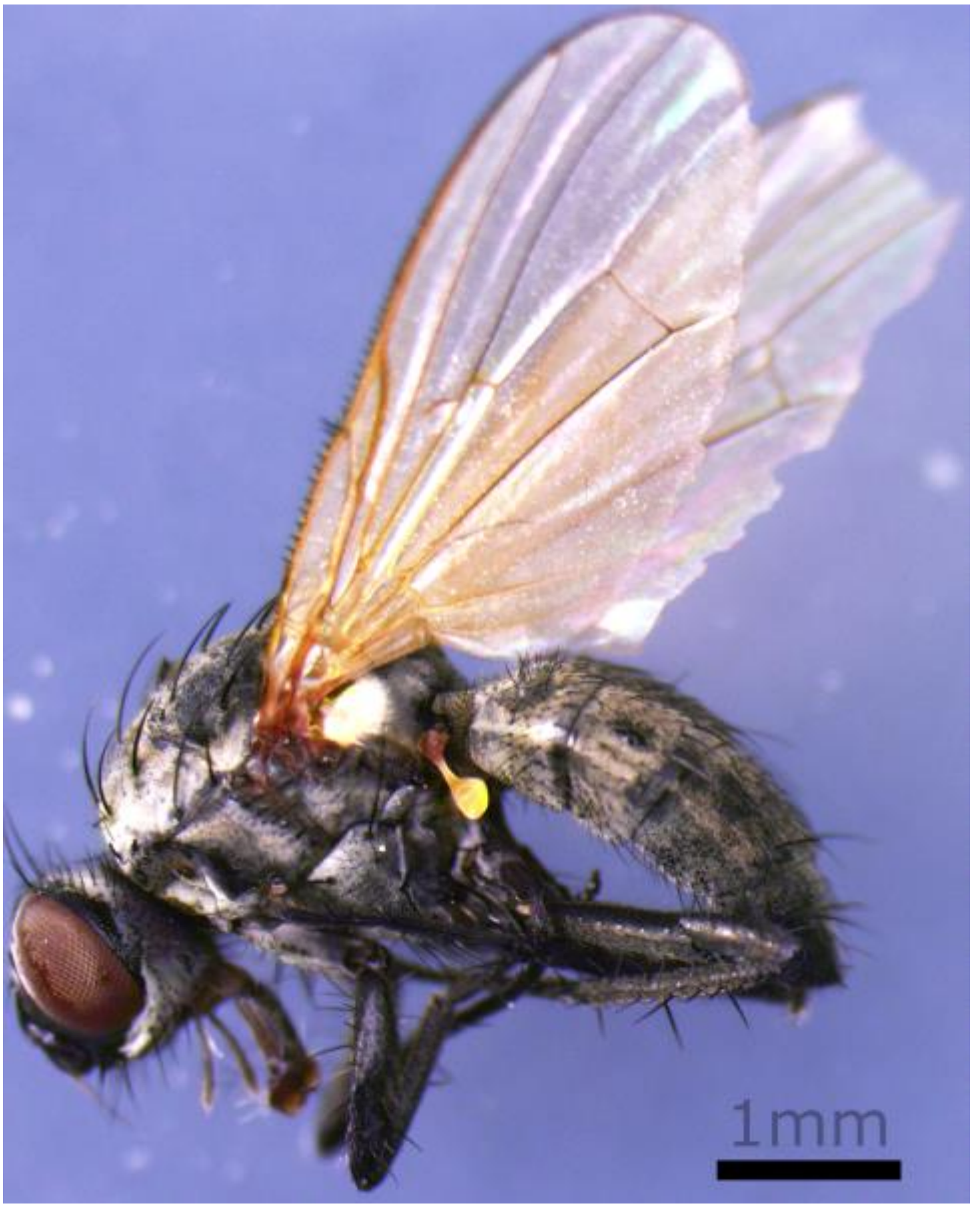

Bradysia sp.

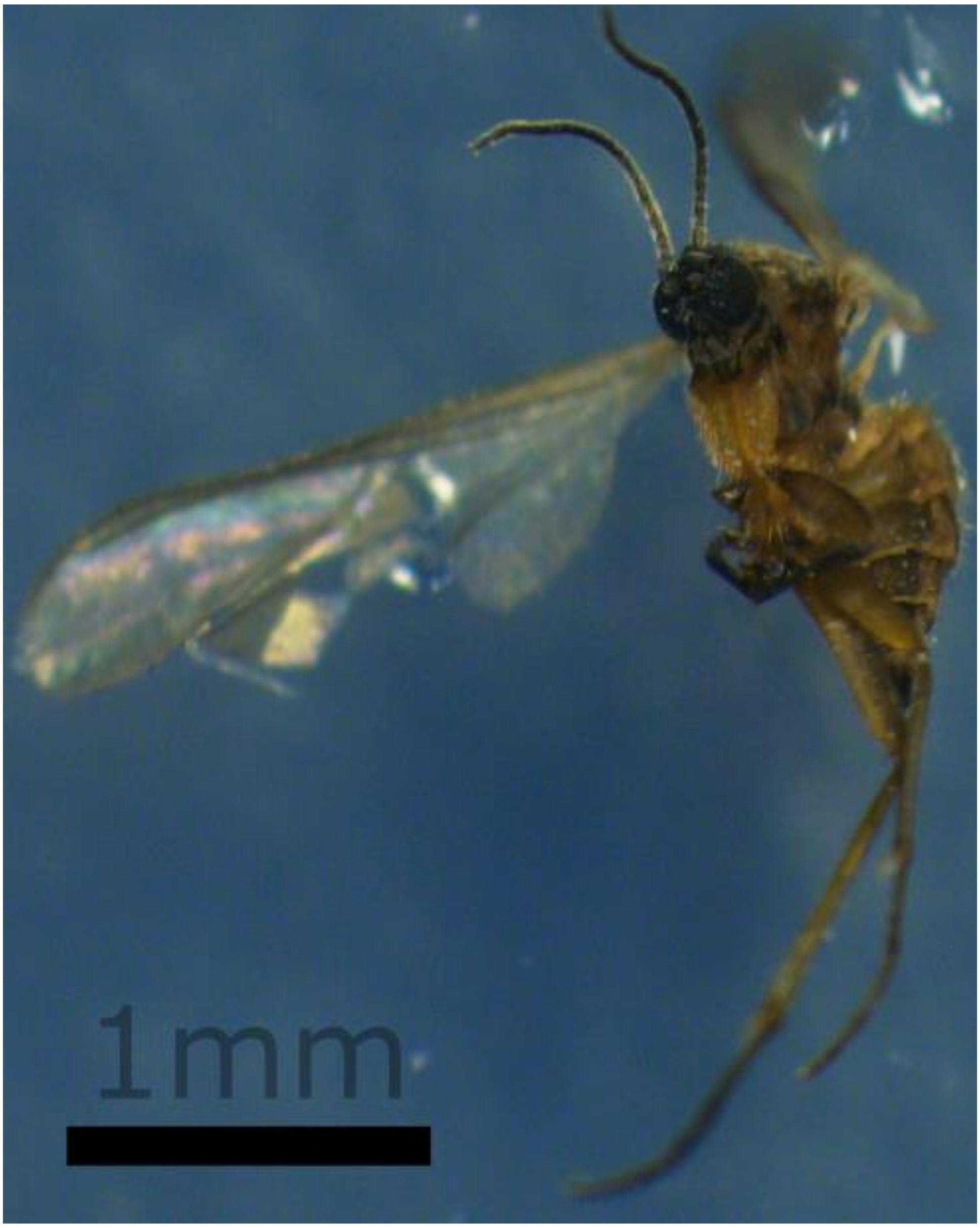

Calliphora uralensis

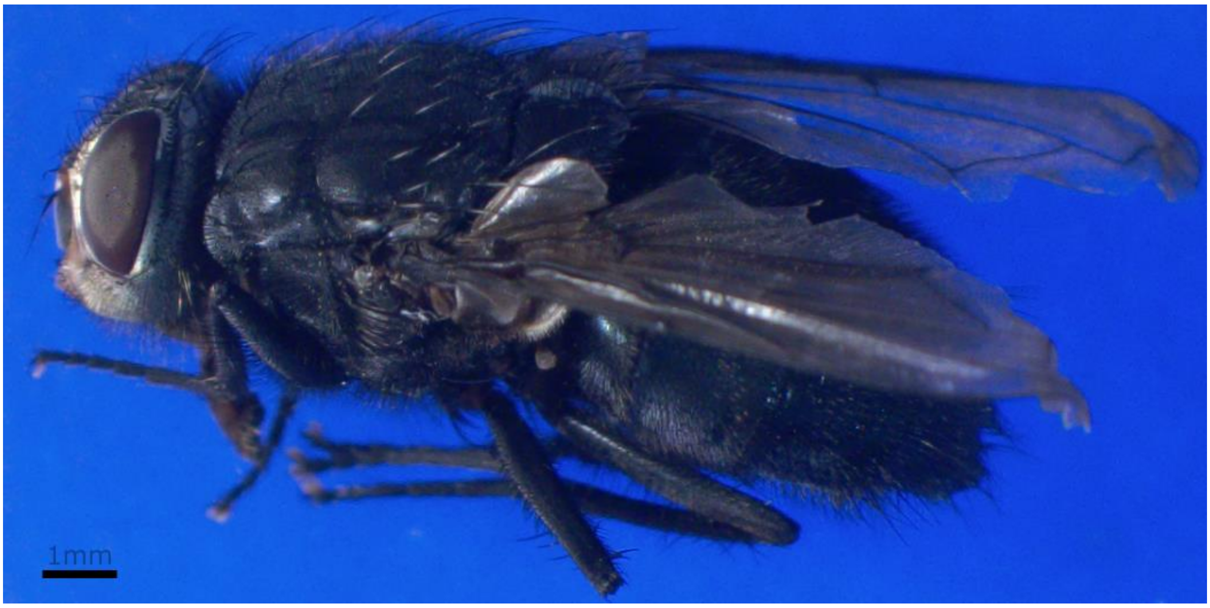

Delia echinata

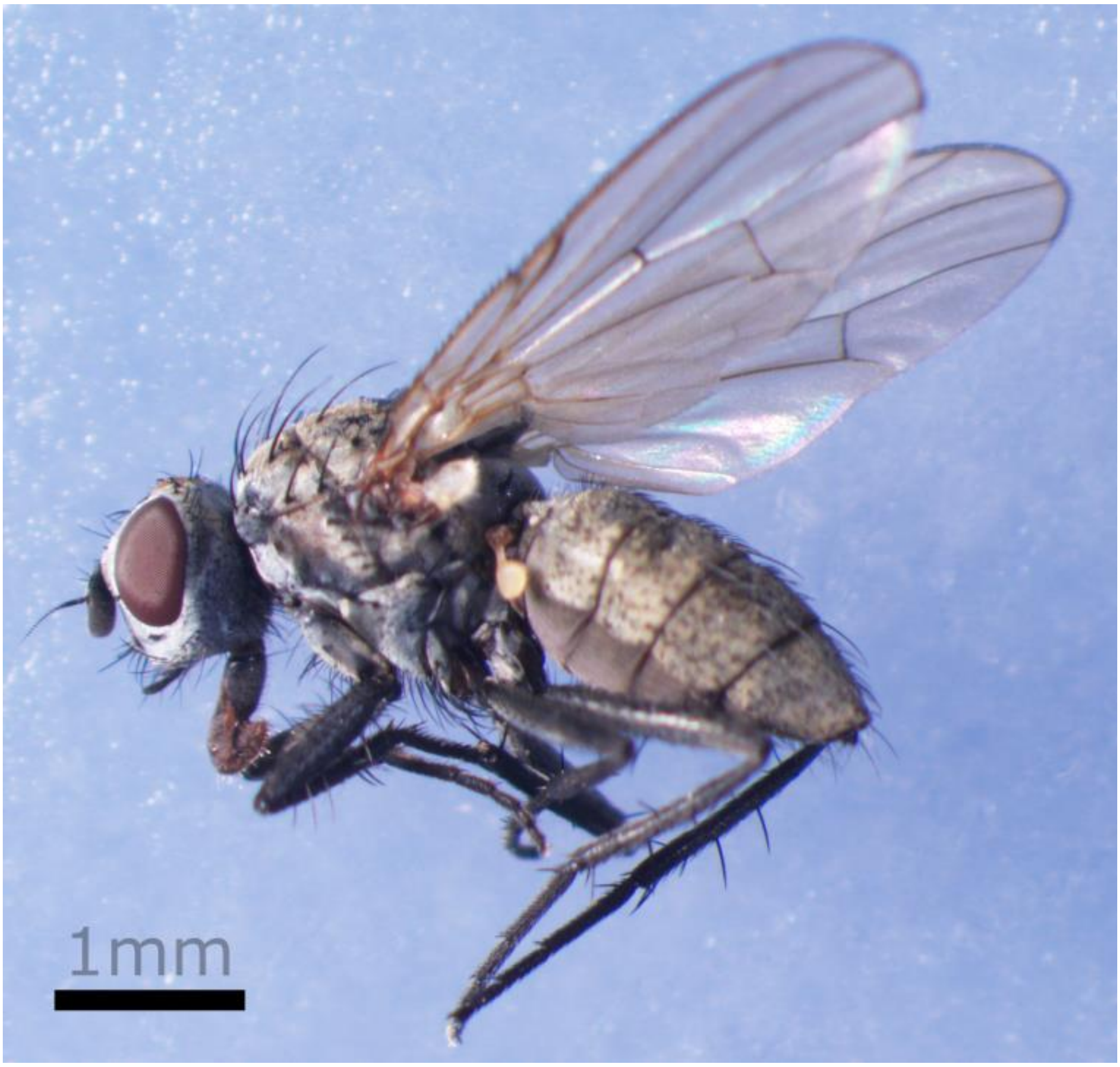

Delia fabricii

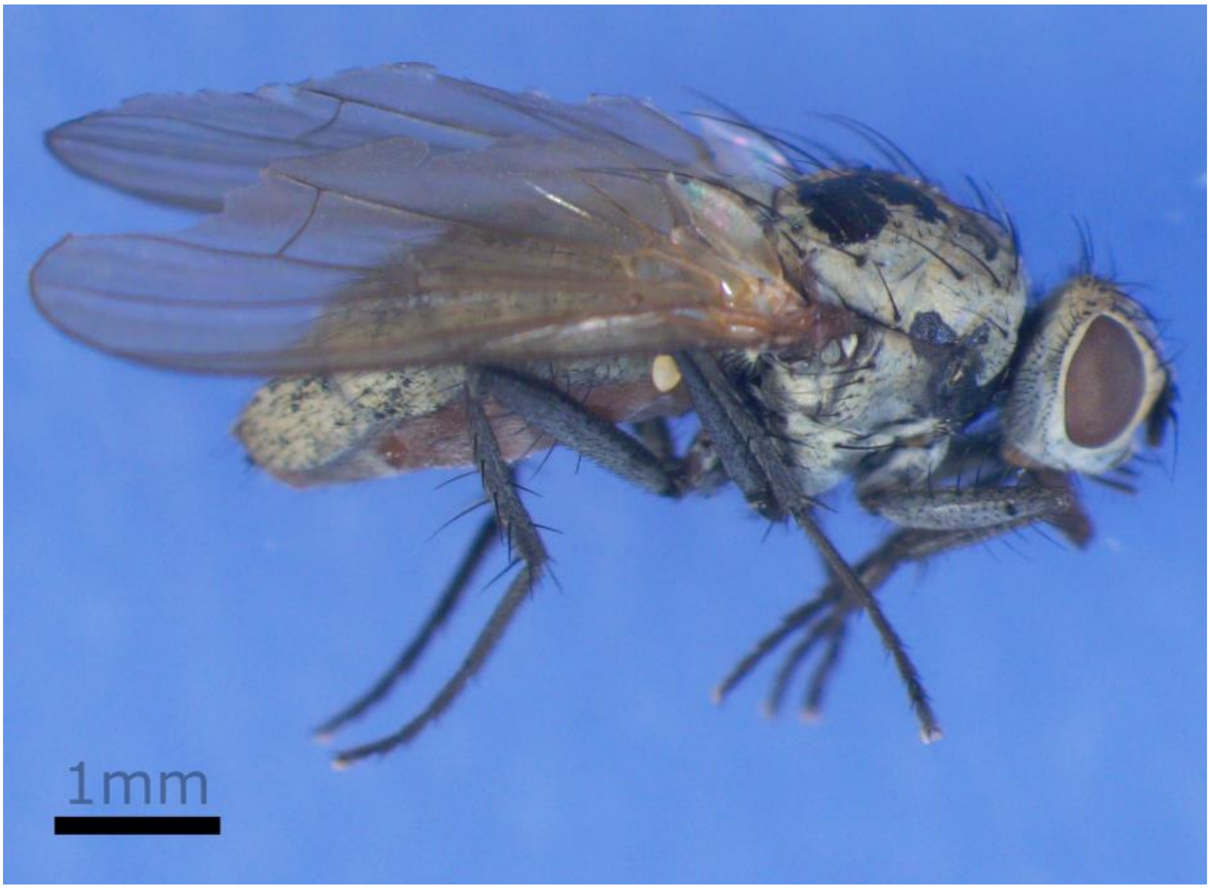

Delia platura

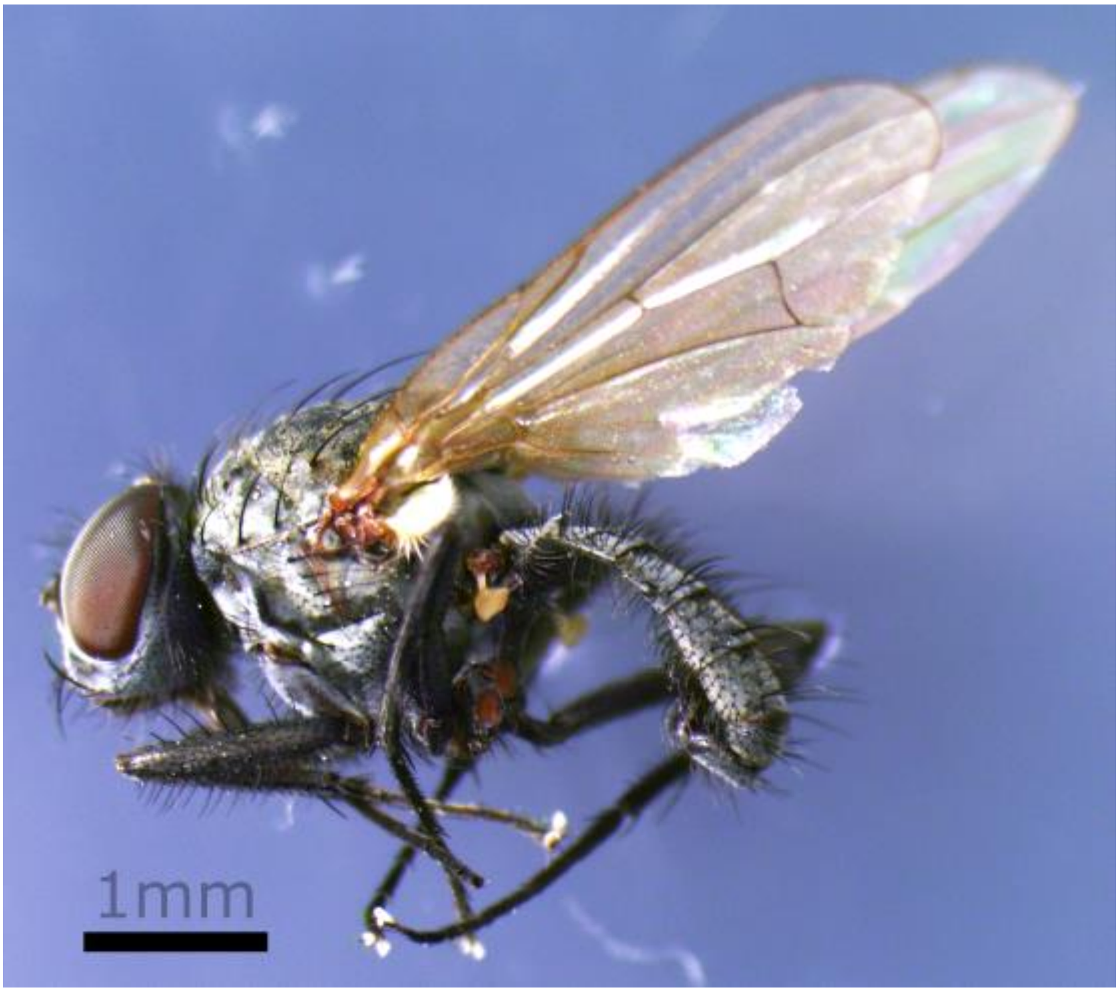

Dolichopus groenlandicus

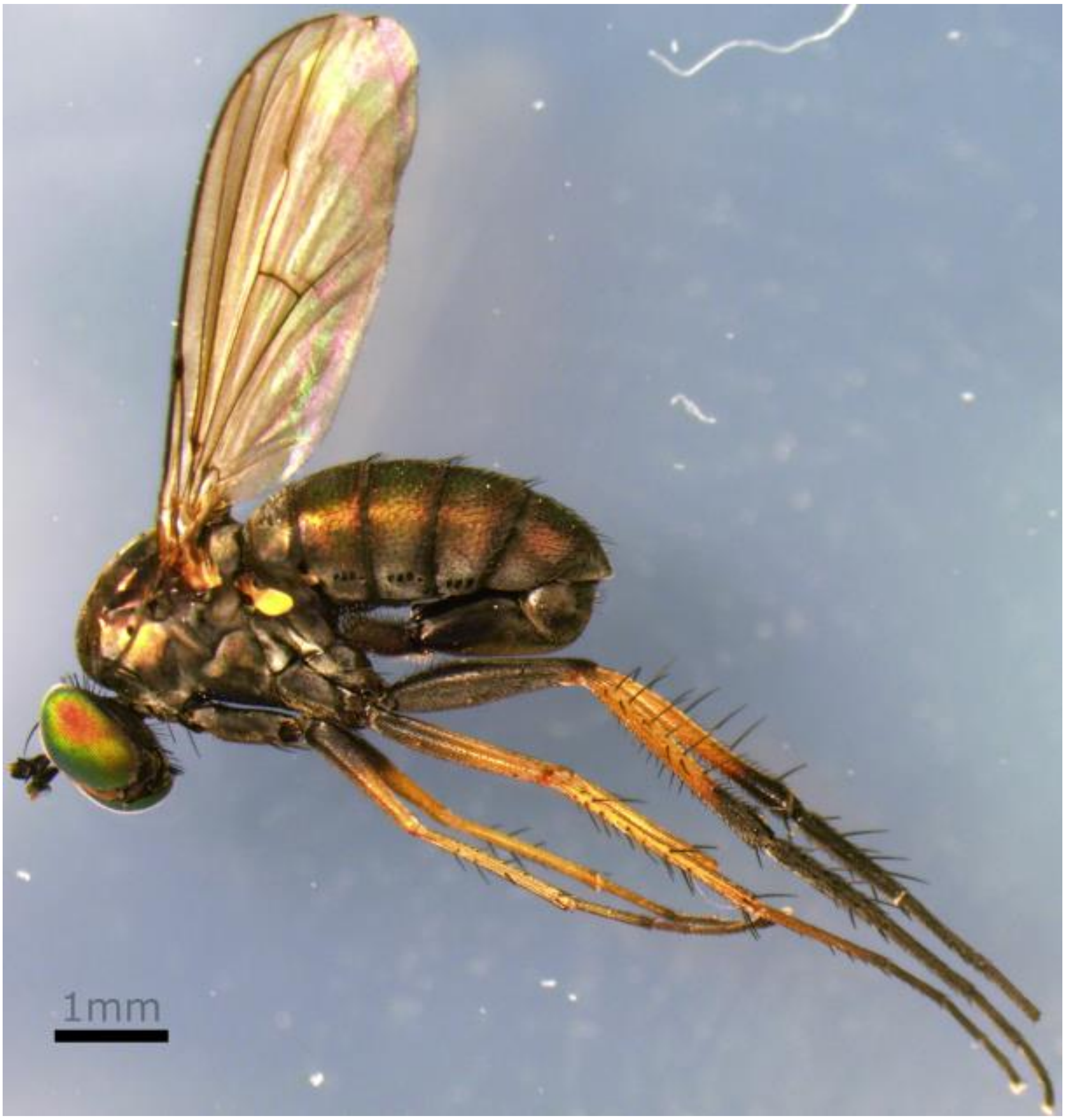

Dolichopus plumipes

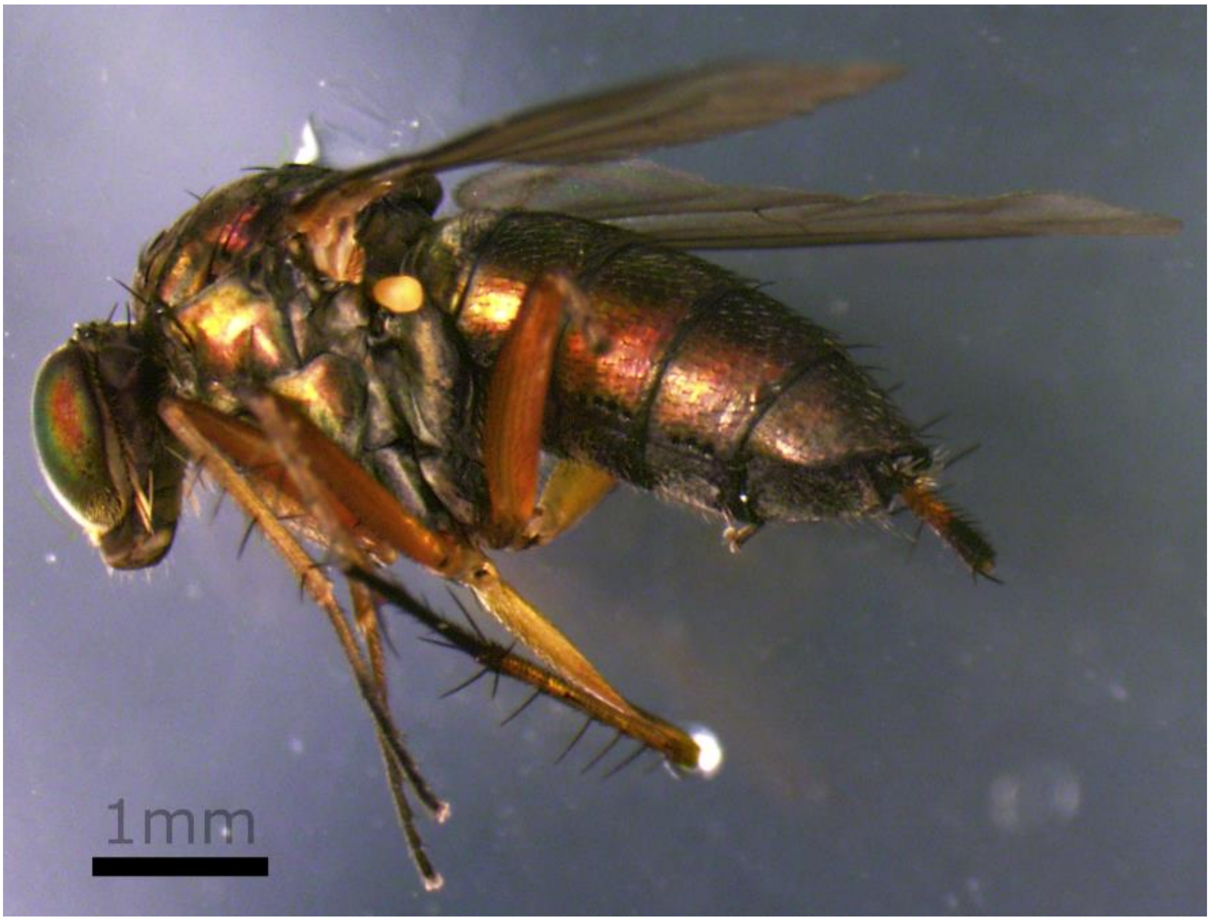

Eupeodes sp.

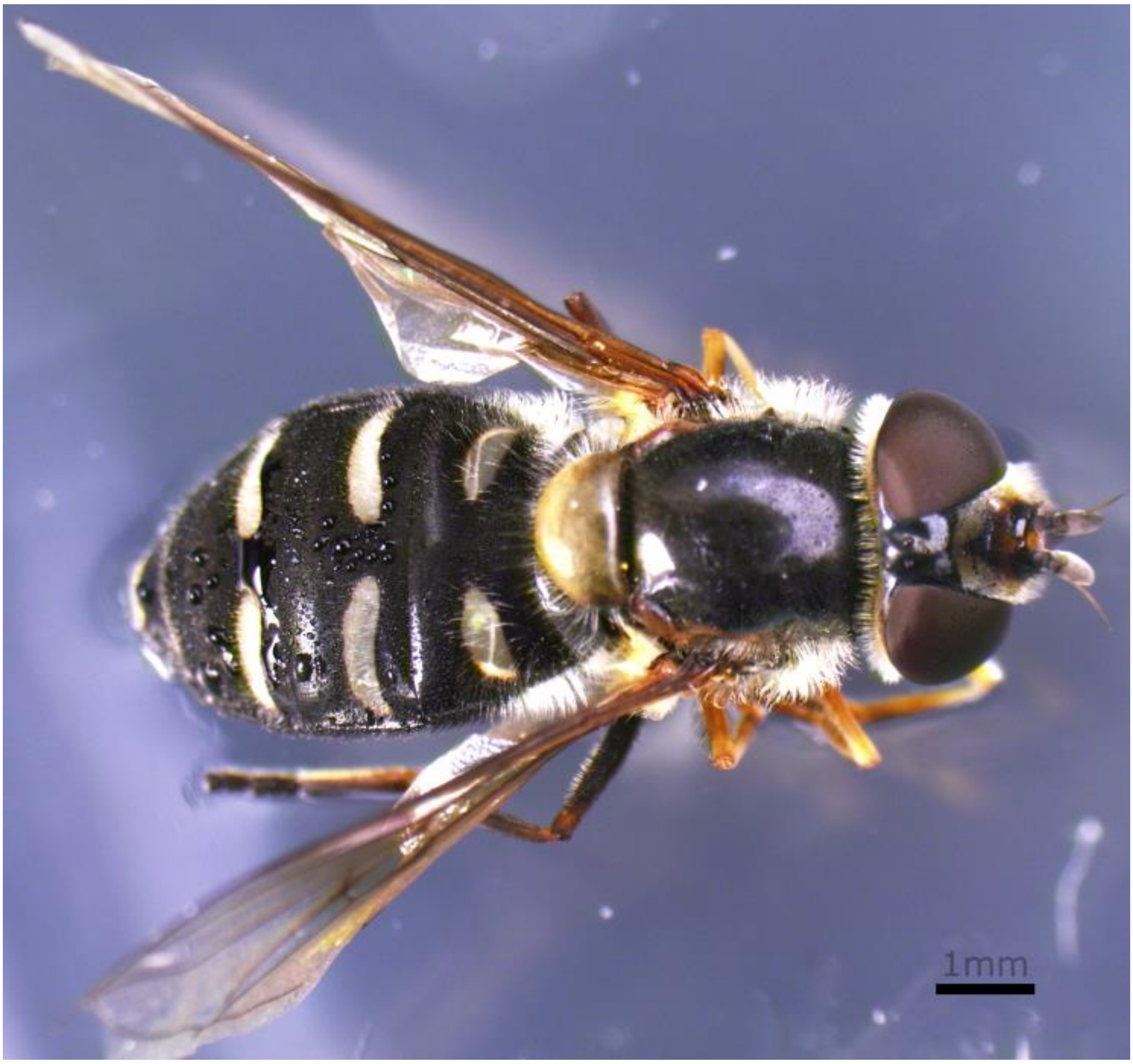

Execchia sp.

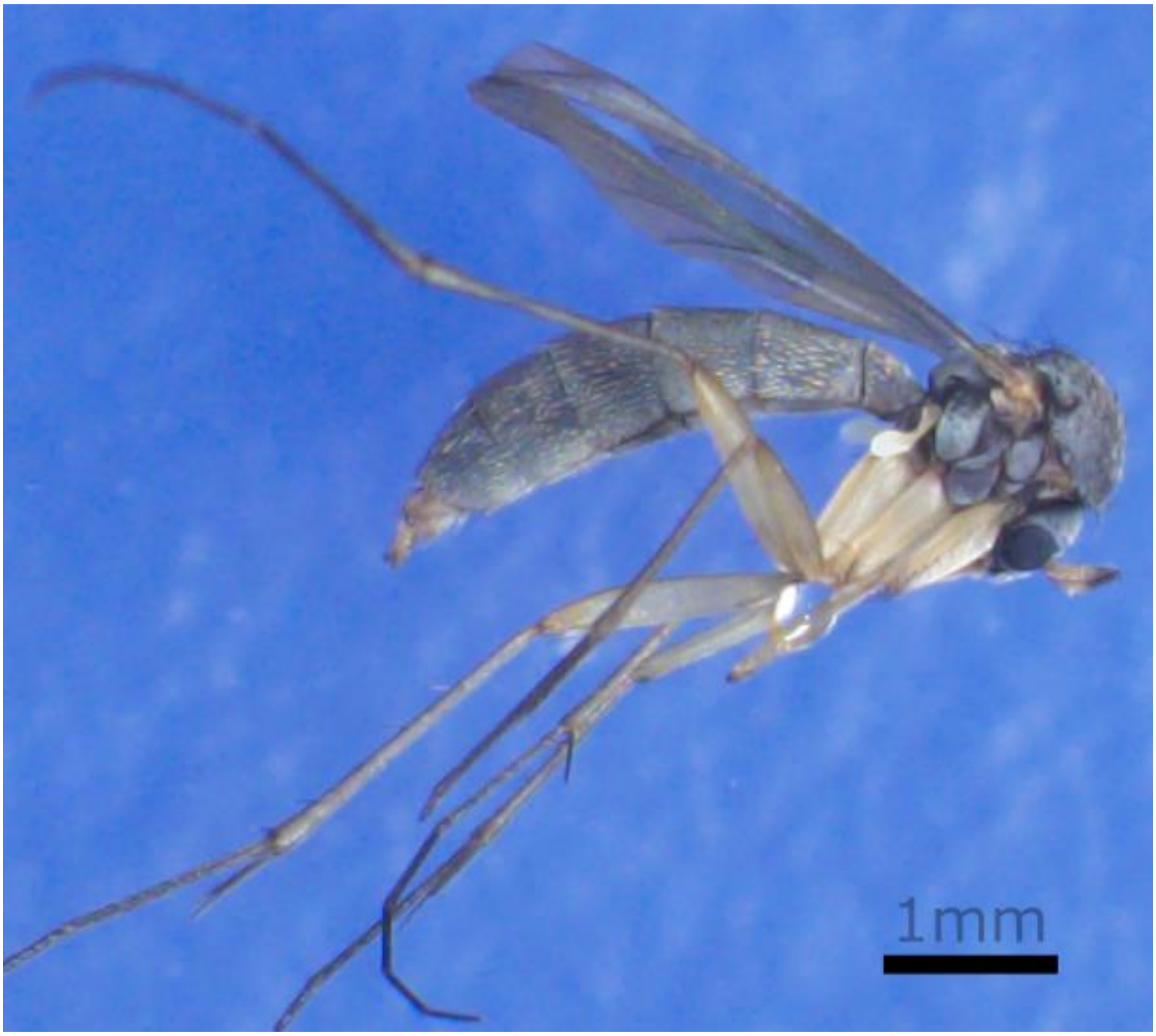

Fannia sp.

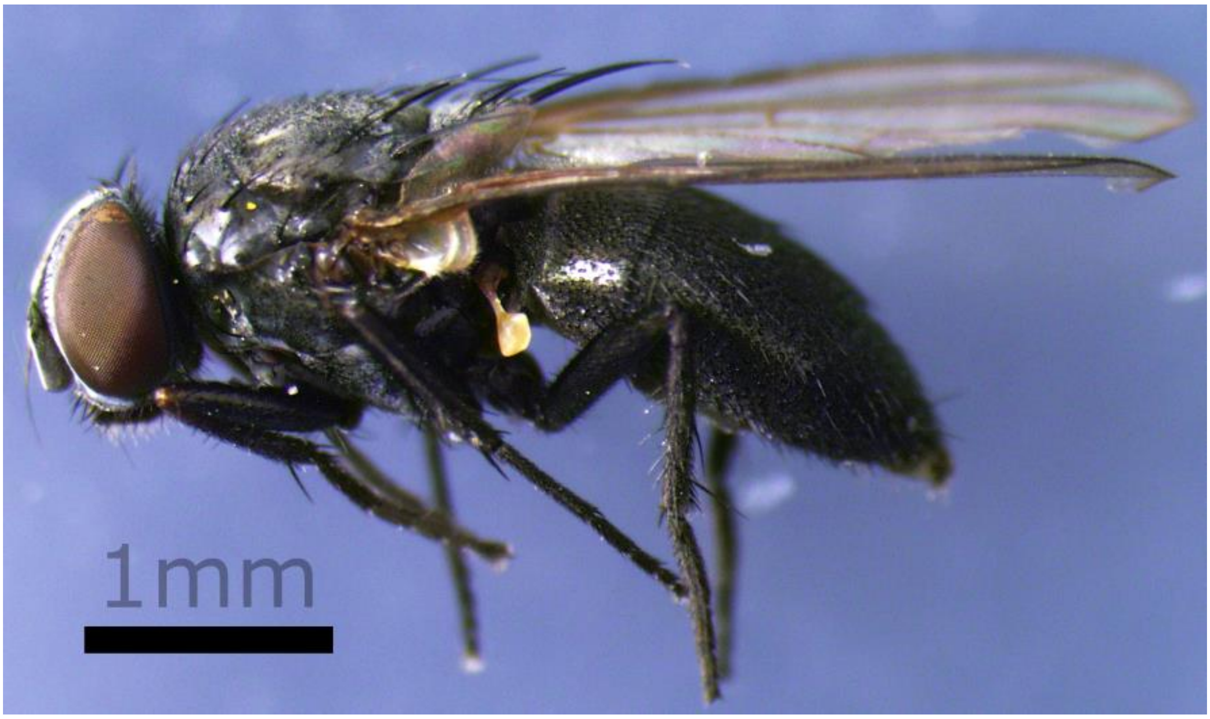

Forcipomyia sp.

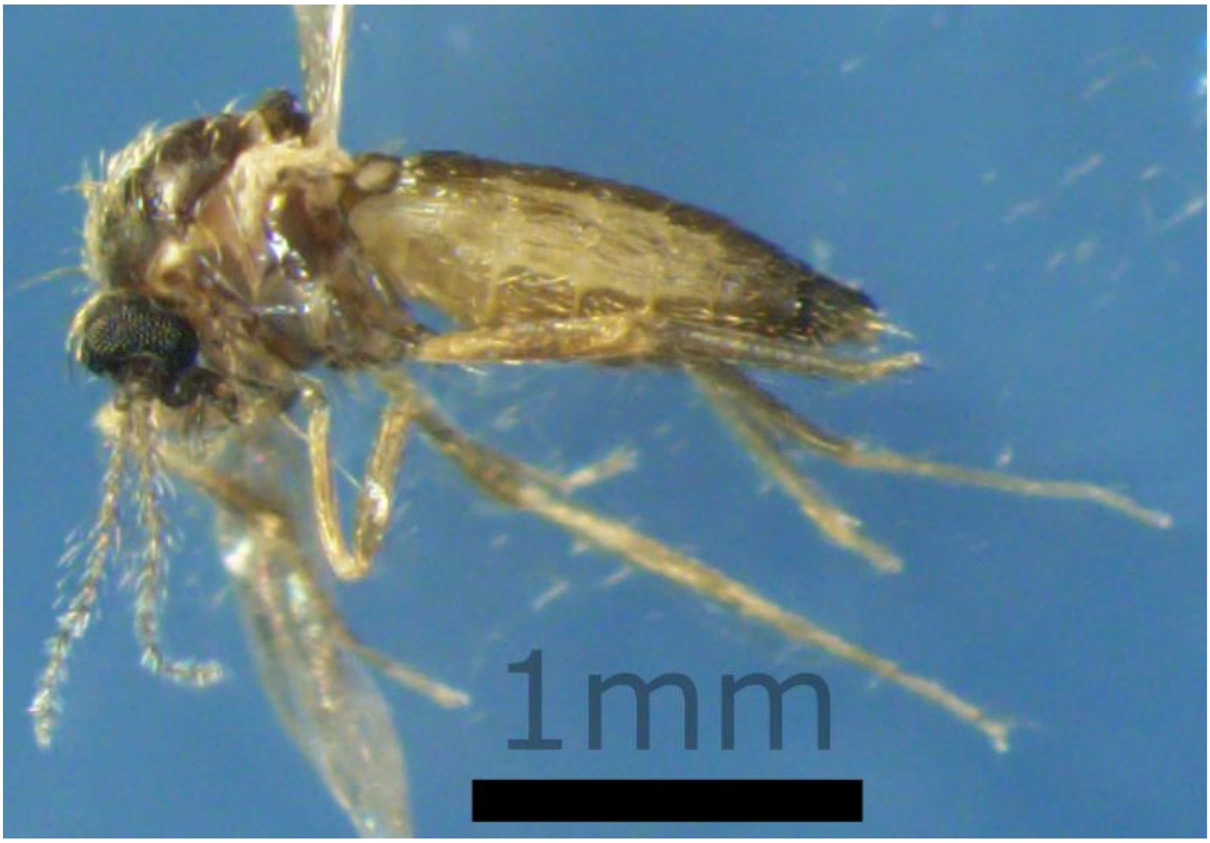

Halocladius variabilis

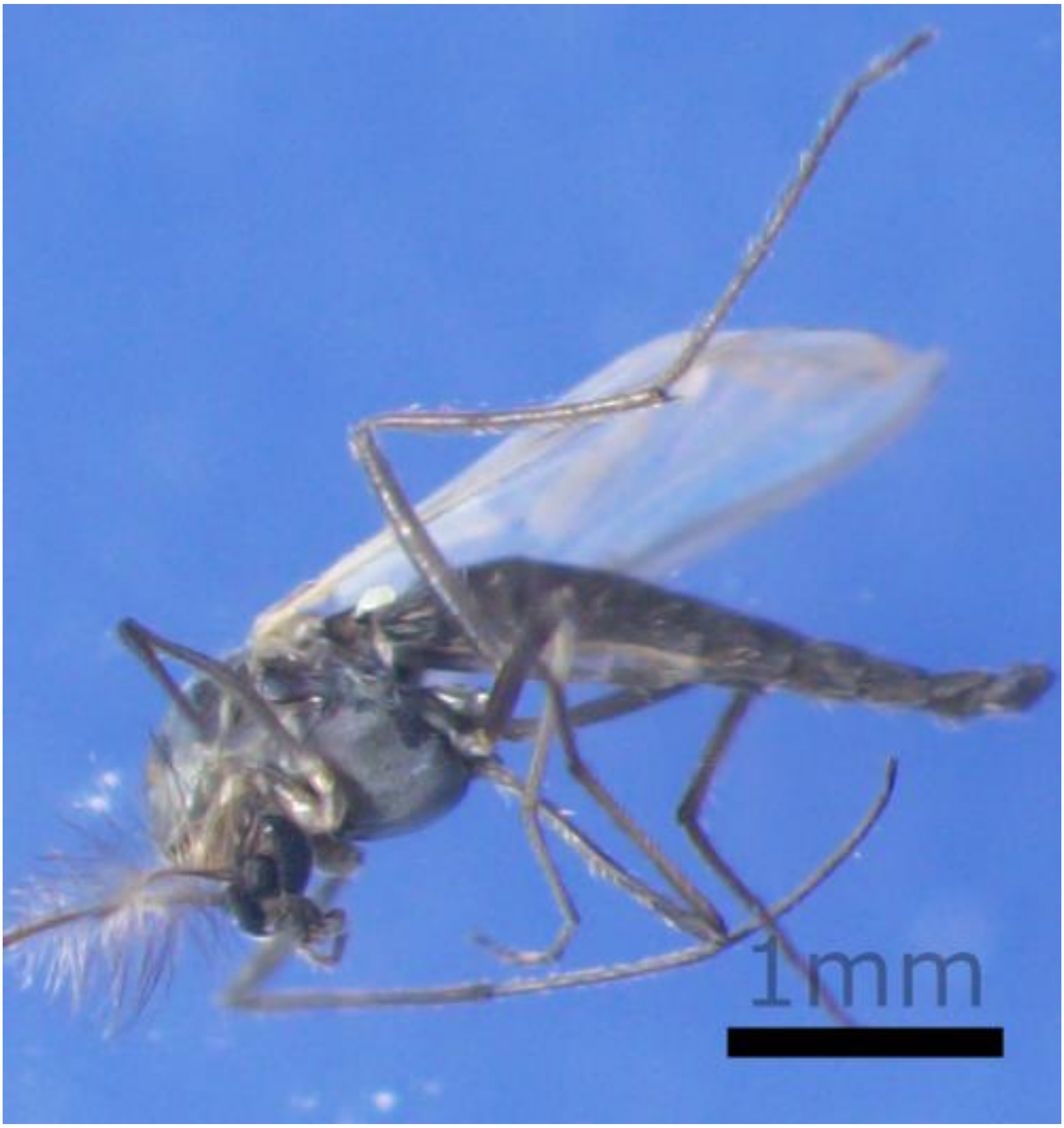

Hydrobaenus fusistylus

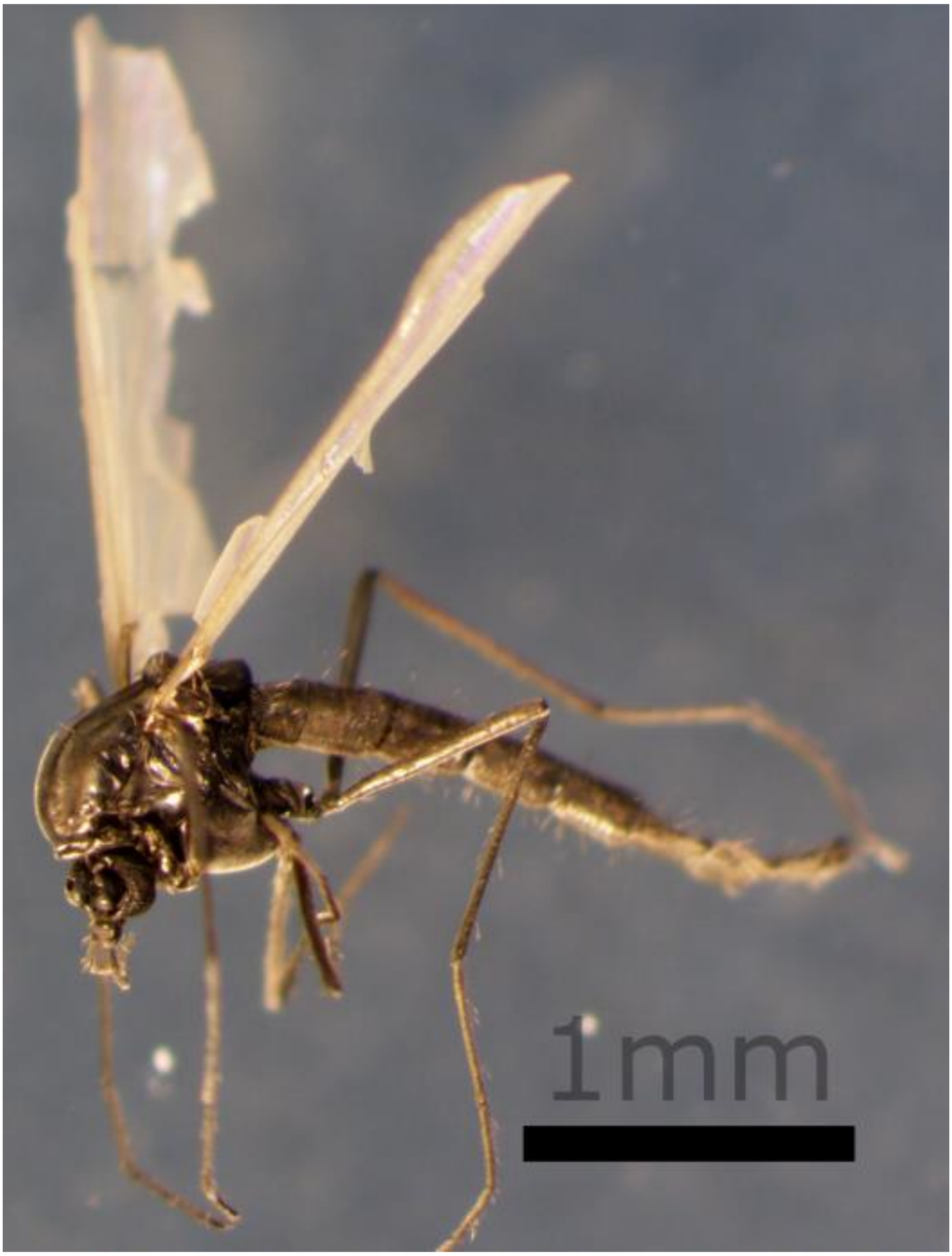

Macrocera sp.

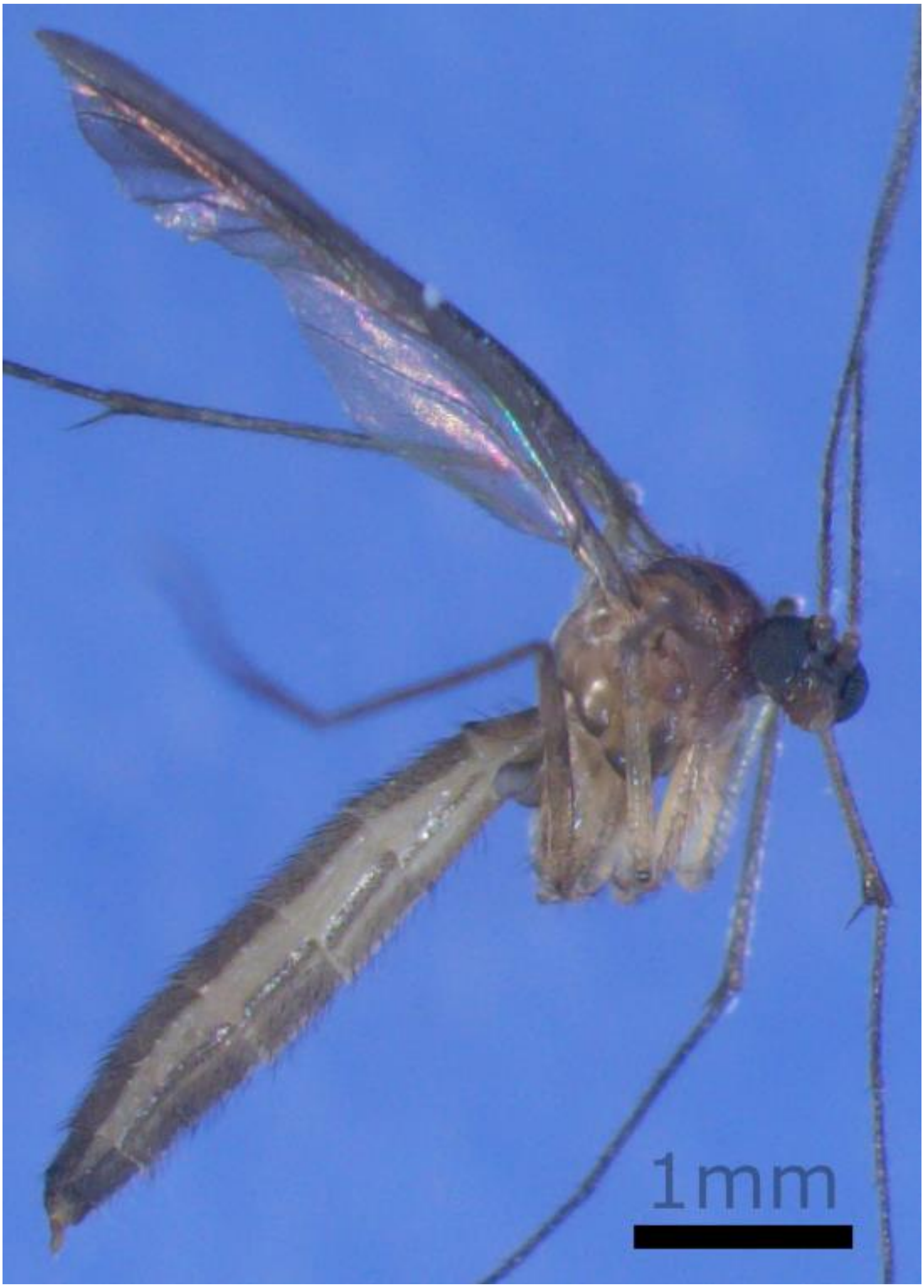

Muscidae sp.

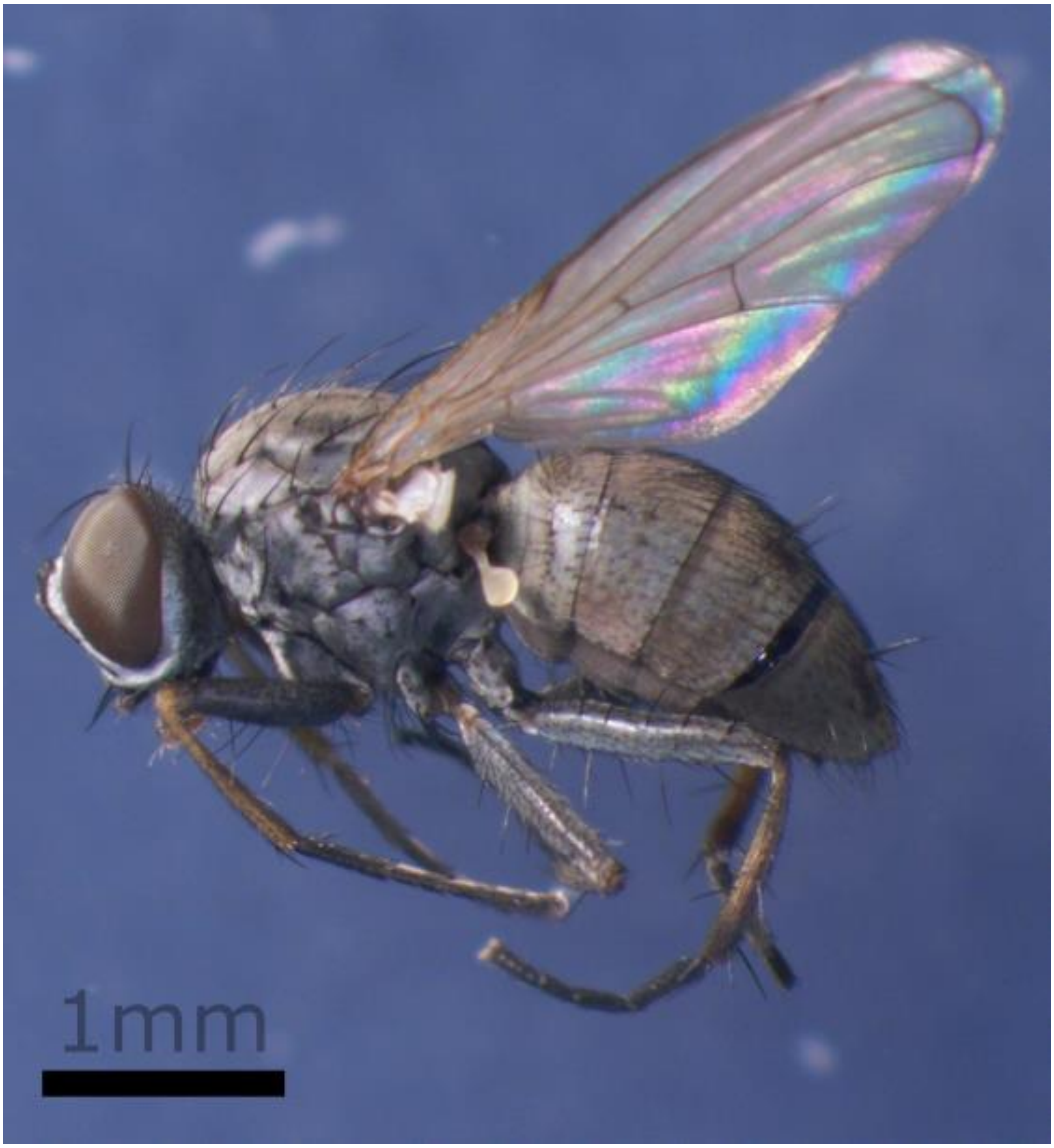

Mycetophila sp.

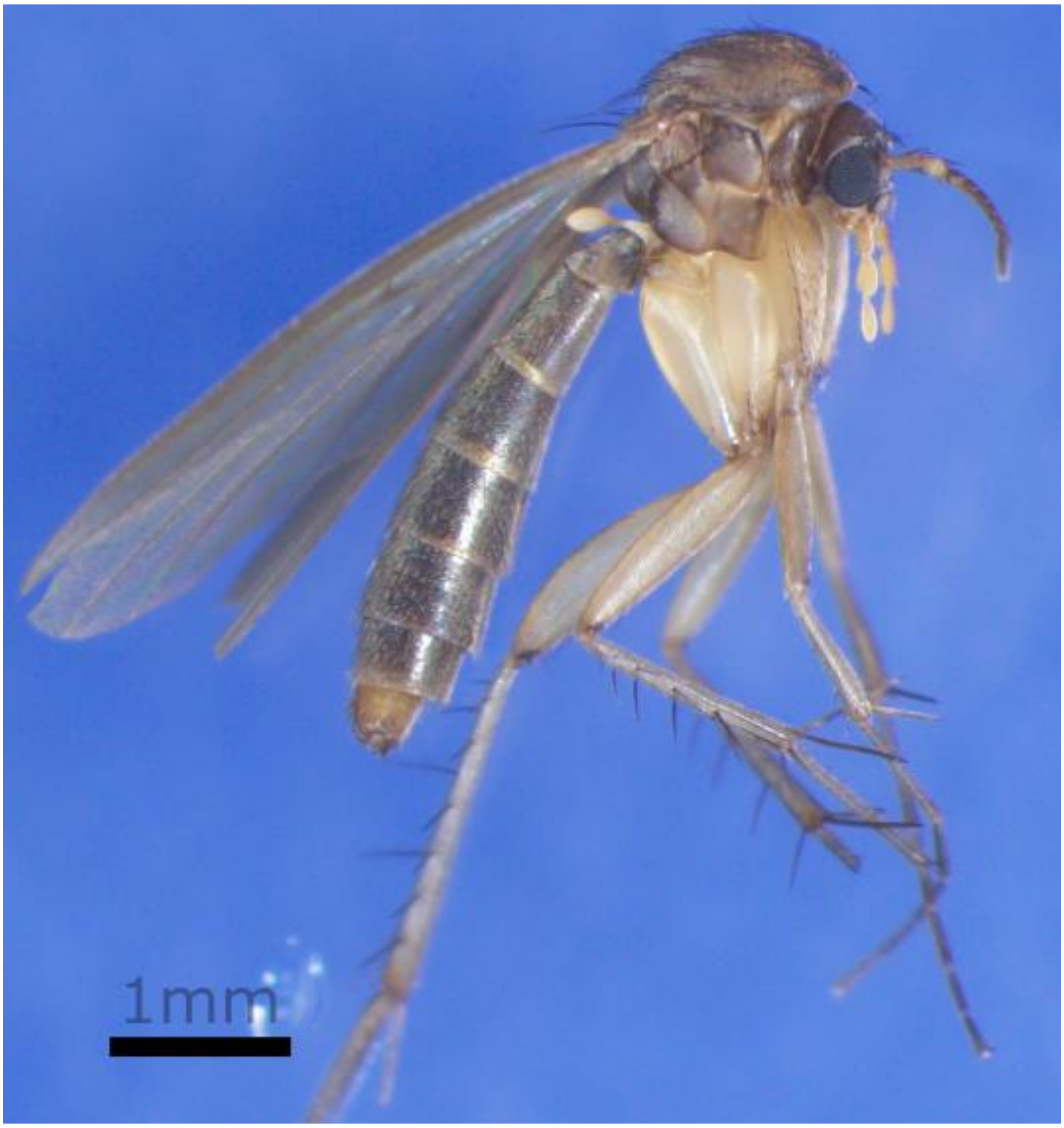

Pegomya notabilis

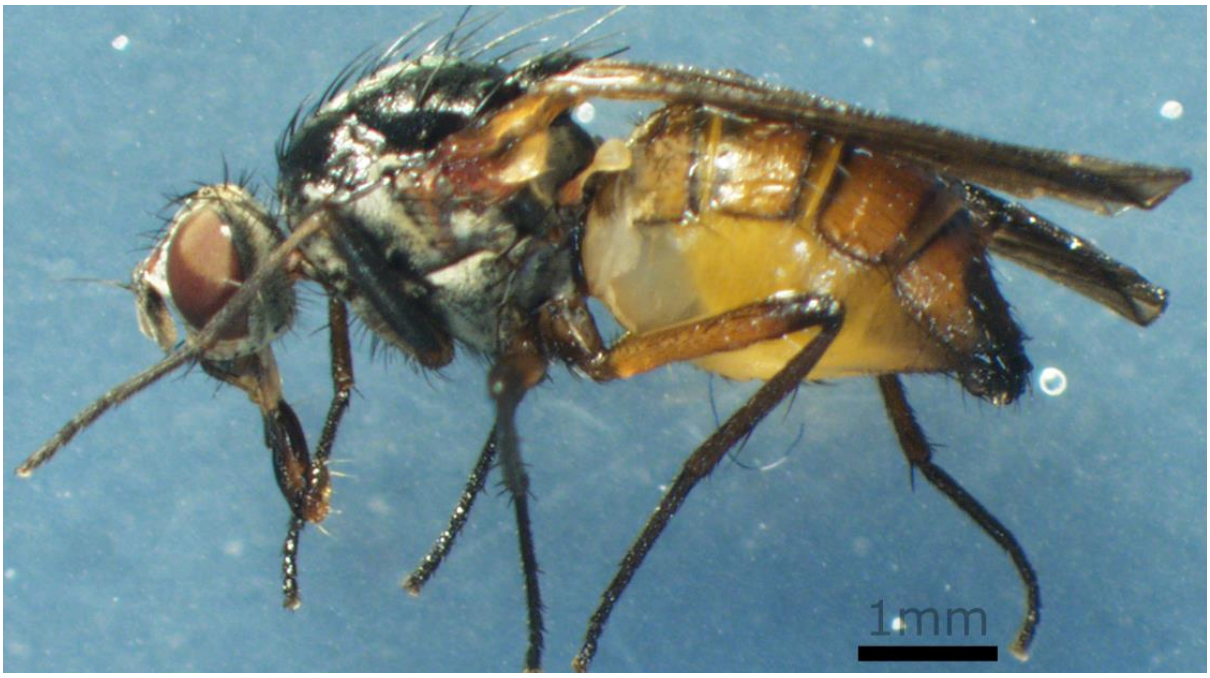

Pegomya sp.

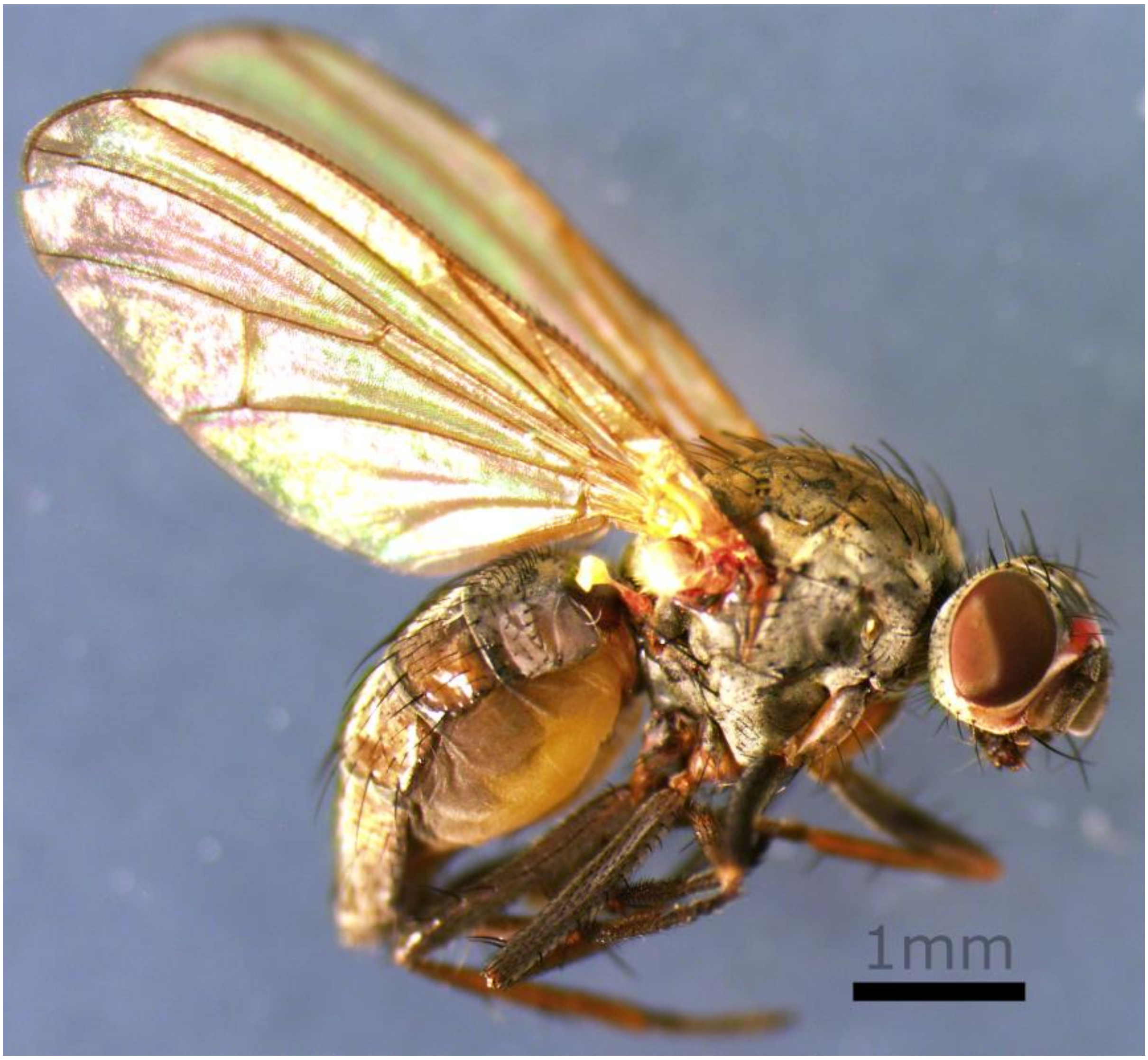

Pegomya zonata

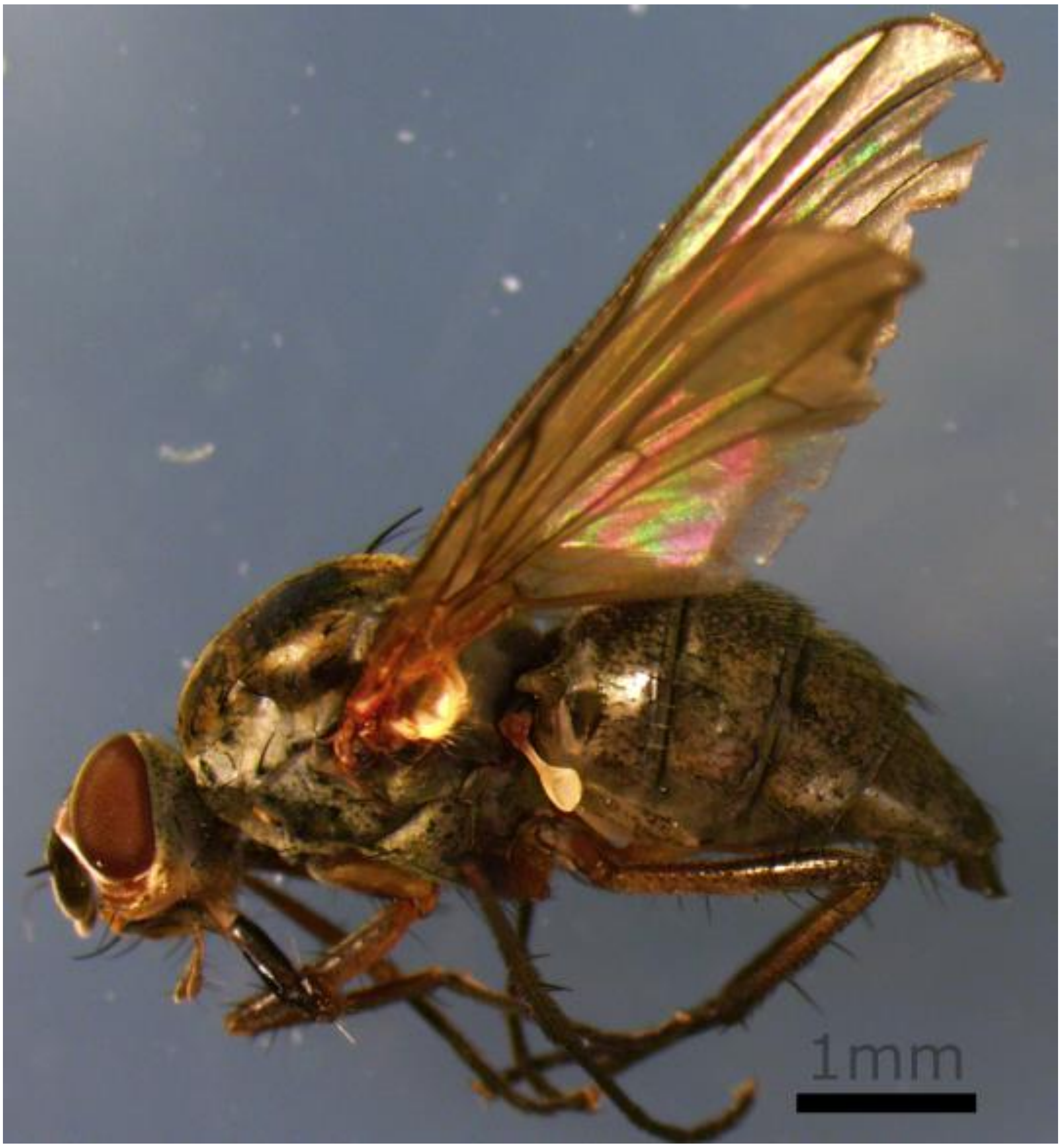

Piophila sp.

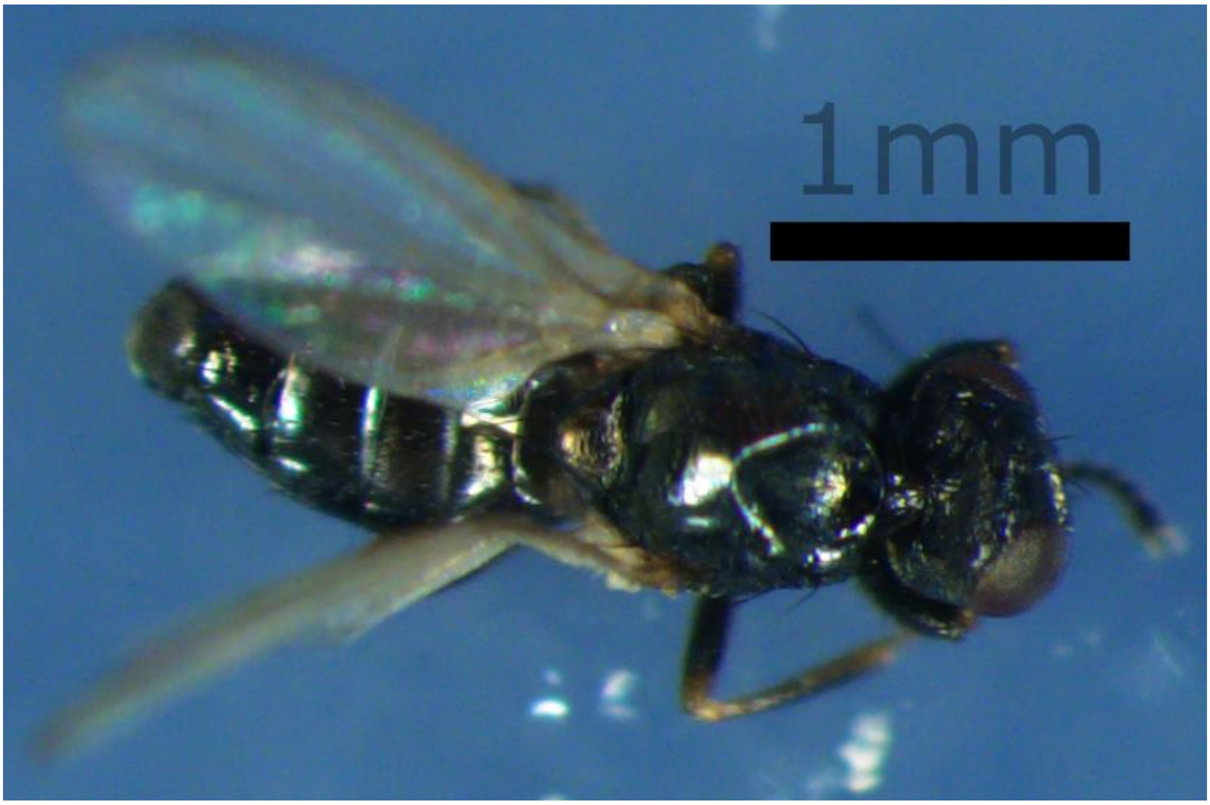

Protophormia terranovae

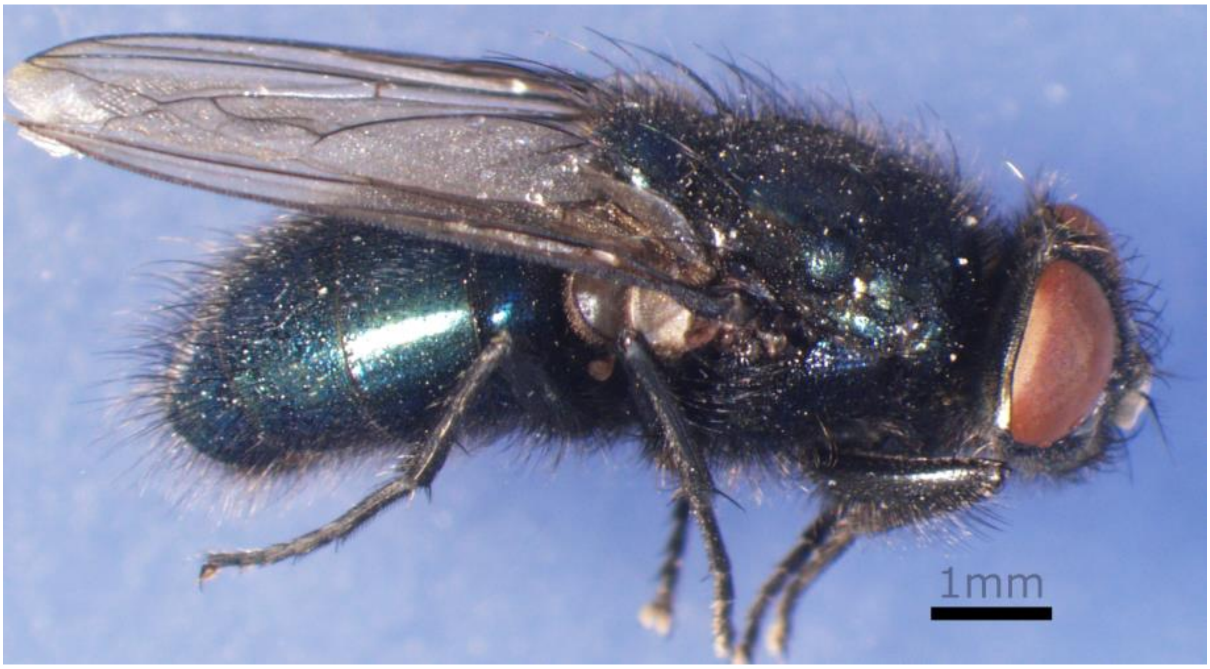

Scathophaga litorea

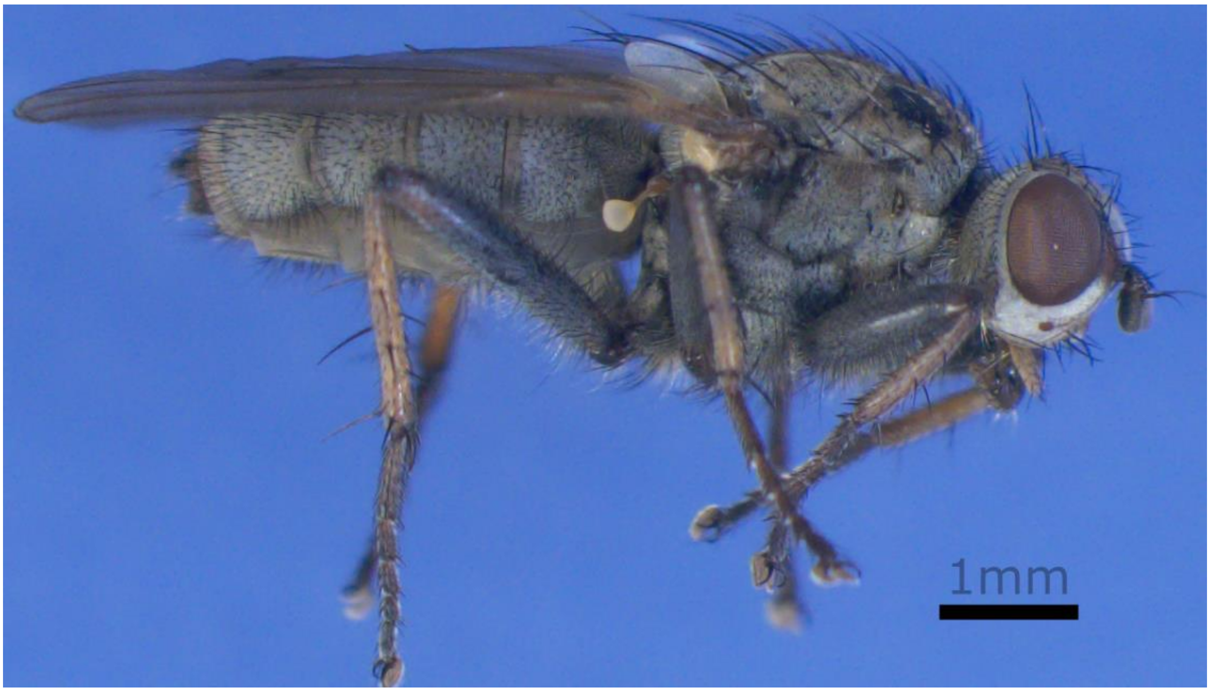

Scathophaga sp.

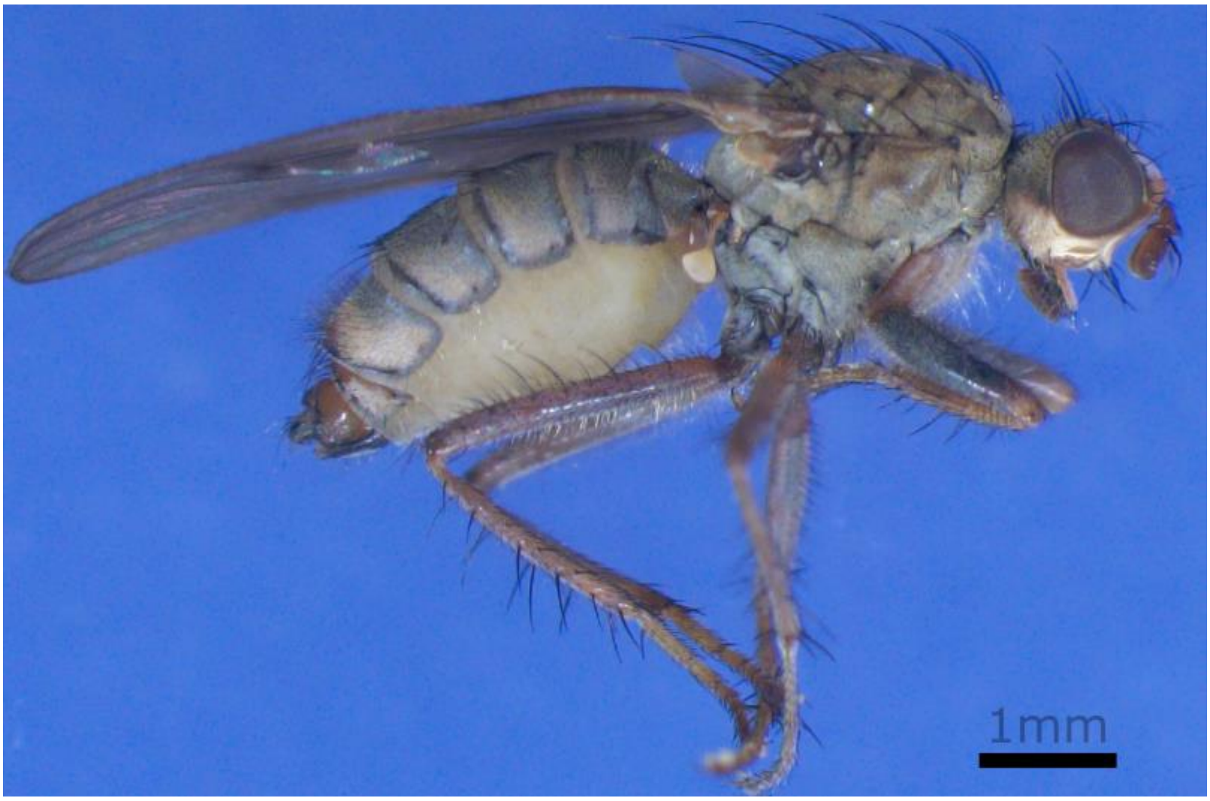

Scathophagidae sp.

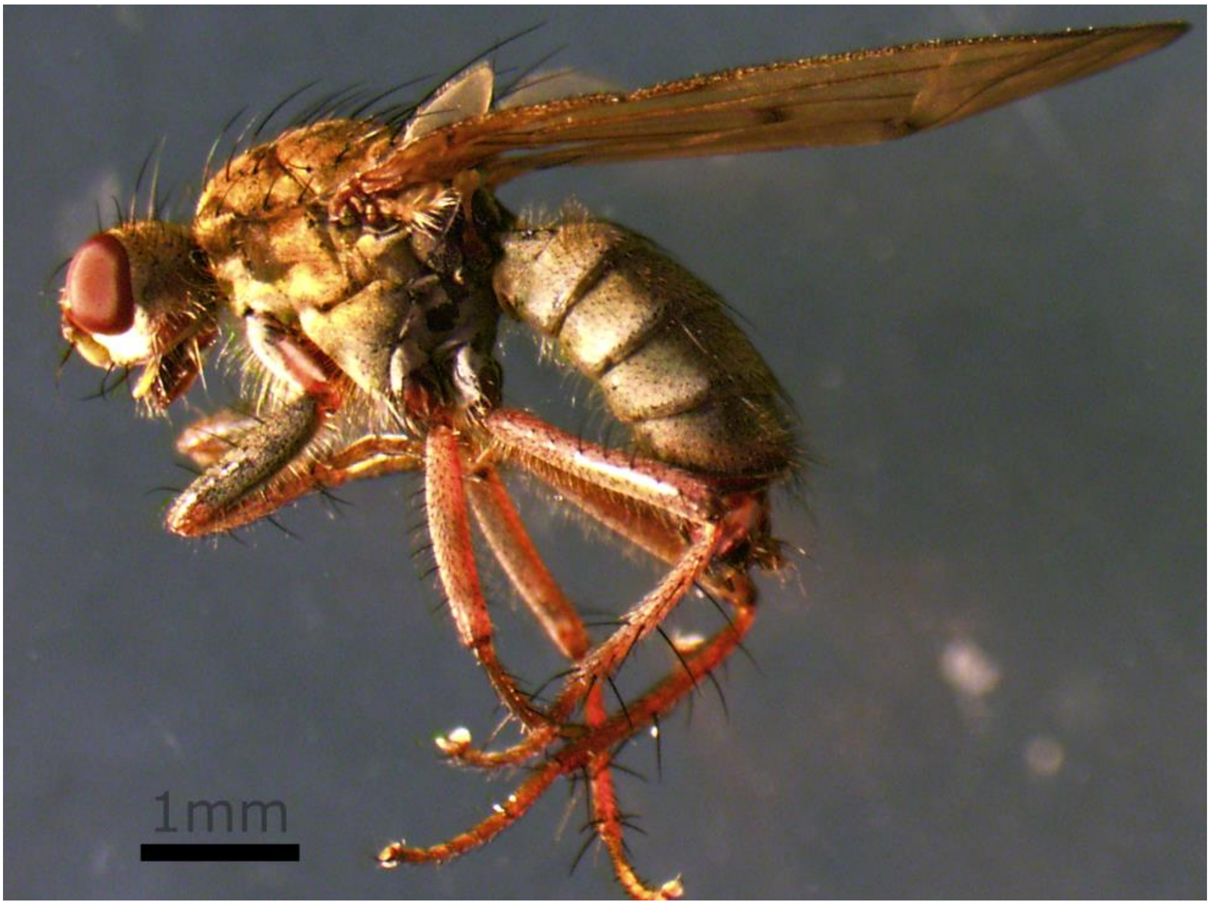

Sciaridae sp.

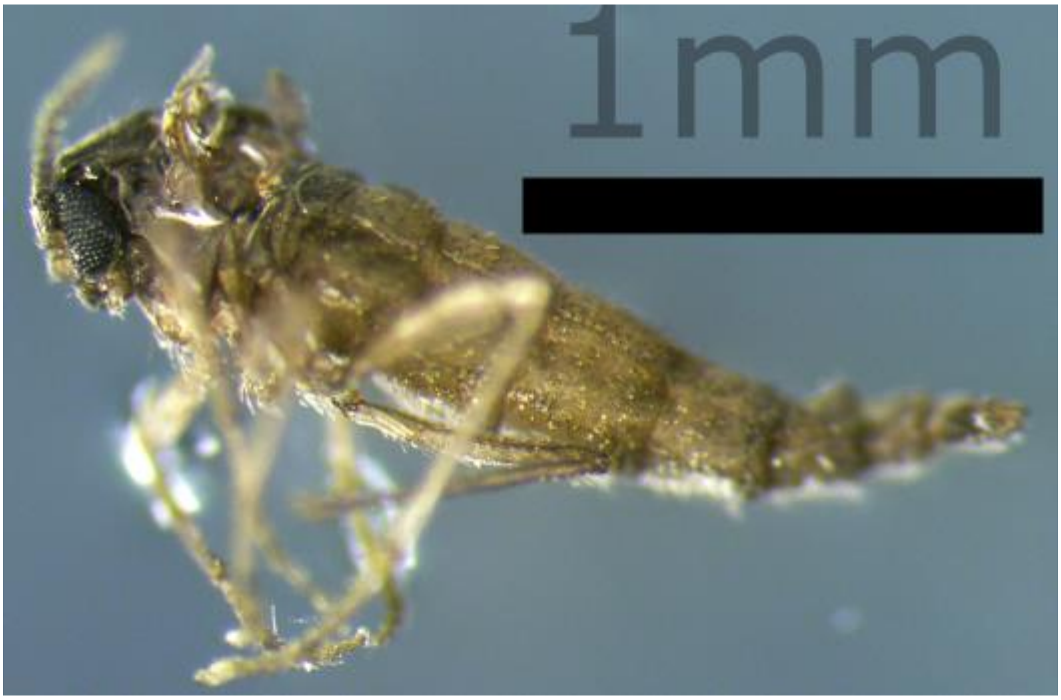

Simulium sp.

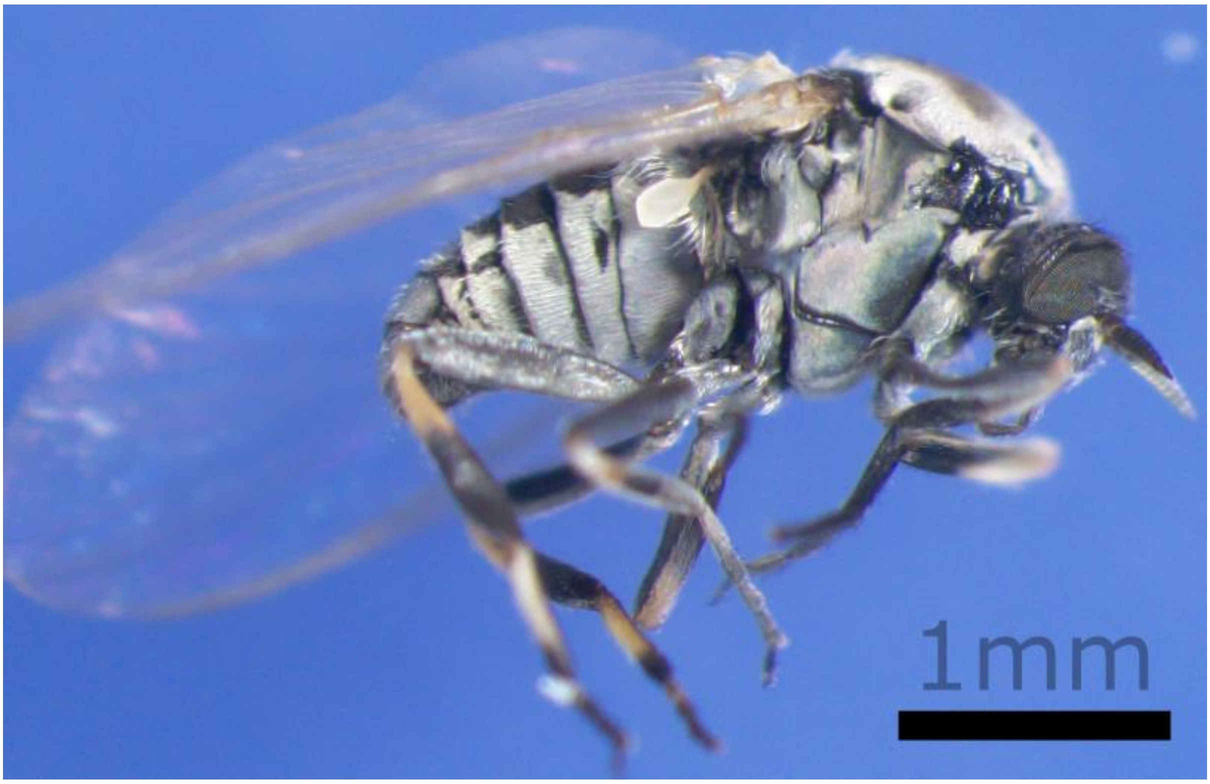

Simulium vittatum

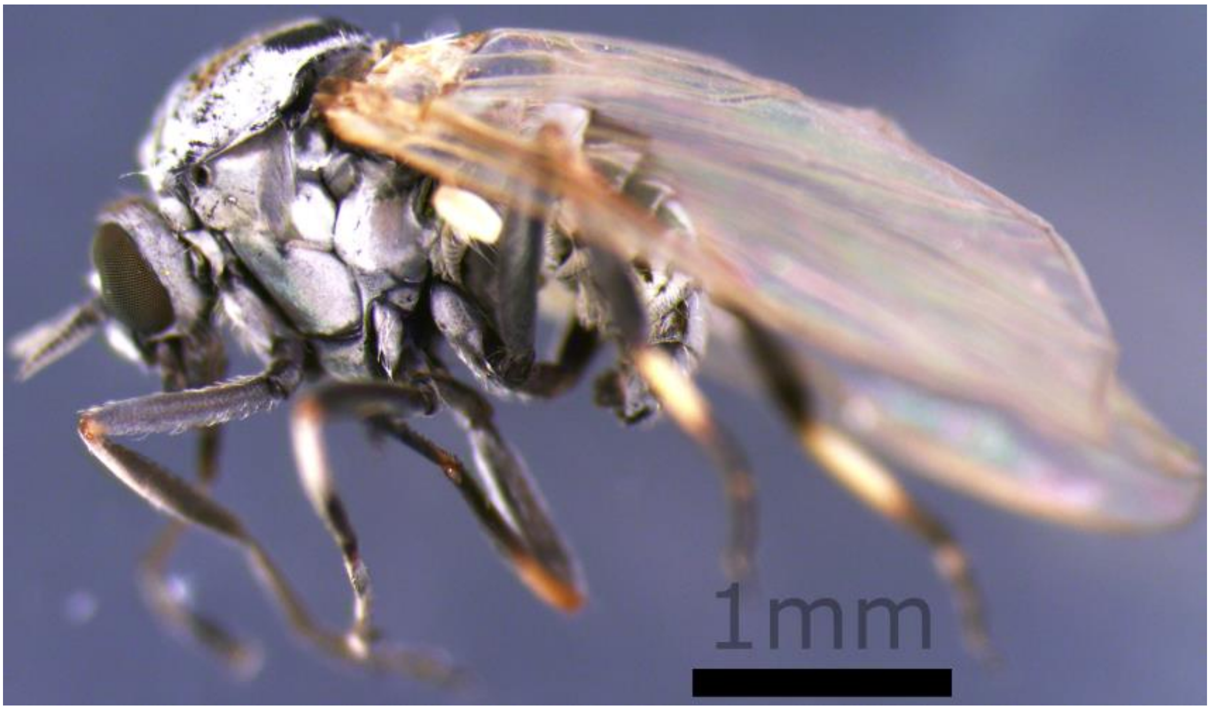

Sphaerophoria sp.

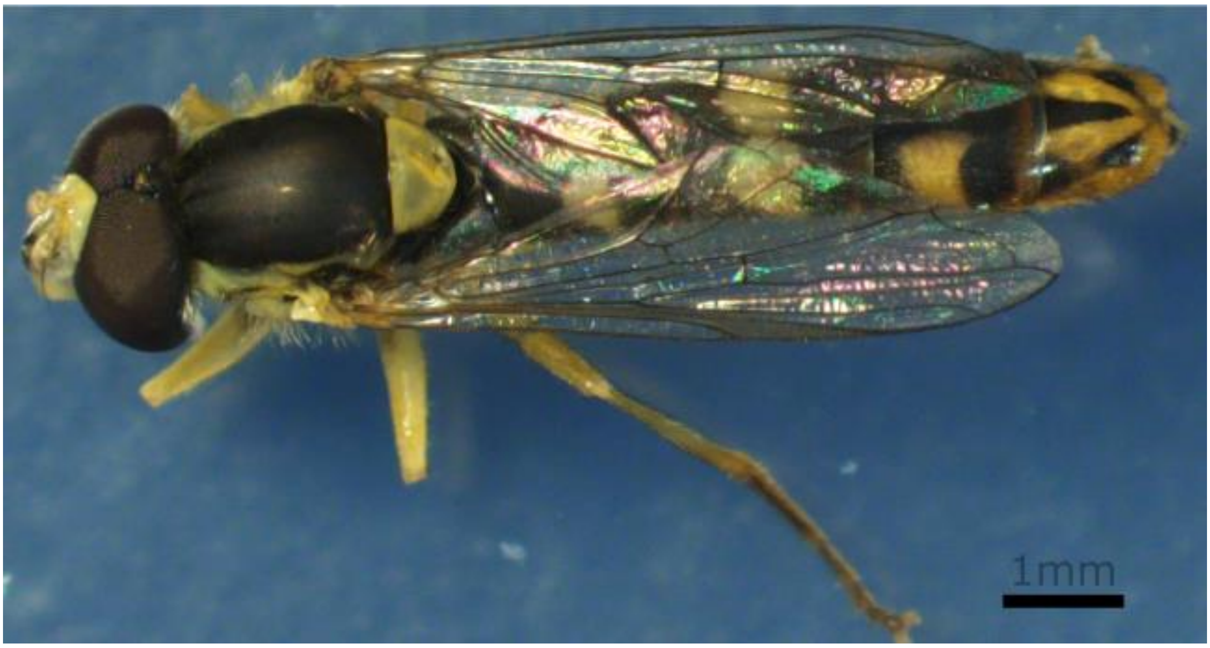

Spilogona arctica

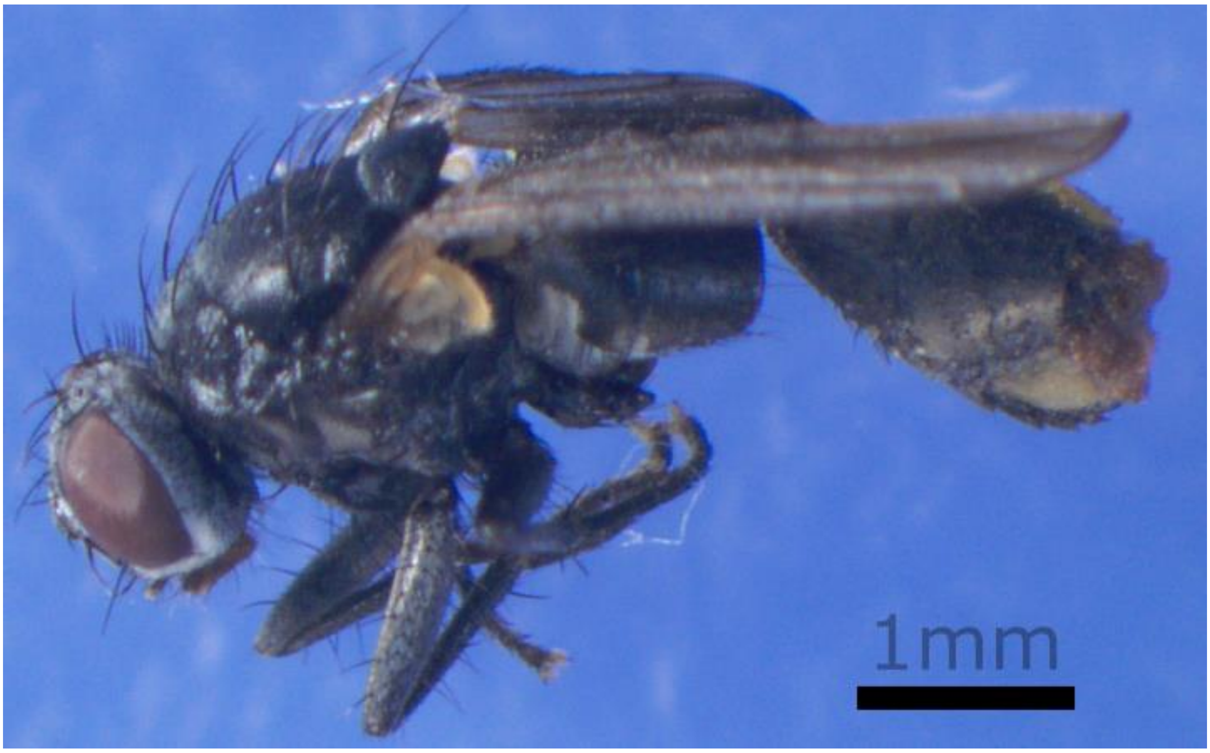

Spilogona sp.

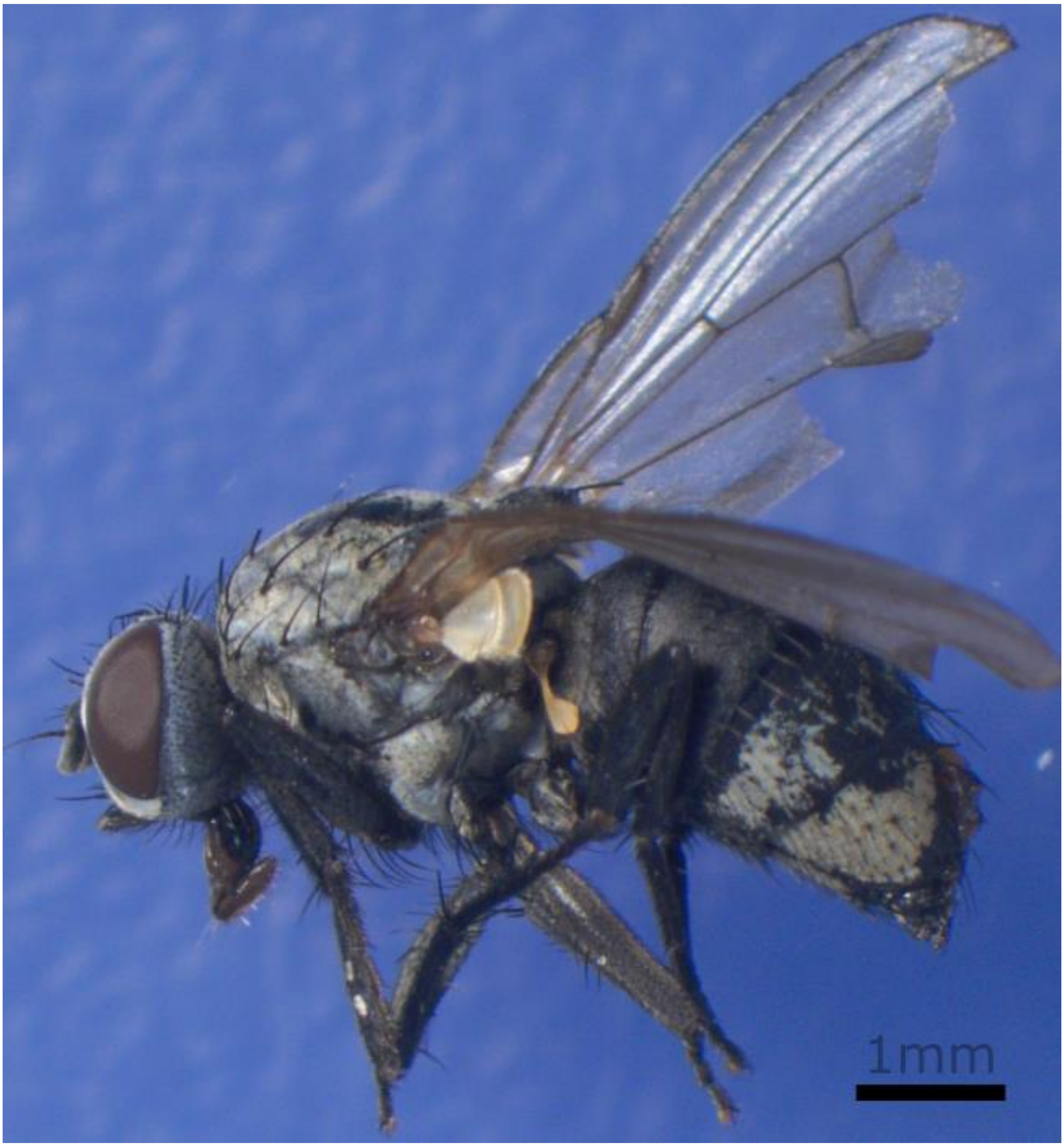

Syrphidae sp.

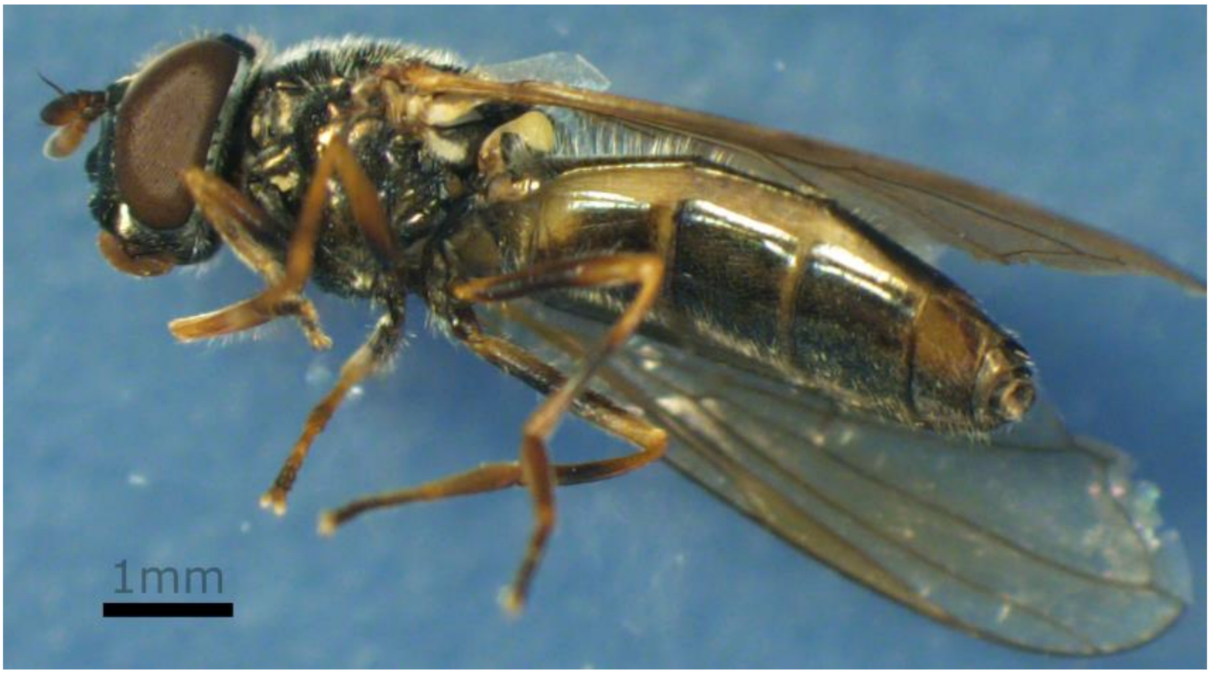

Tachina ampliforceps

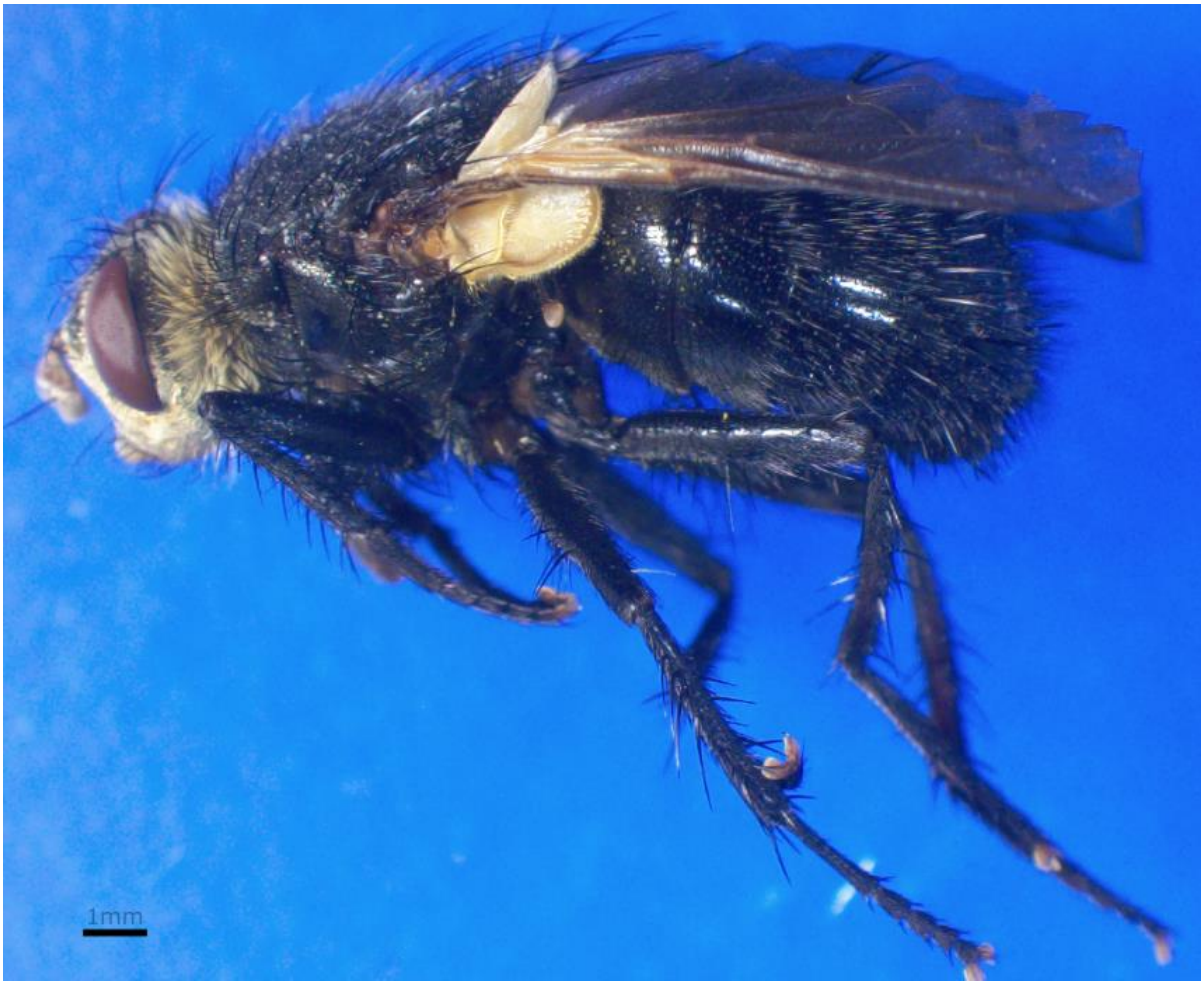

Entomobryomorpha

Lepidocyrtus sp.

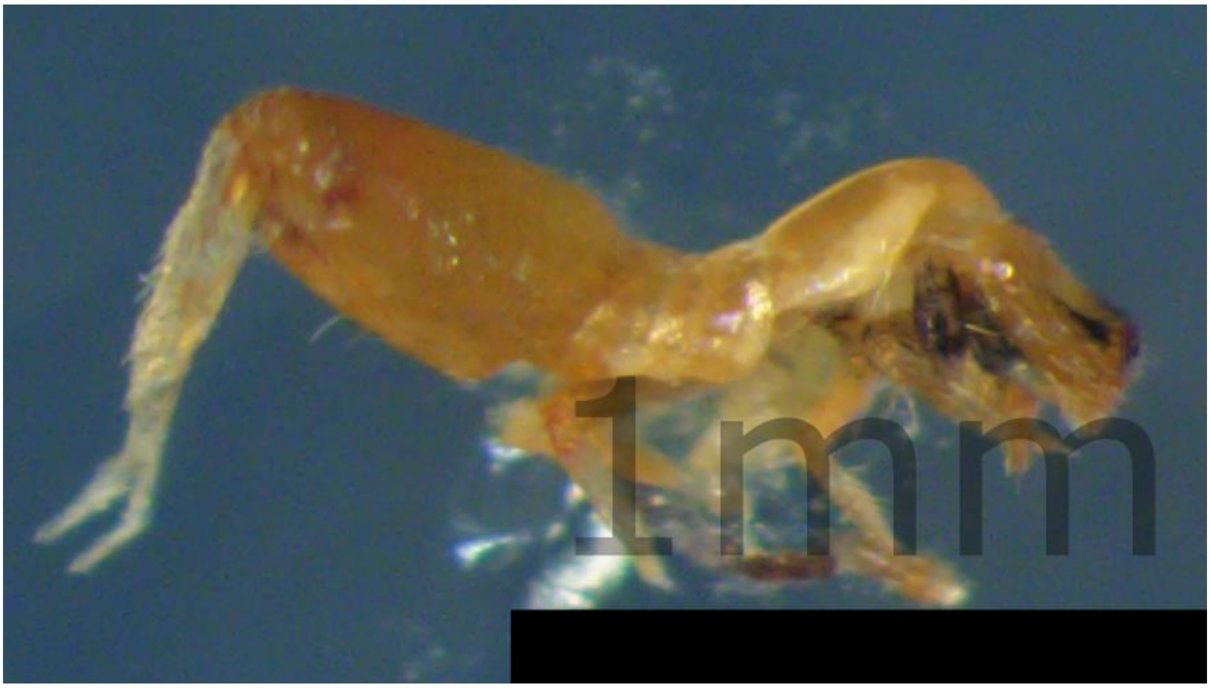

Hemiptera

Aphididae sp.

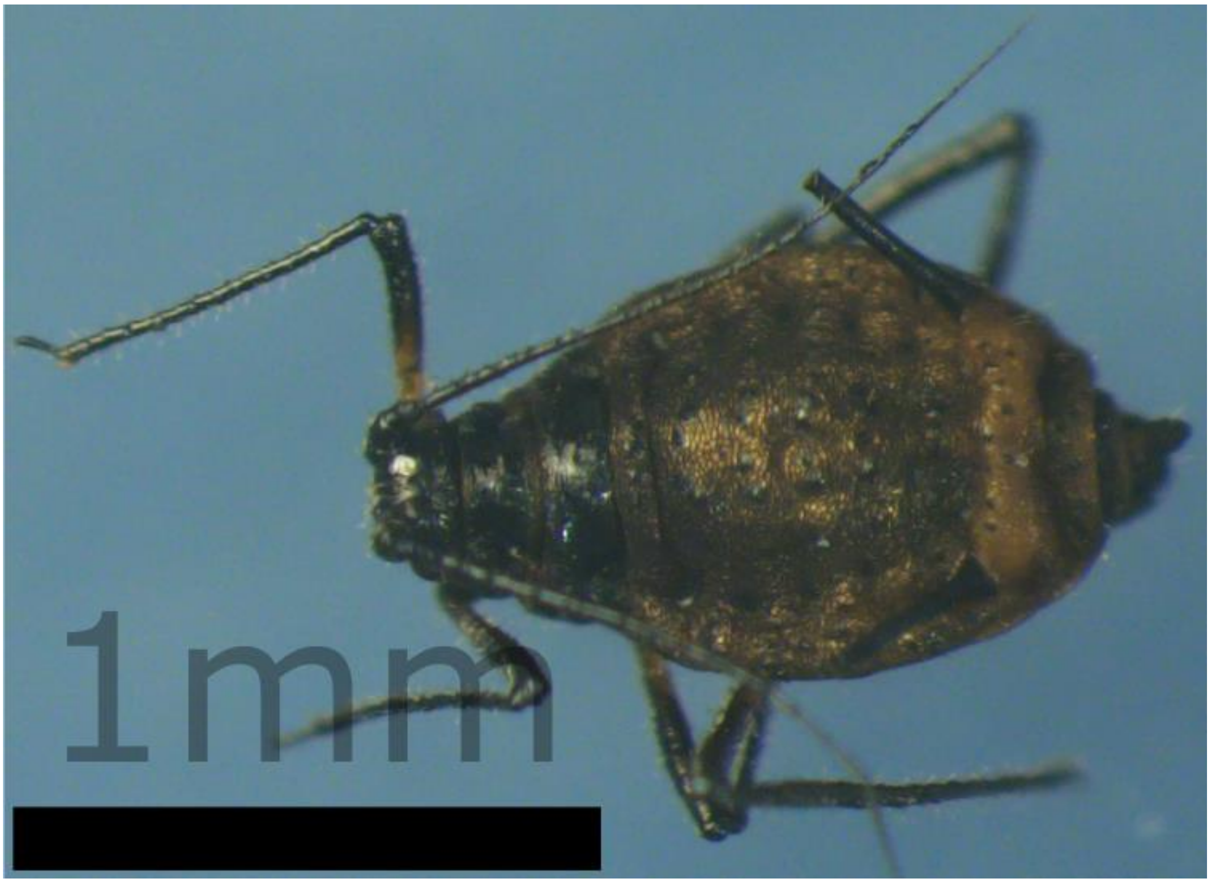

Cavariella sp.

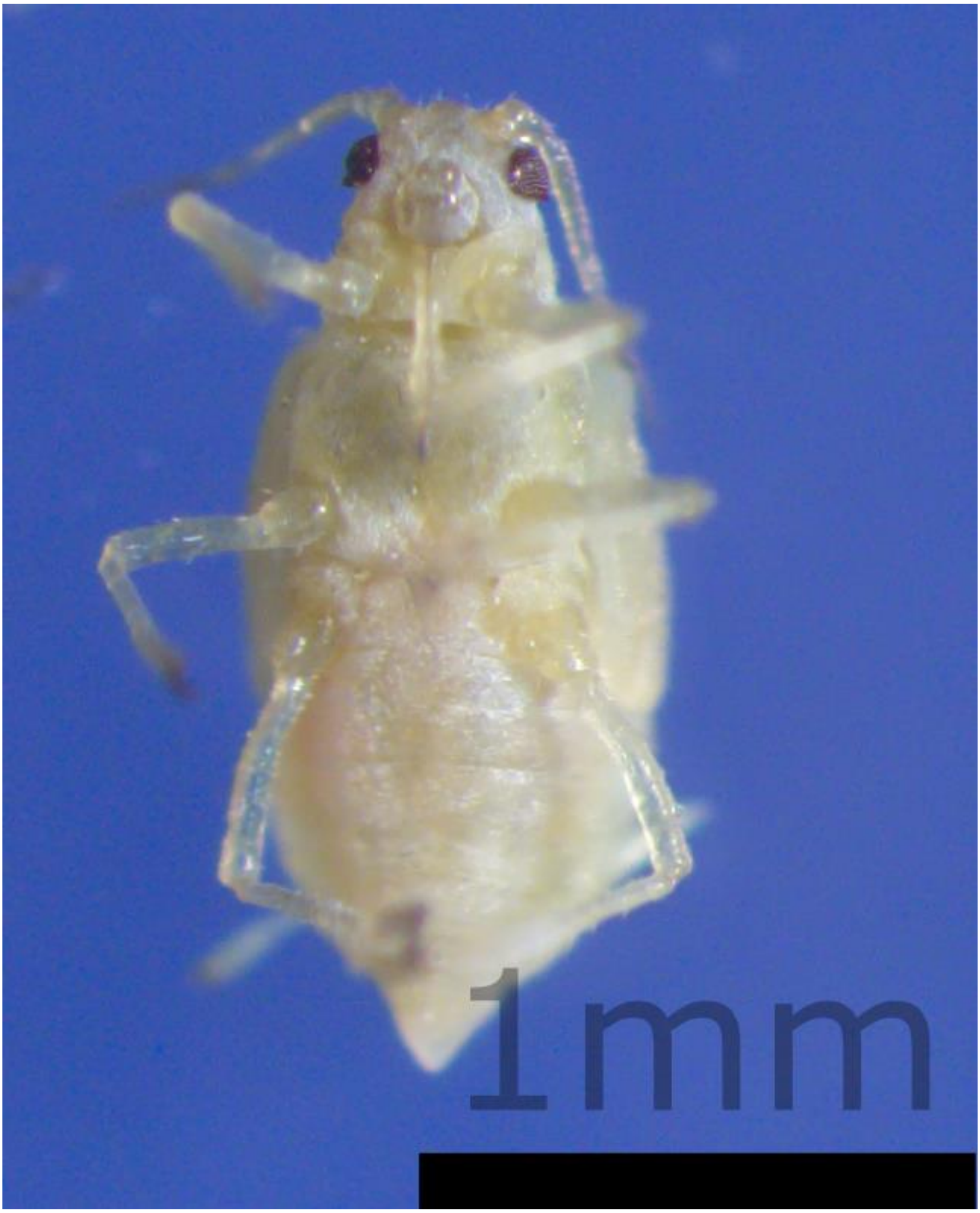

Euceraphis punctipennis

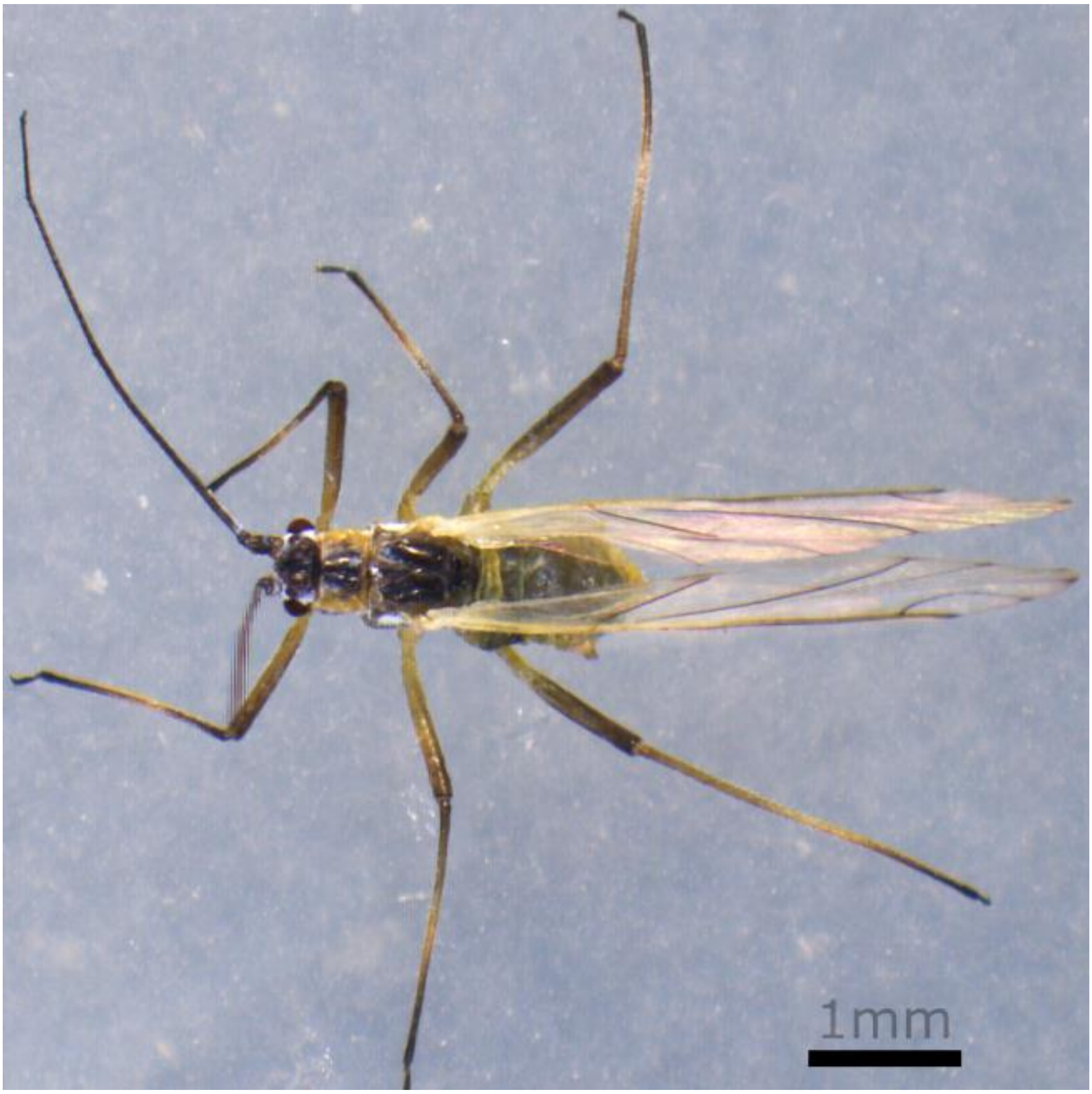

Euceraphis sp

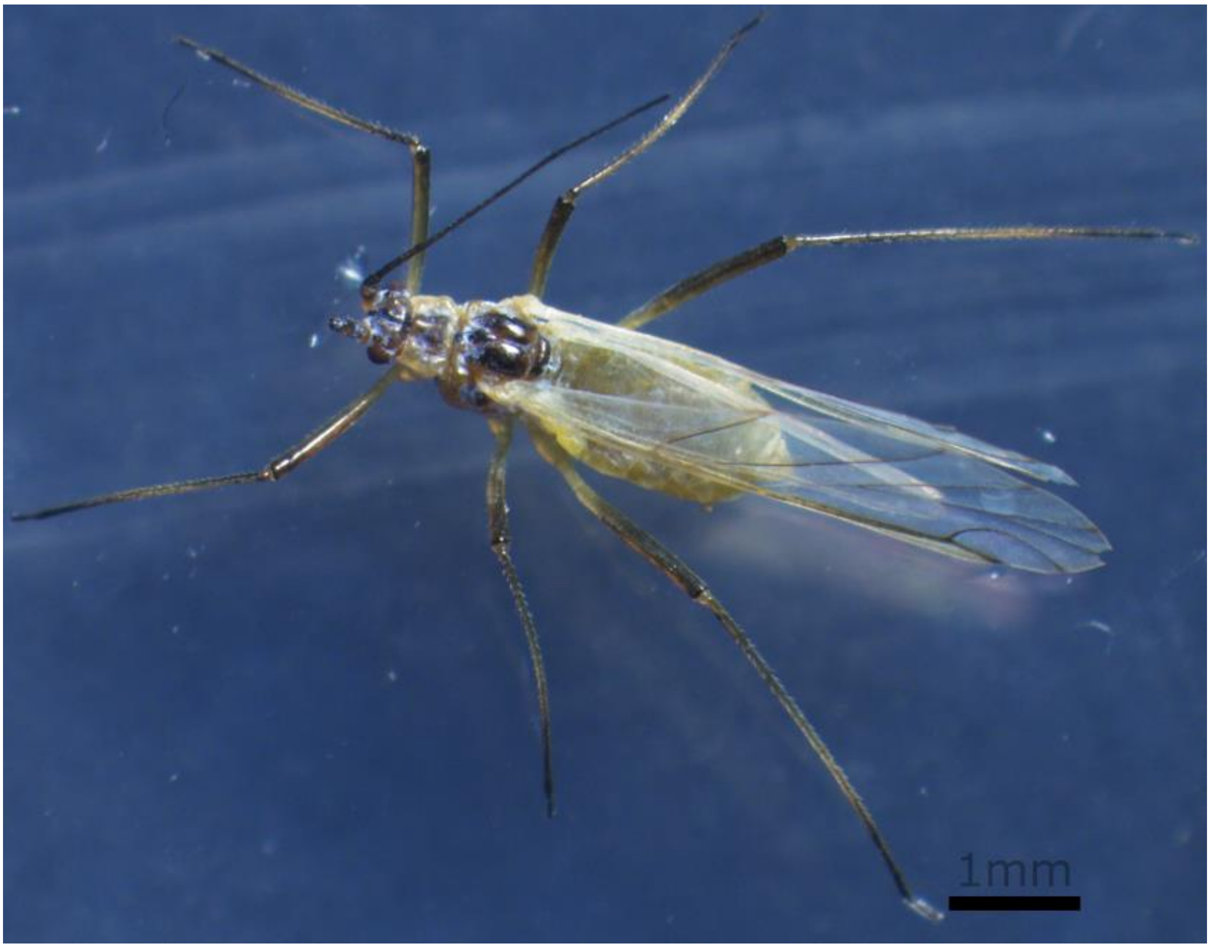

Myzodium modestum

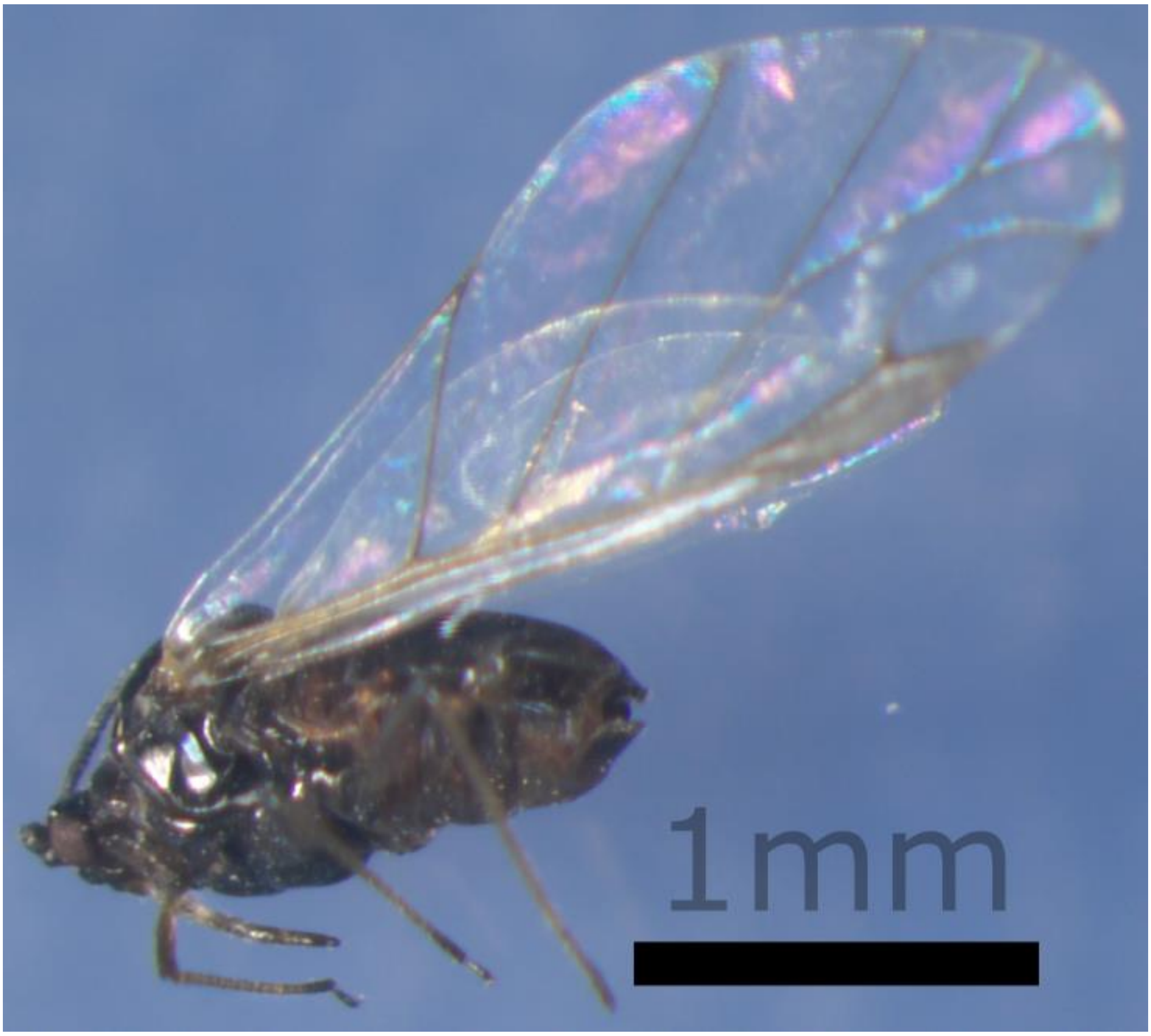

Nabis flavomarginatus

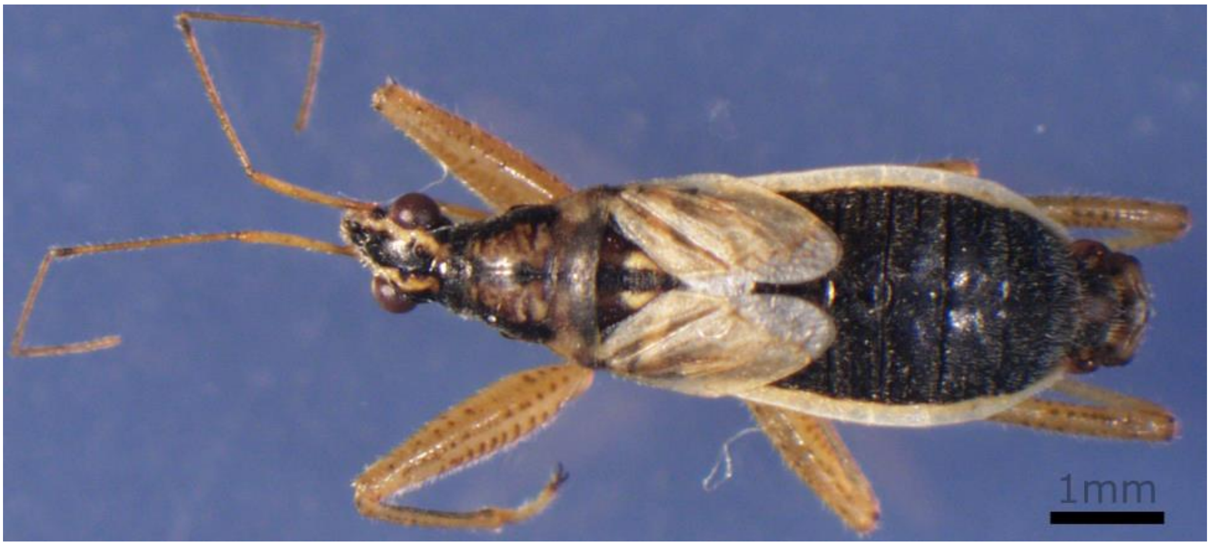

Nysius groenlandicus

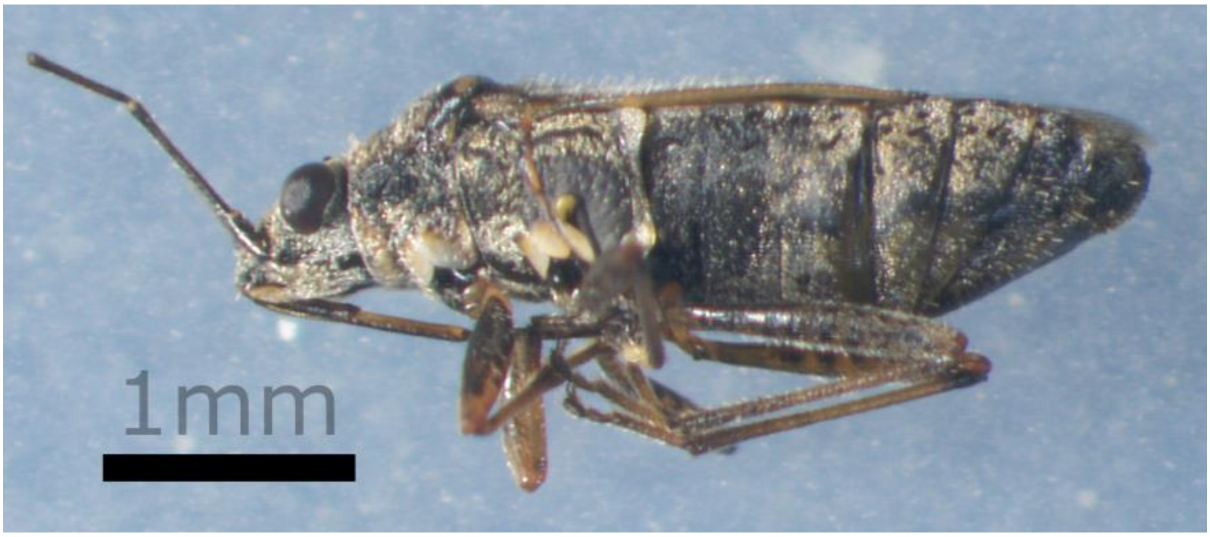

Psammotettix lividellus

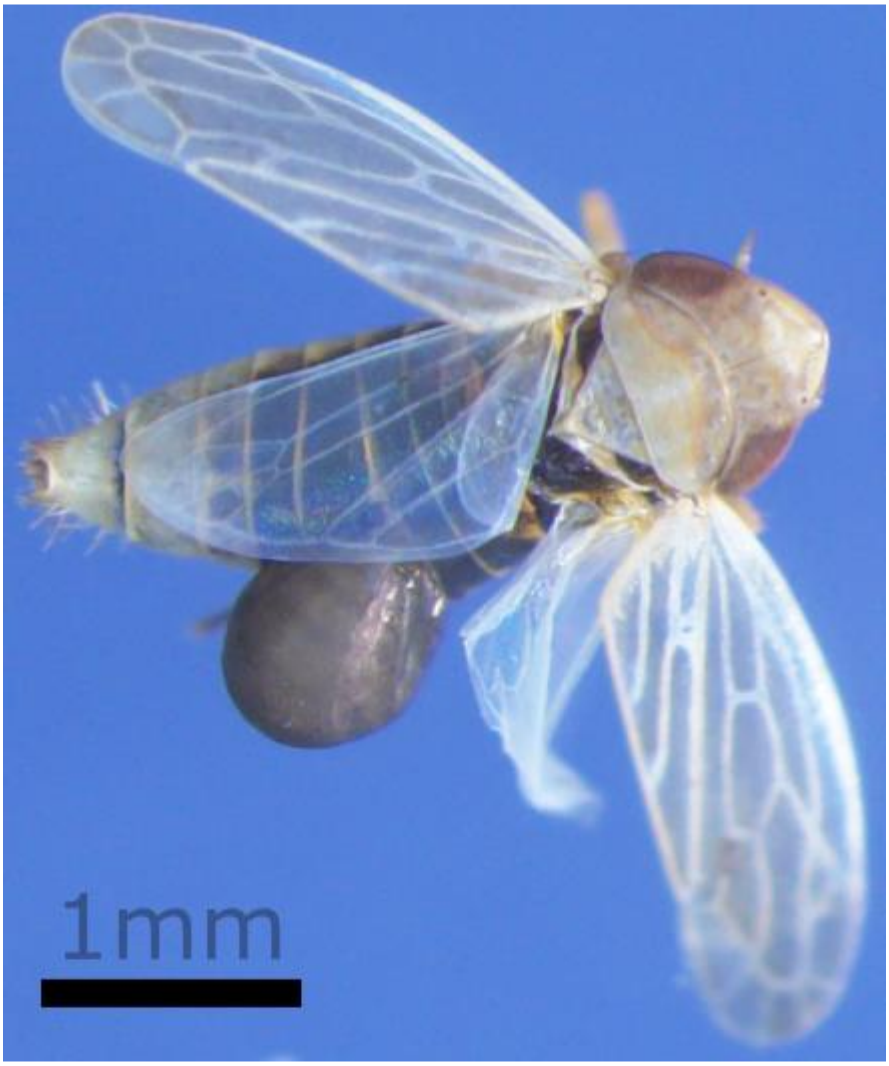

Psyllidae sp.

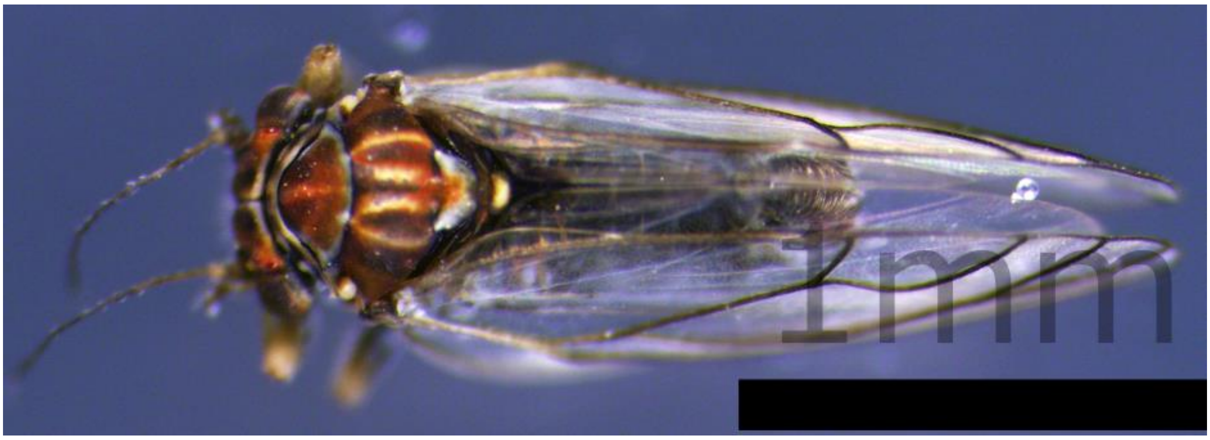

Pterocomma sp.

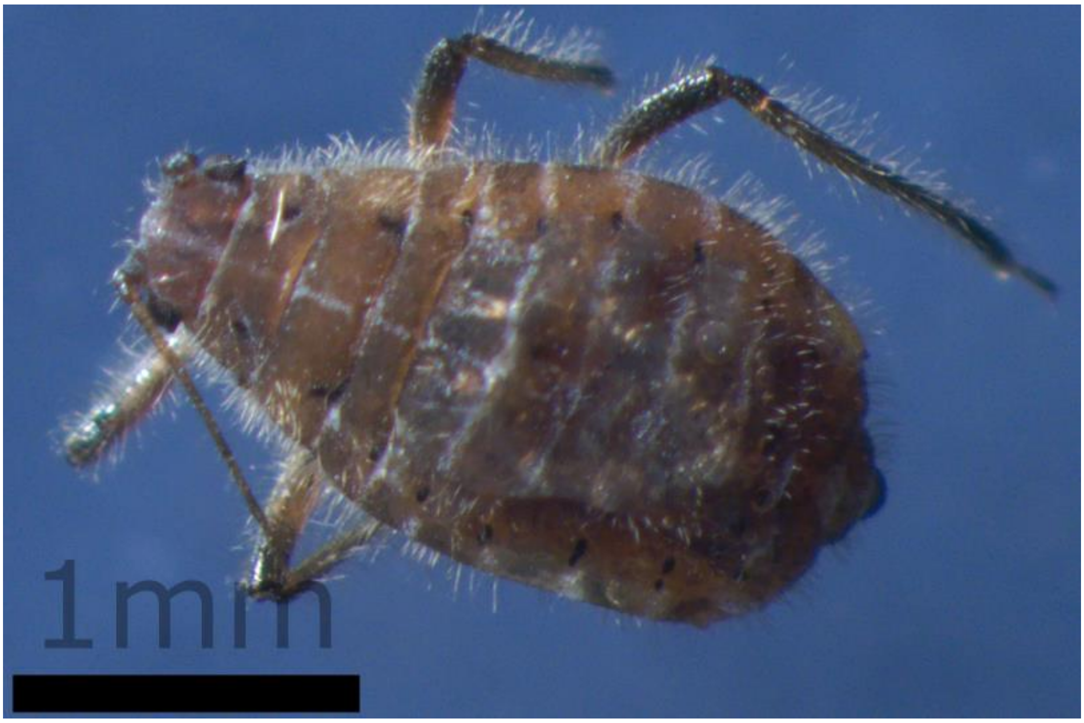

Utamphorophora sp.

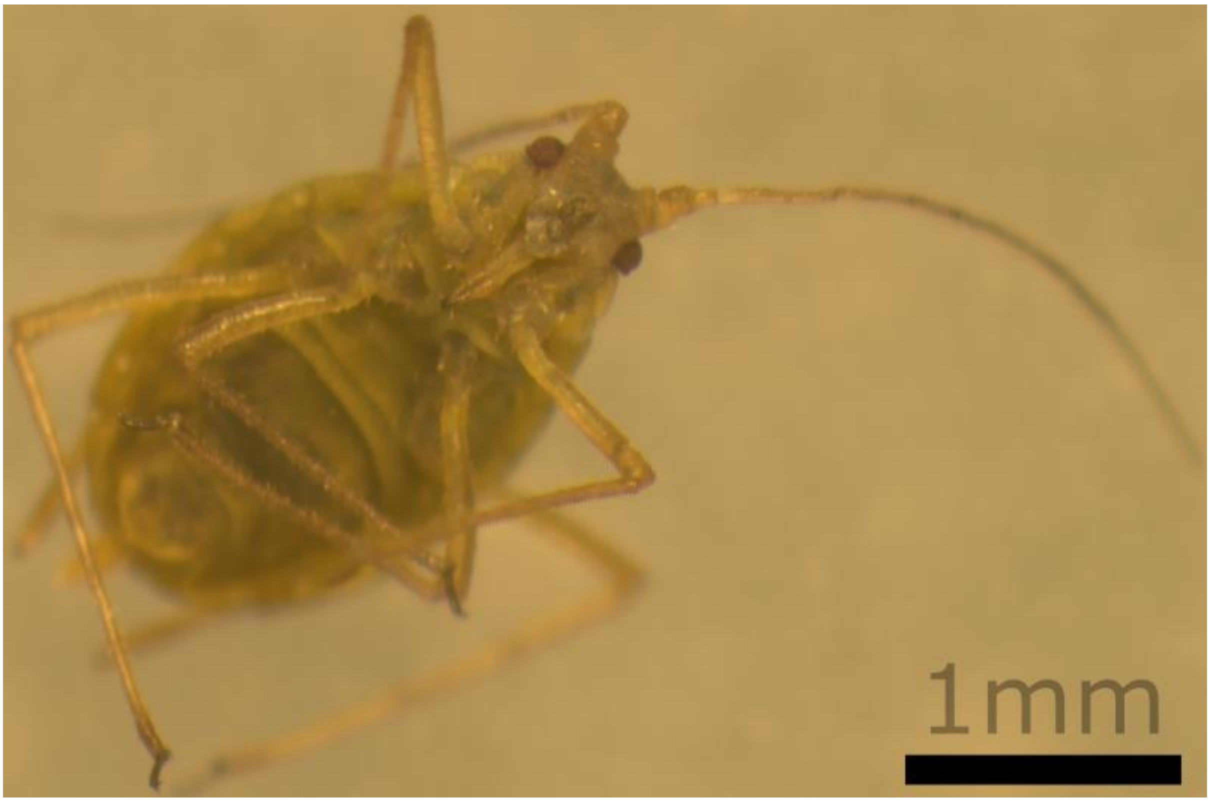

Hymenoptera

Brachonidae sp.

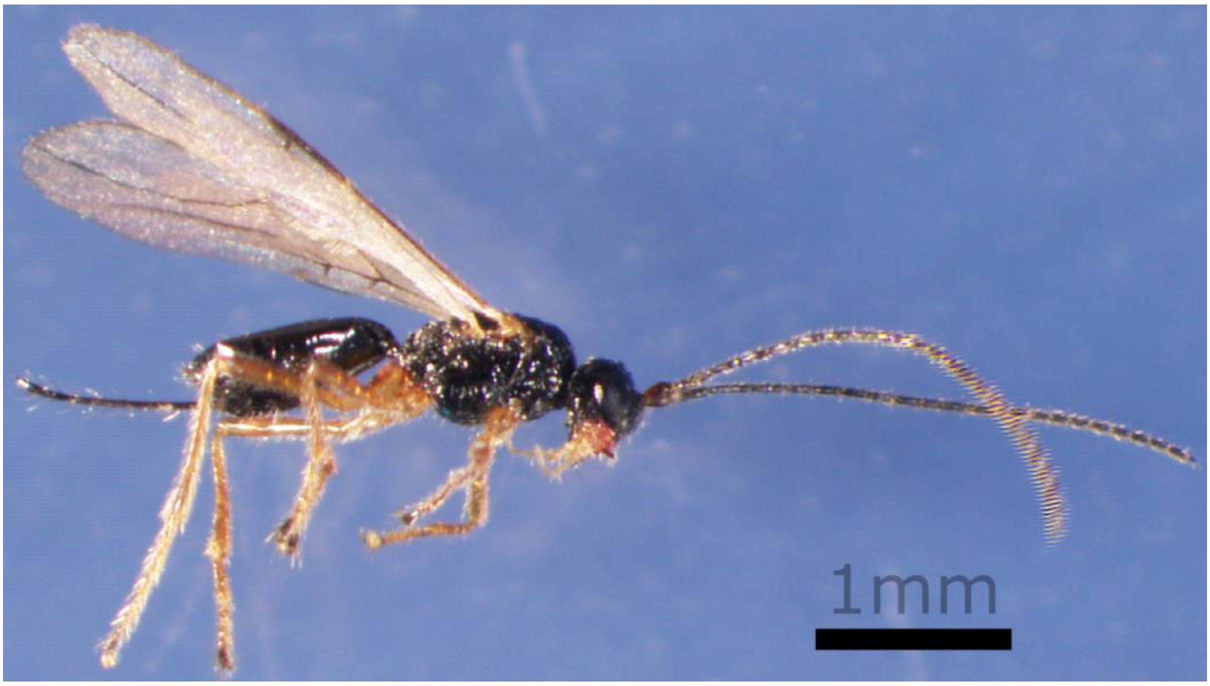

Campoletis horstmanni

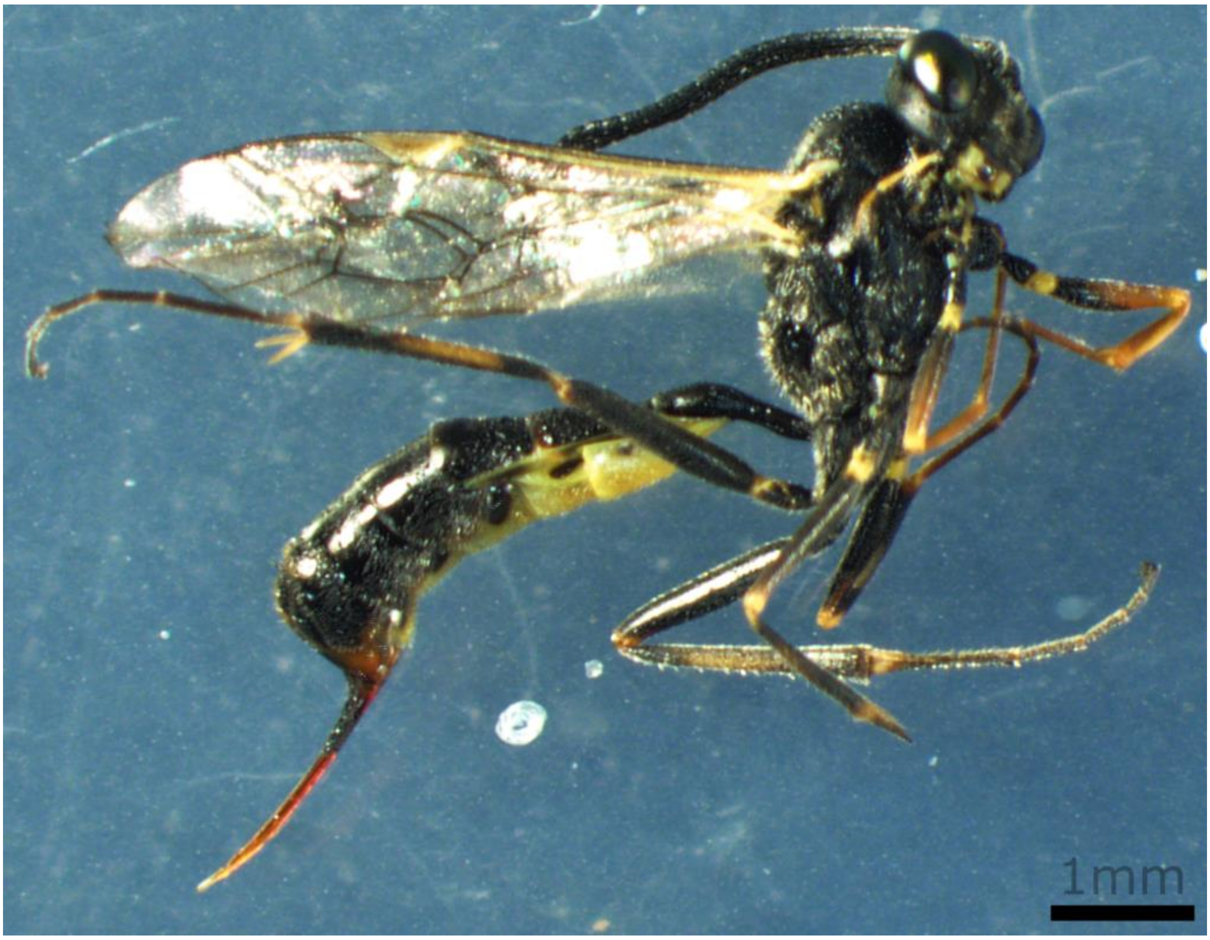

Campopleginae sp.

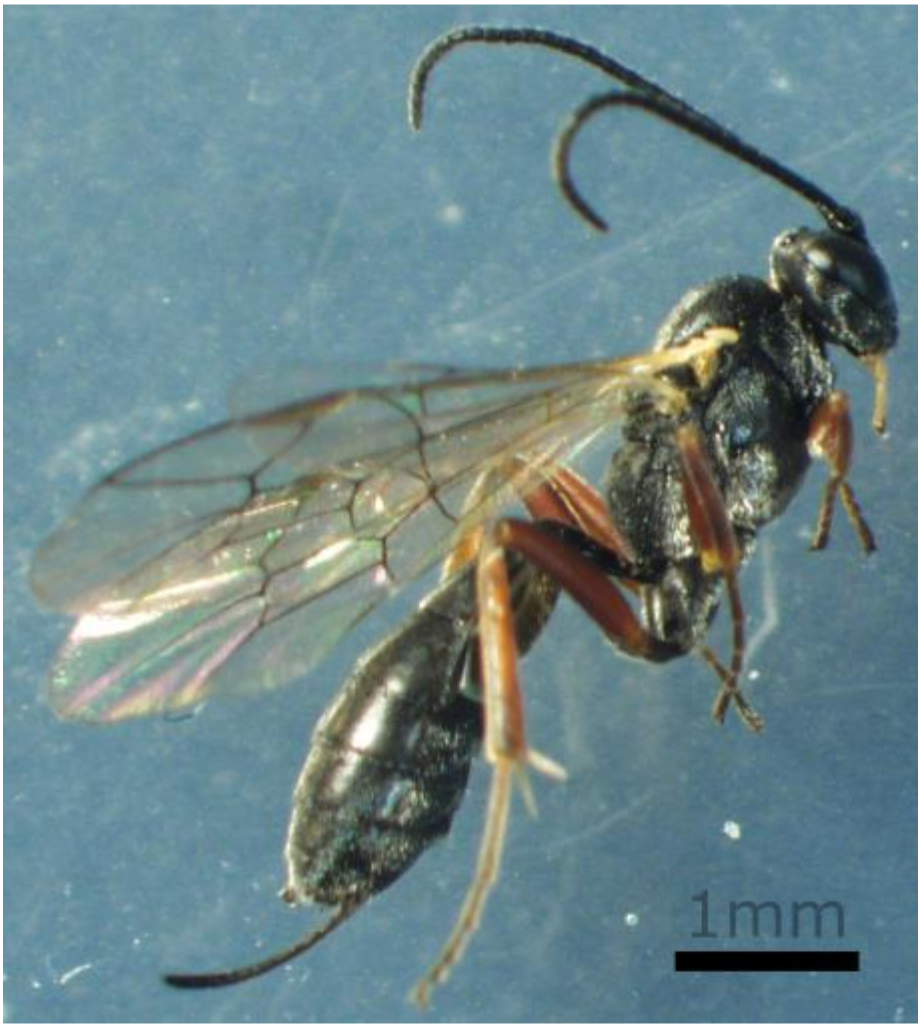

Hymenoptera sp.

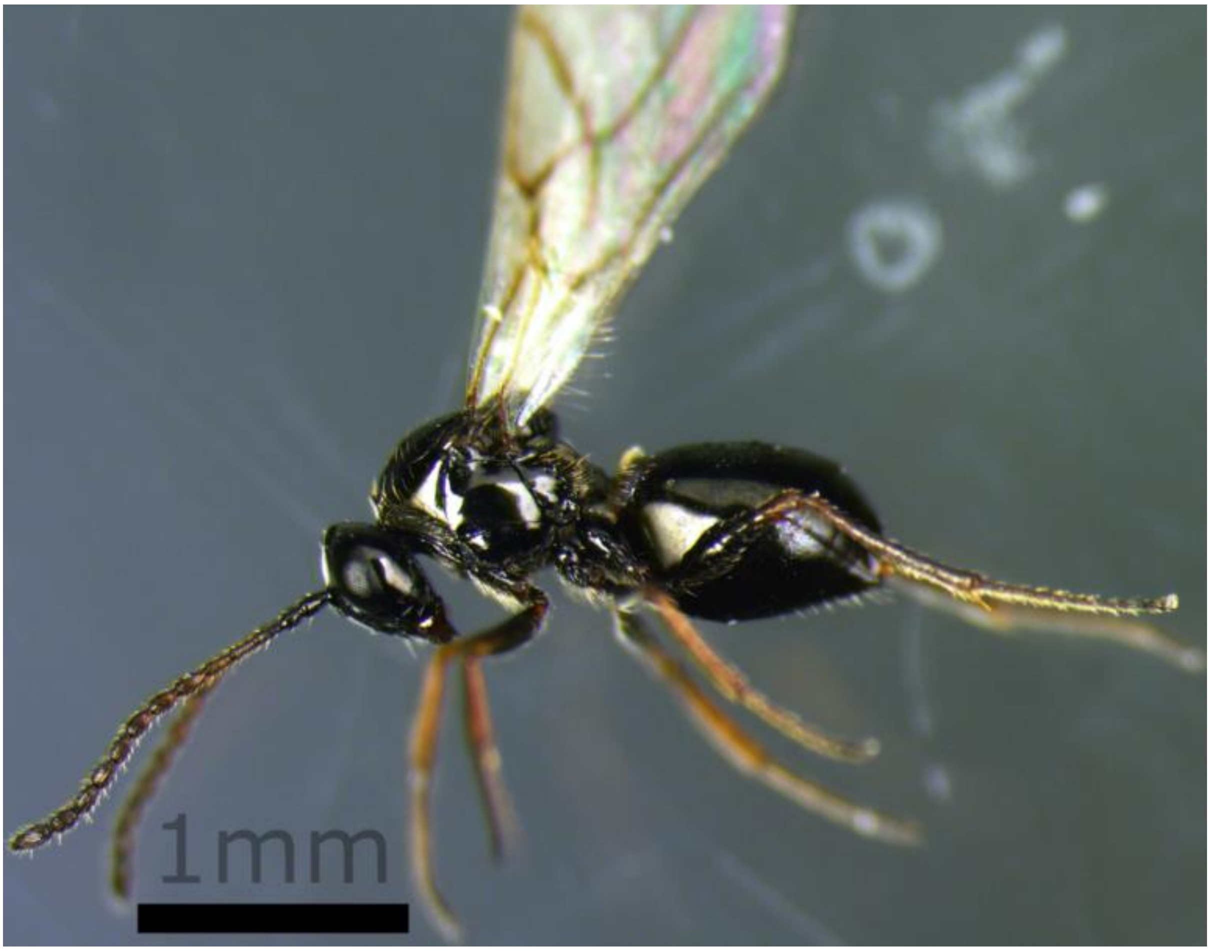

Hyposoter frigidus

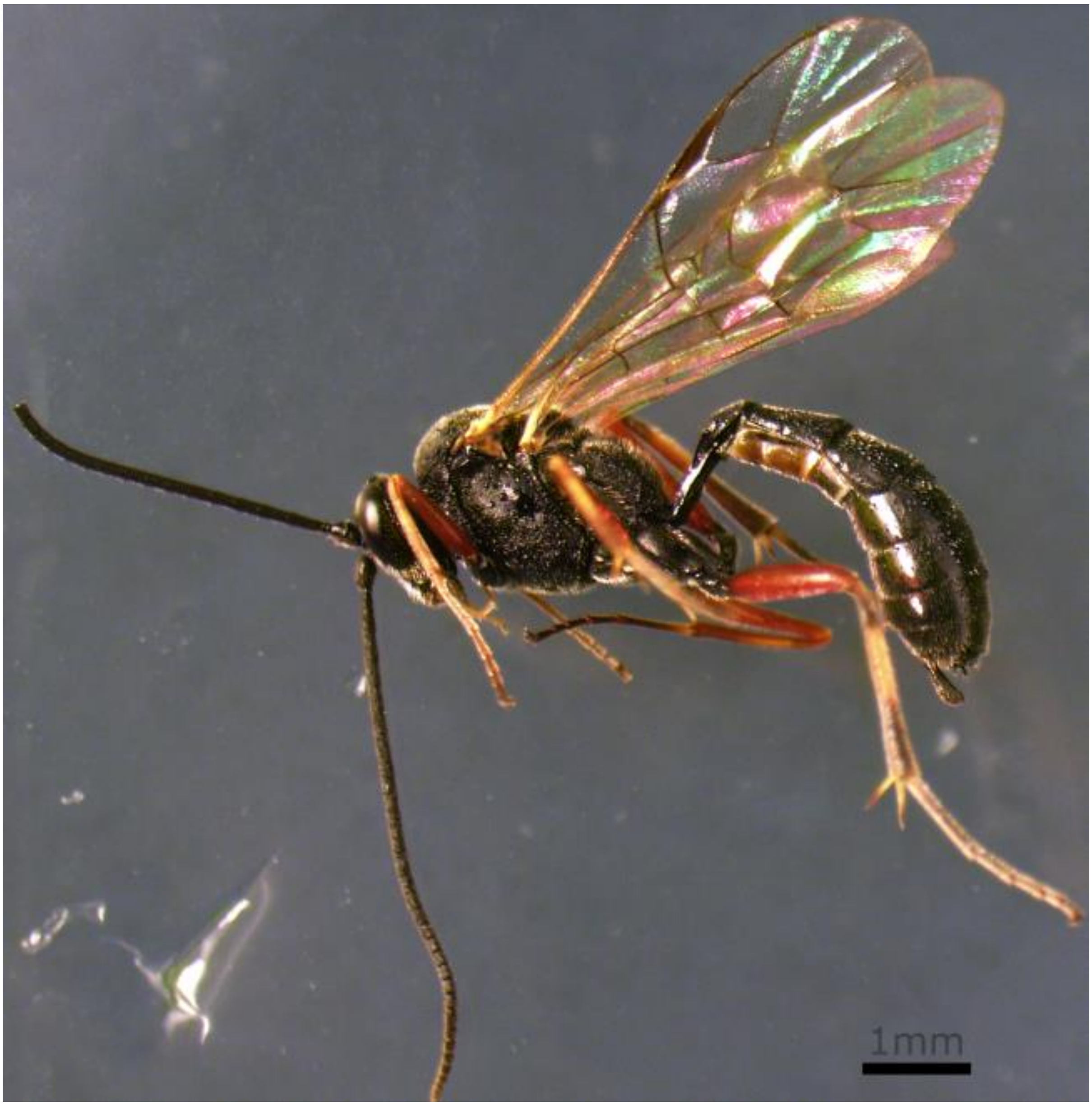

Hyposoter sp.

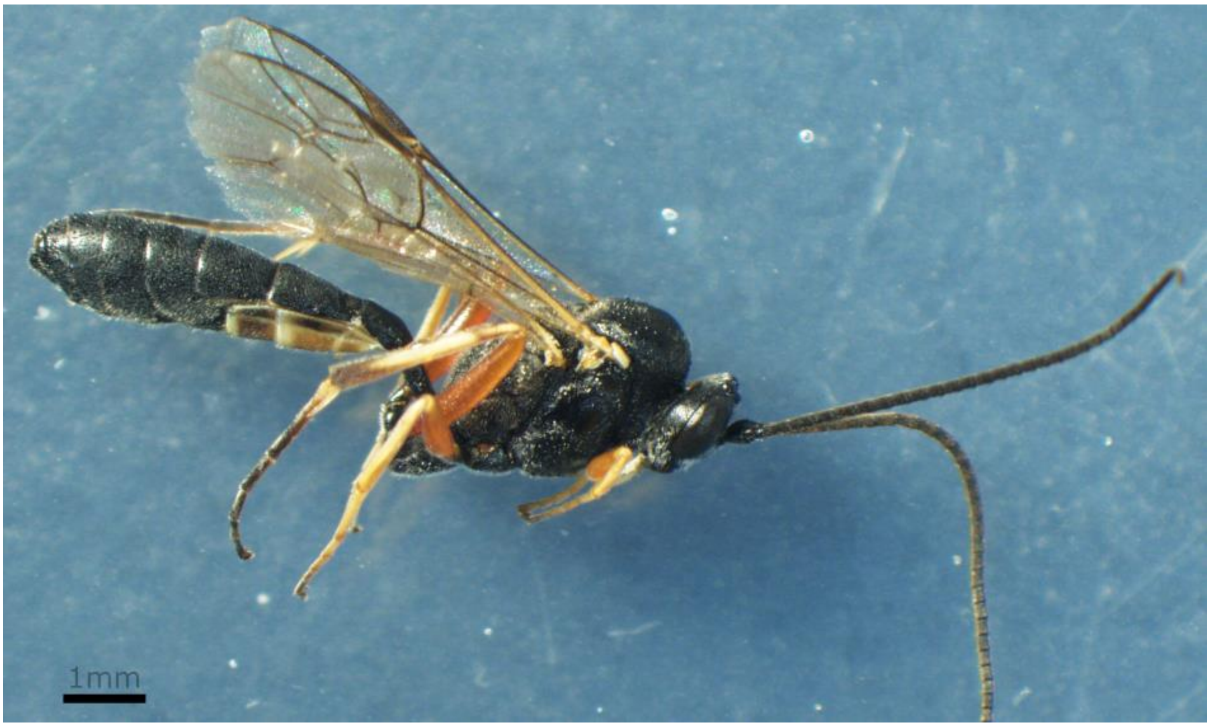

Ichneumon sarcitorius

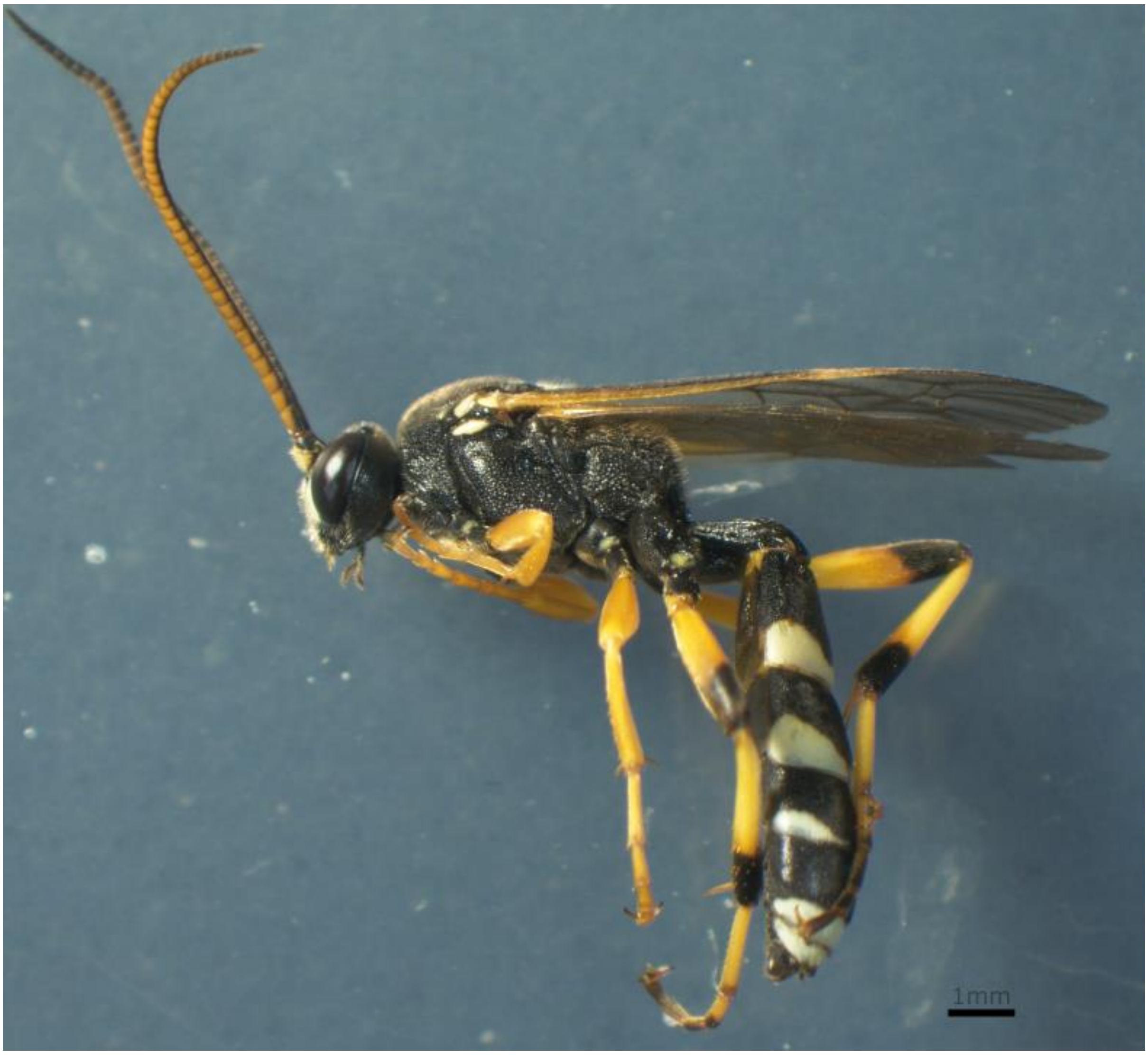

Ichneumonidae sp.

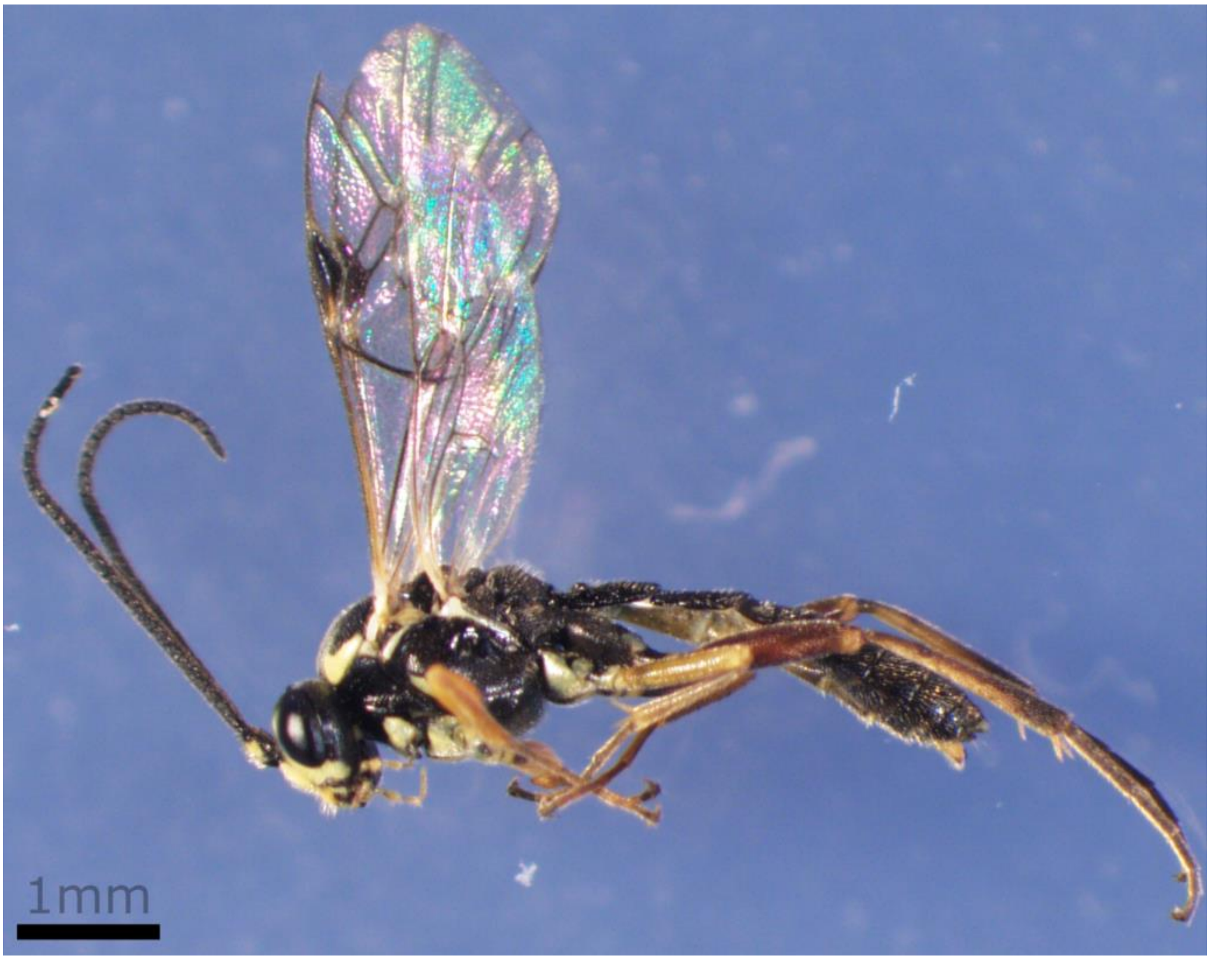

Meteorus rubens

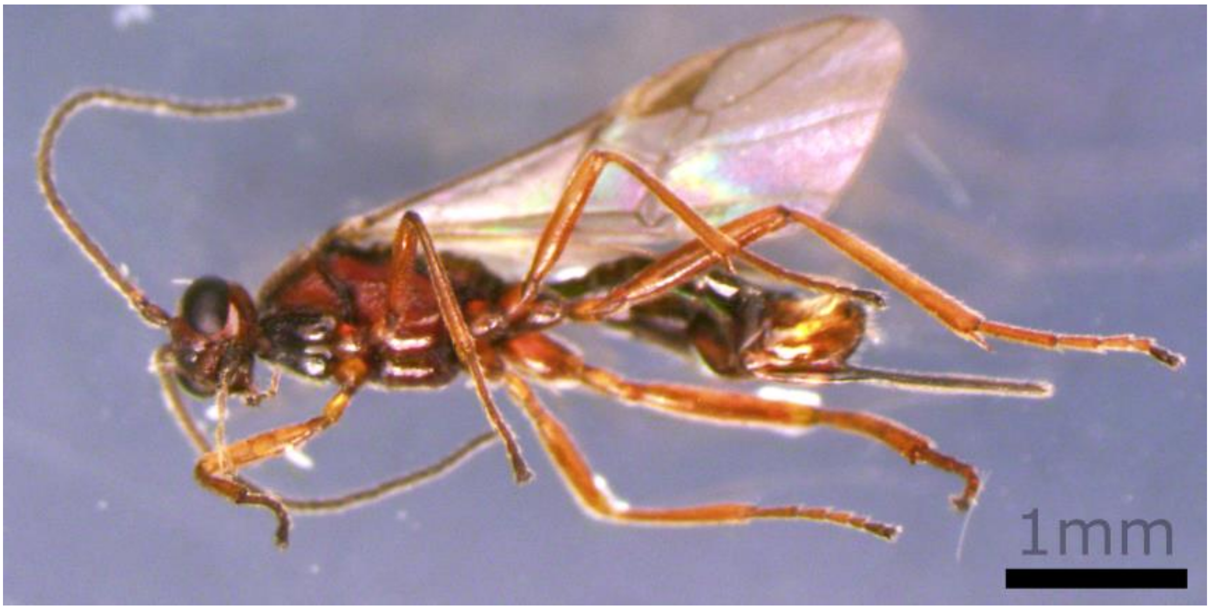

Panthisarthrus lubricus

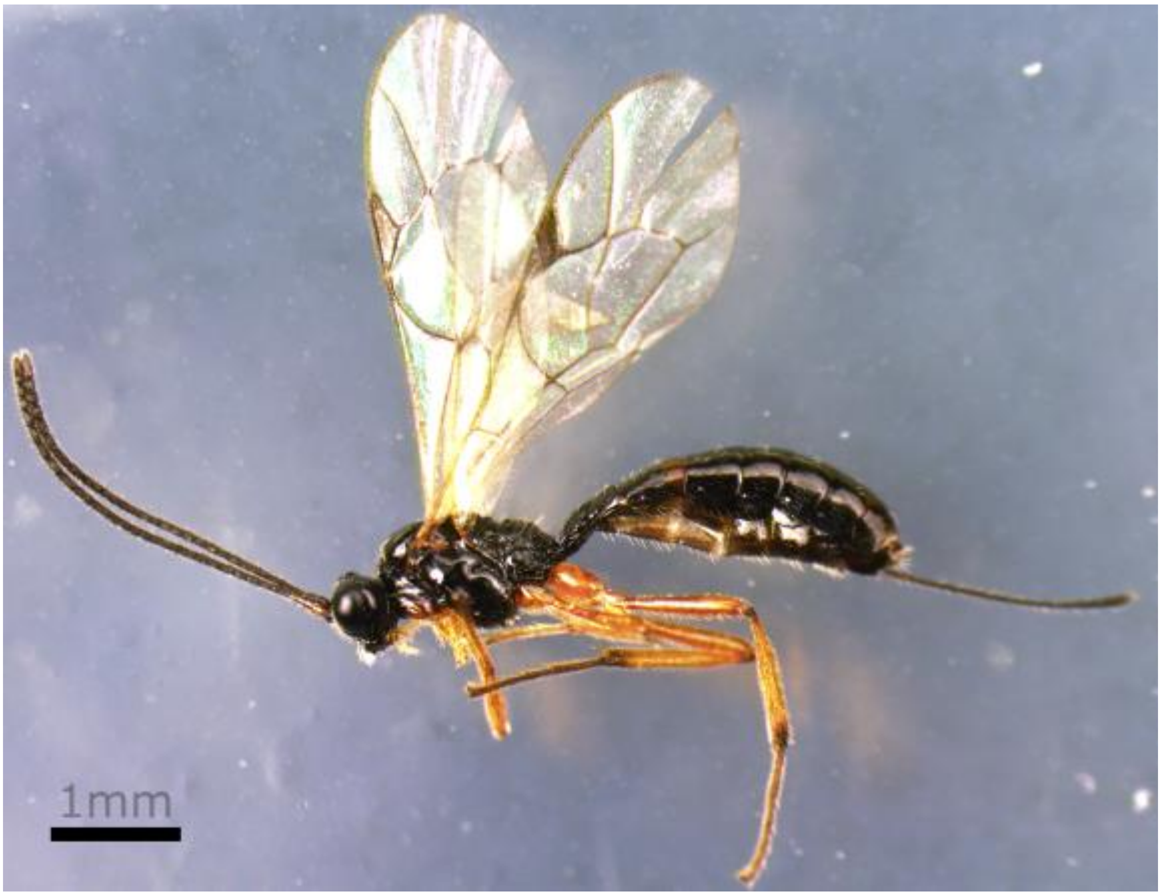

Protopanatales fulvipes

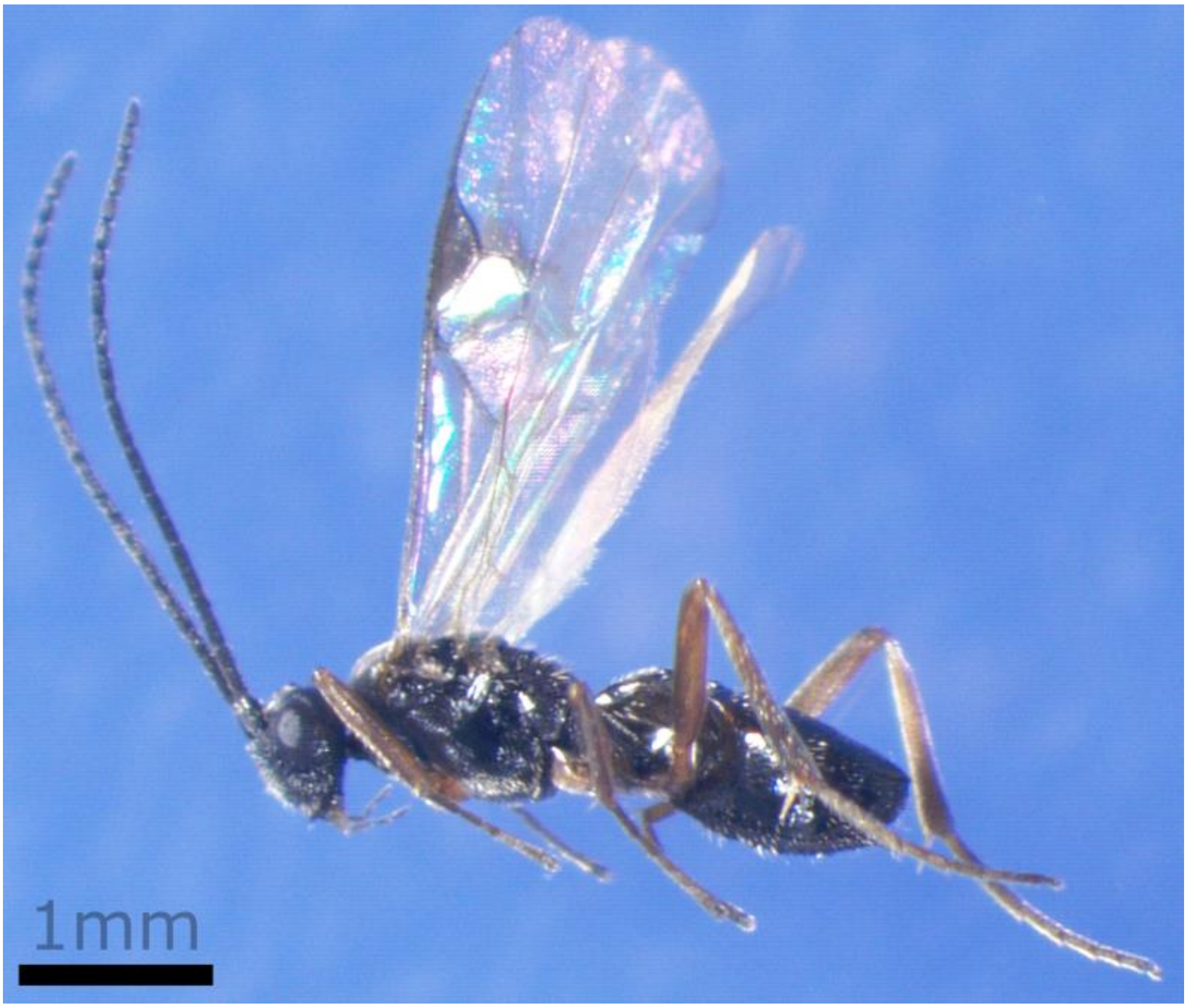

Tenthredinidae sp.

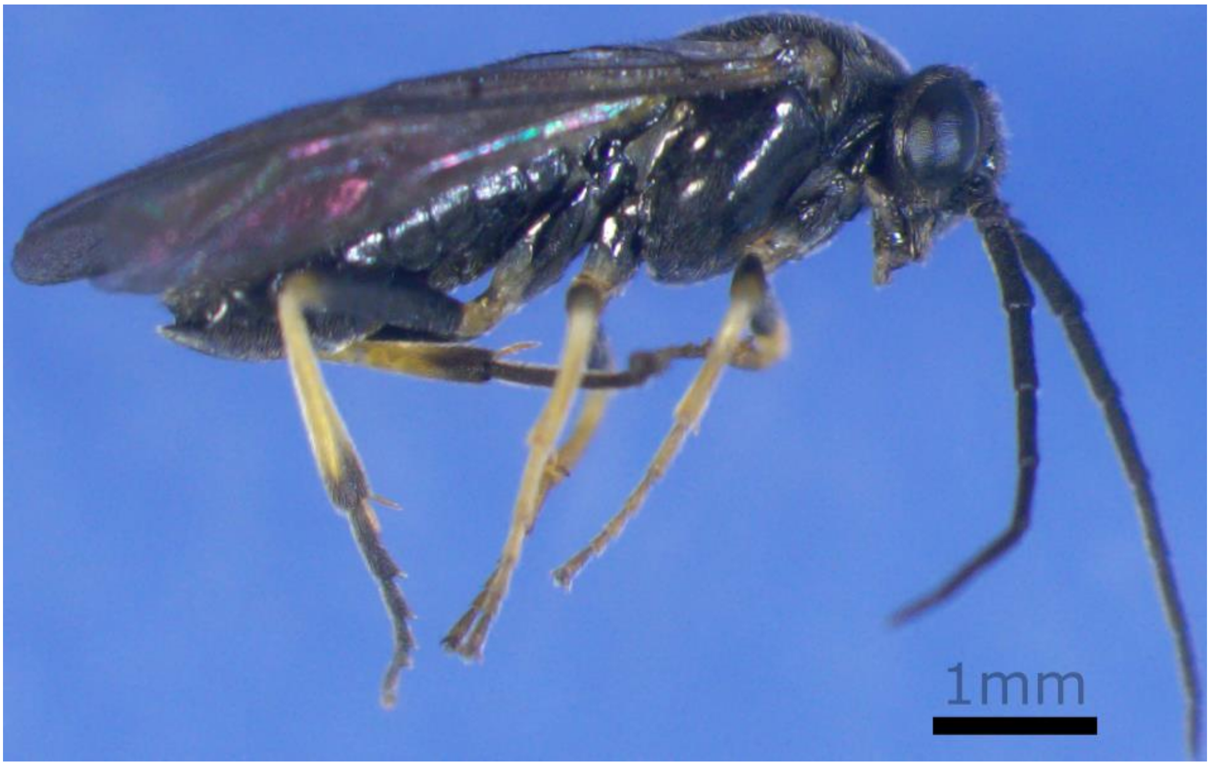

Lepidoptera

Acleris caryosphena

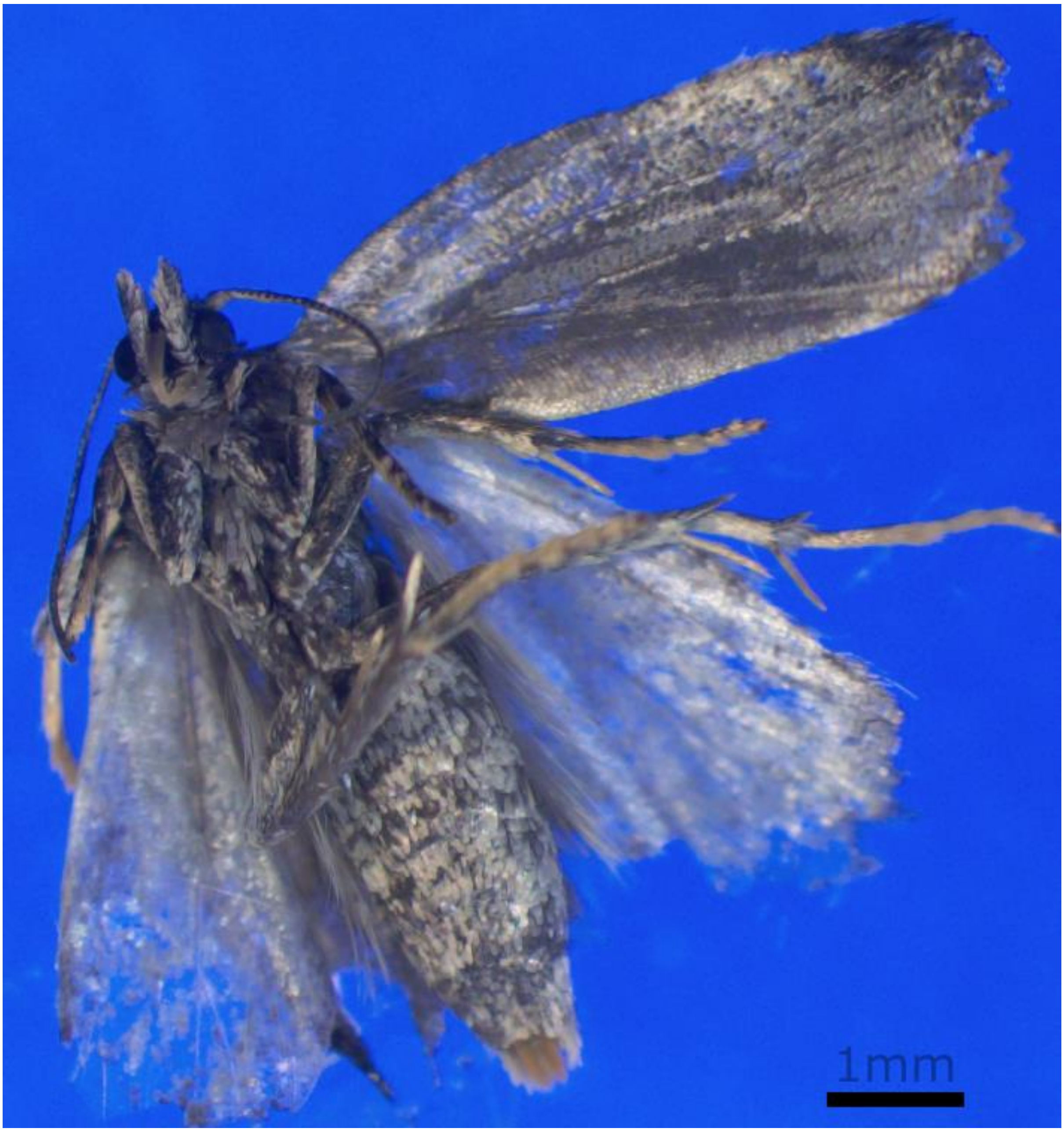

Autographa gamma

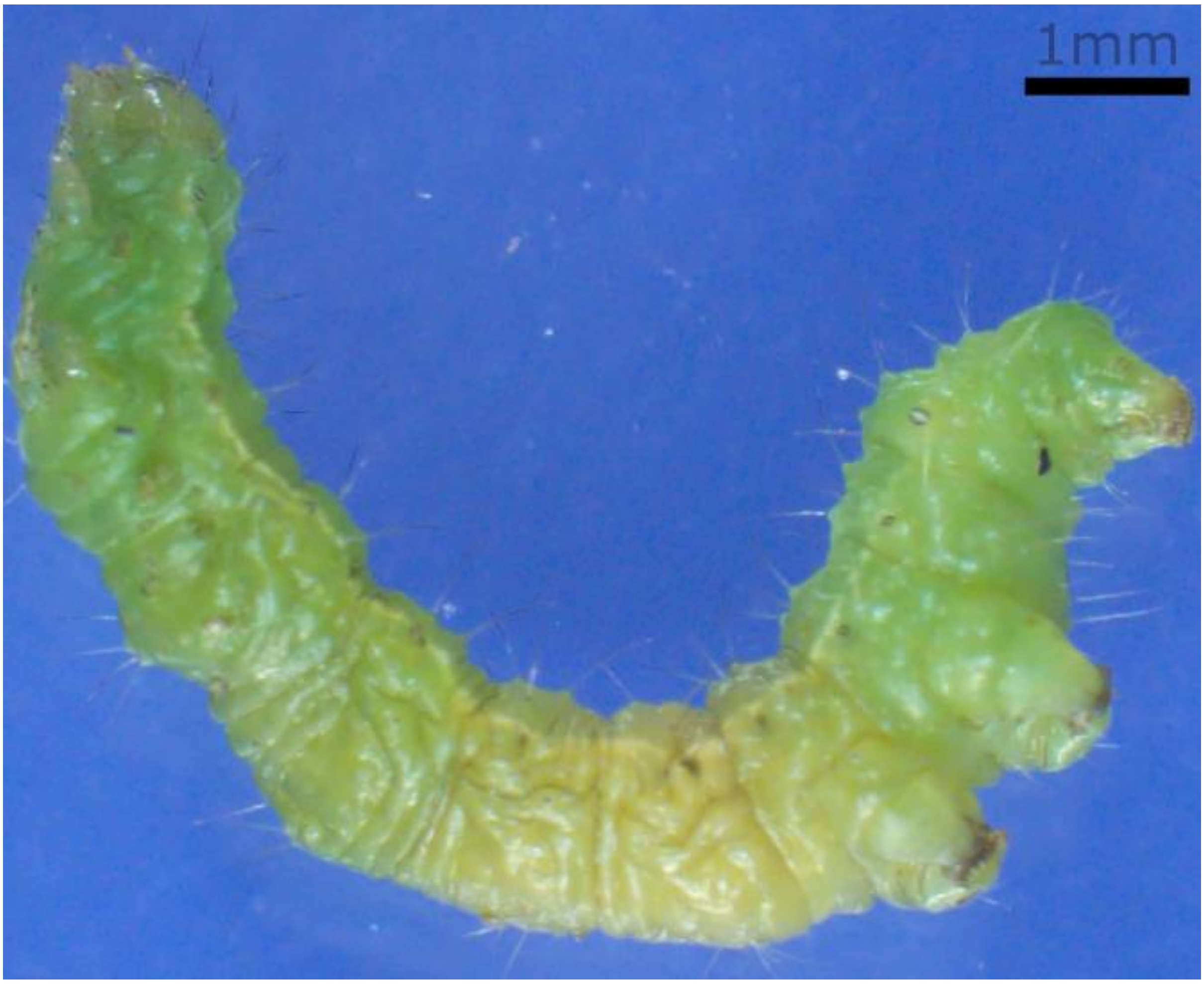

Eurois occulta

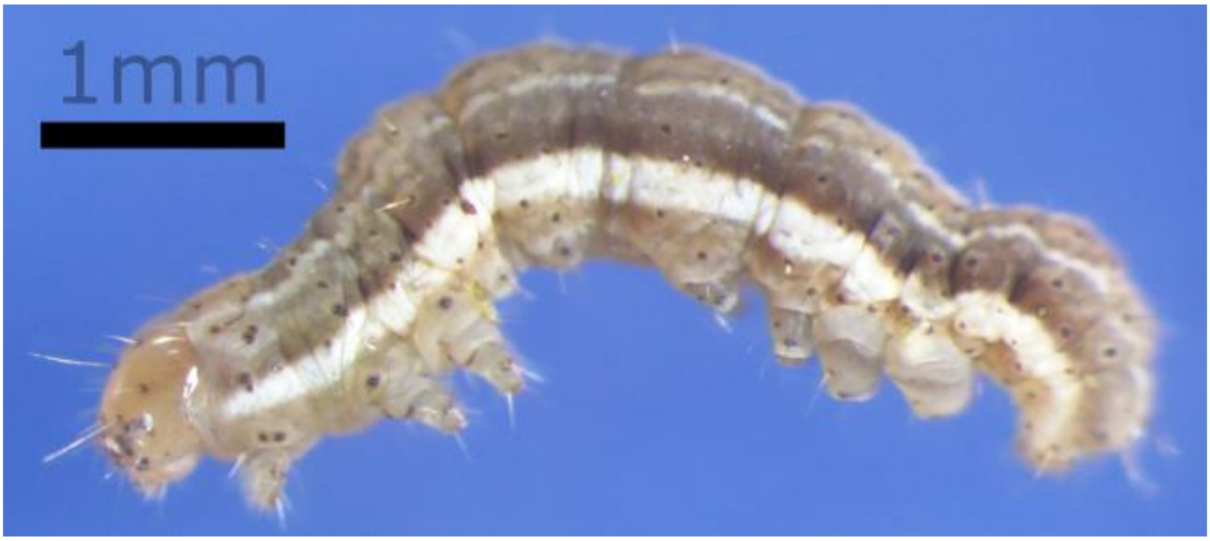

Mniotype adusta

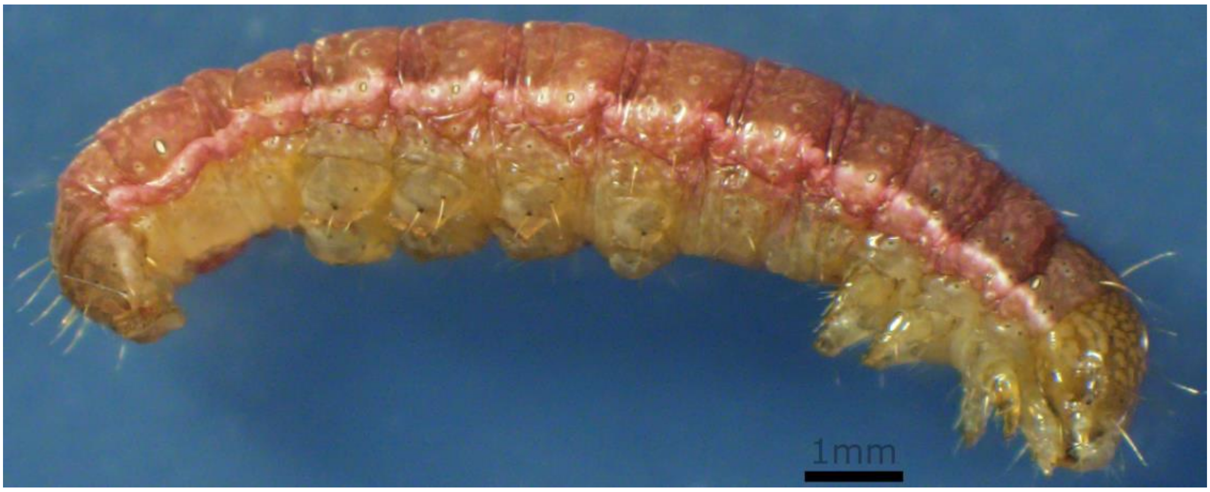

Scythris noricella

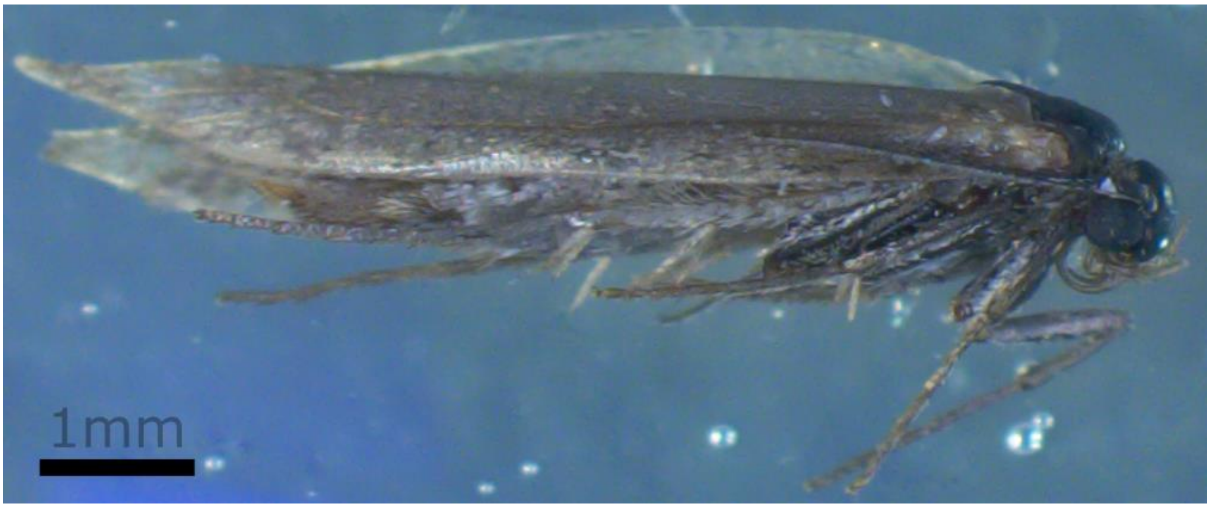

Spaelotis clandestine

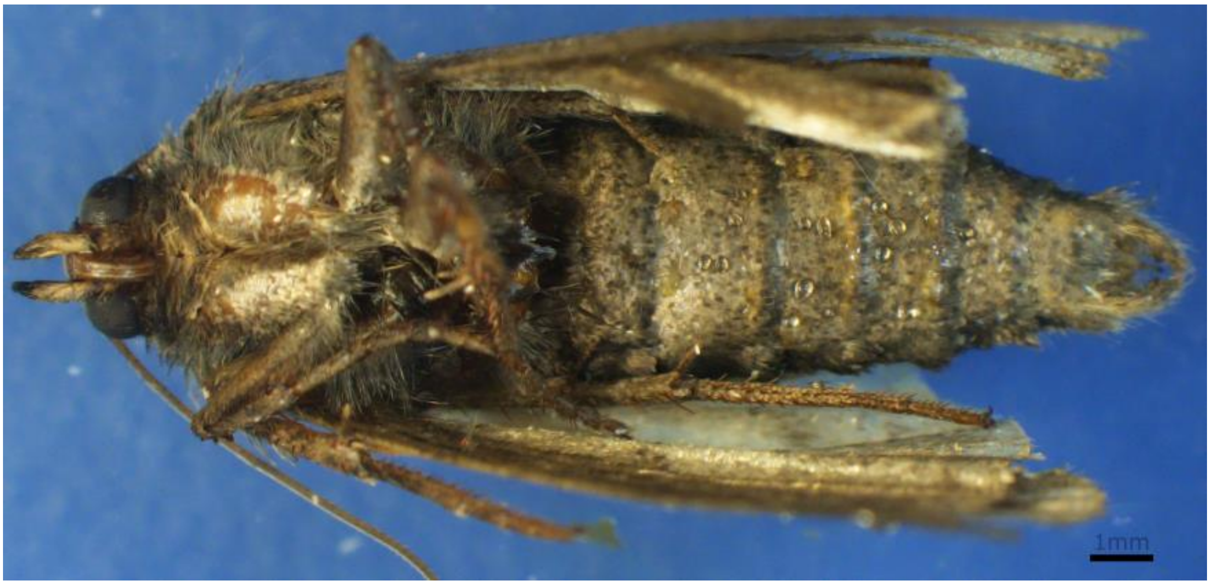

Syngrapha borea

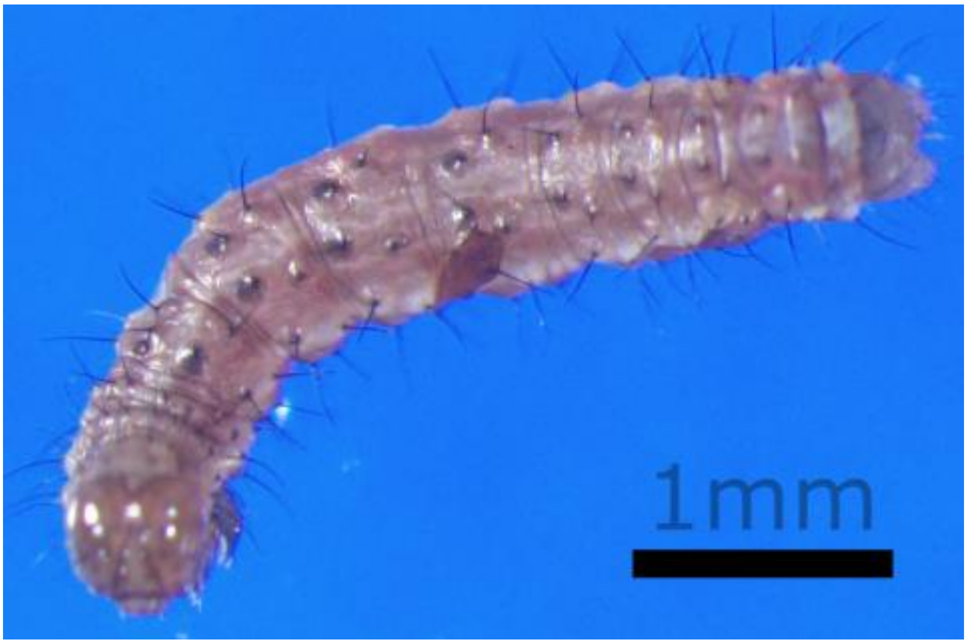

Torticidae sp.

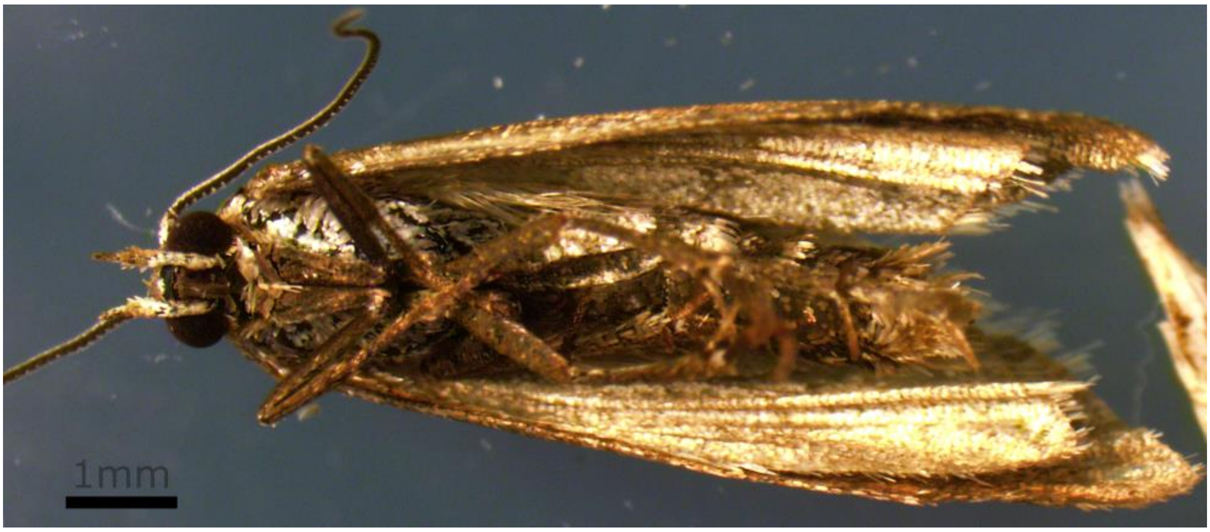

Neuroptera

Hemerobius ovalis

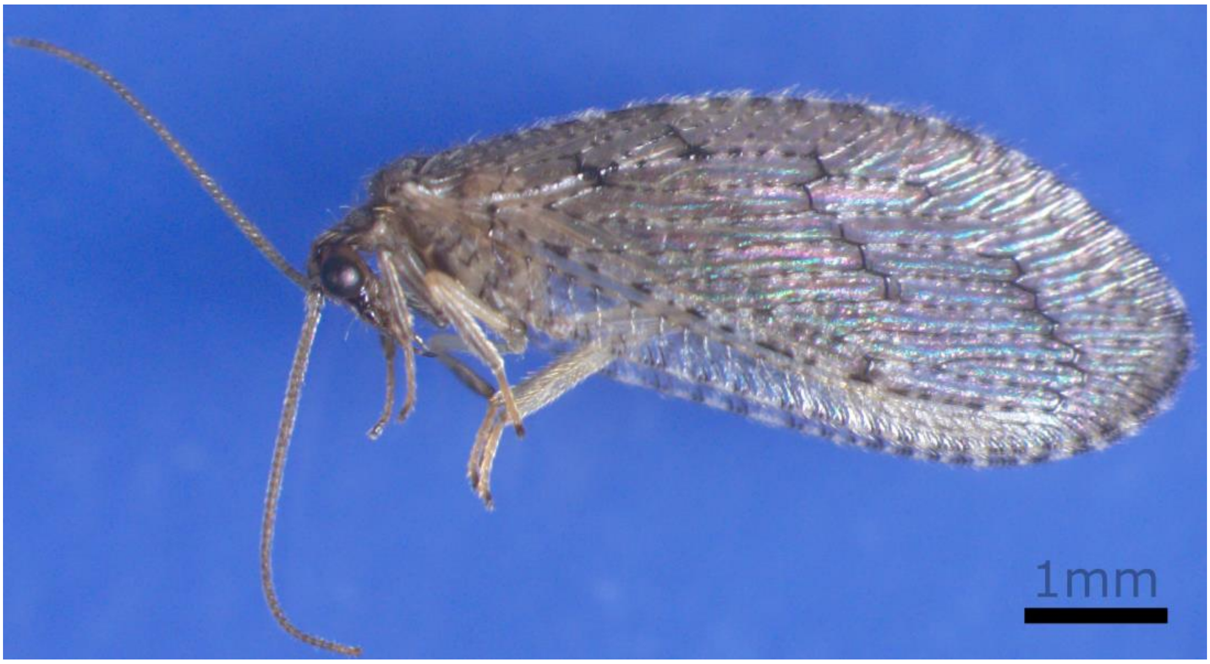

Wesmaelius nervosus

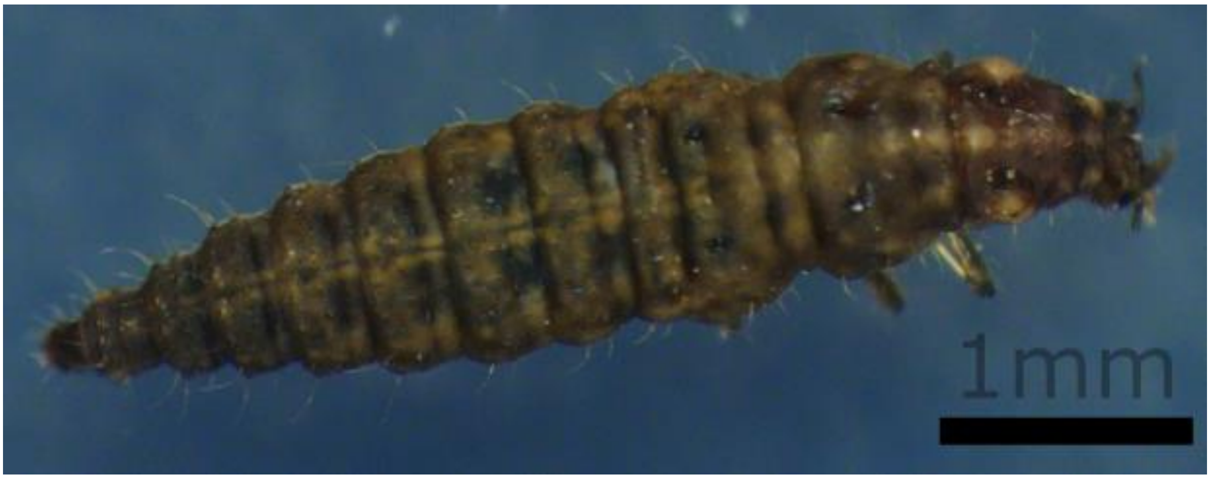

Trichoptera

Limnephilus kennicotti

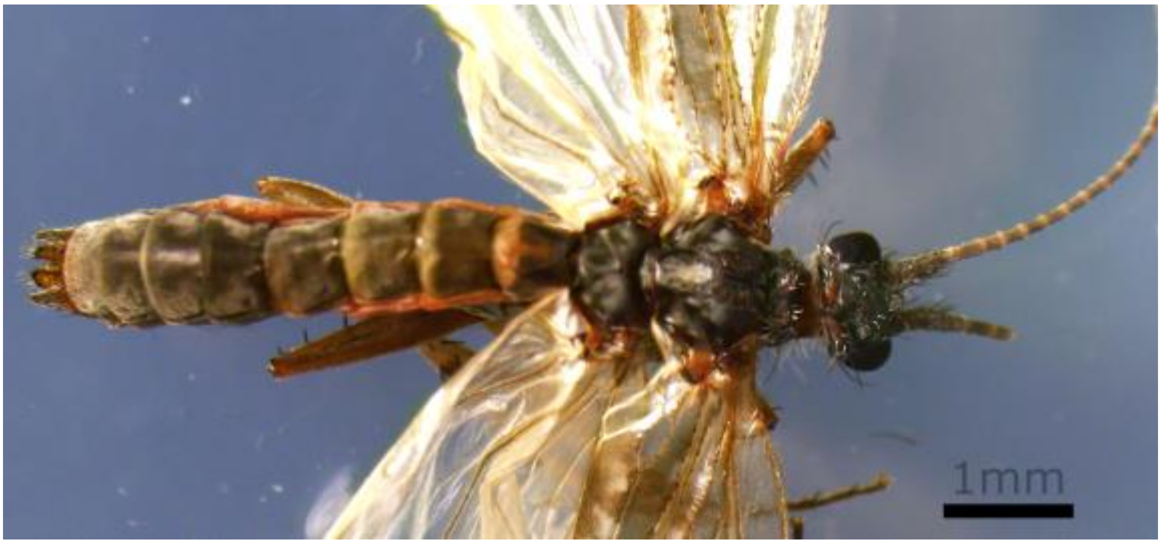

Limnephilus sp.

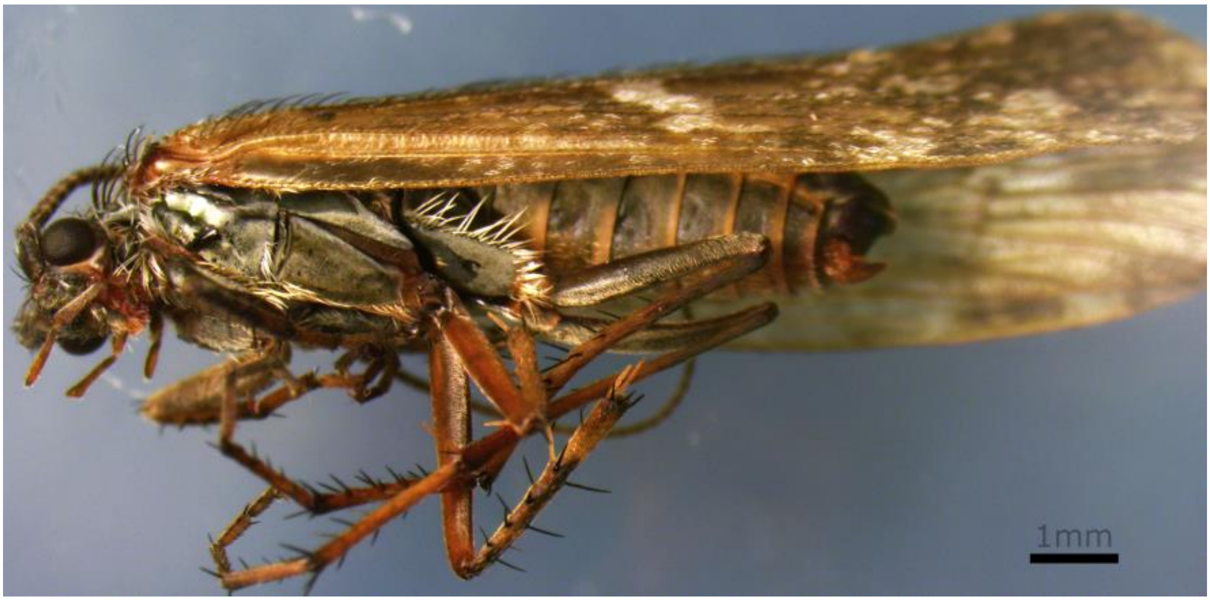

Trombidiformes

Anystidae sp.

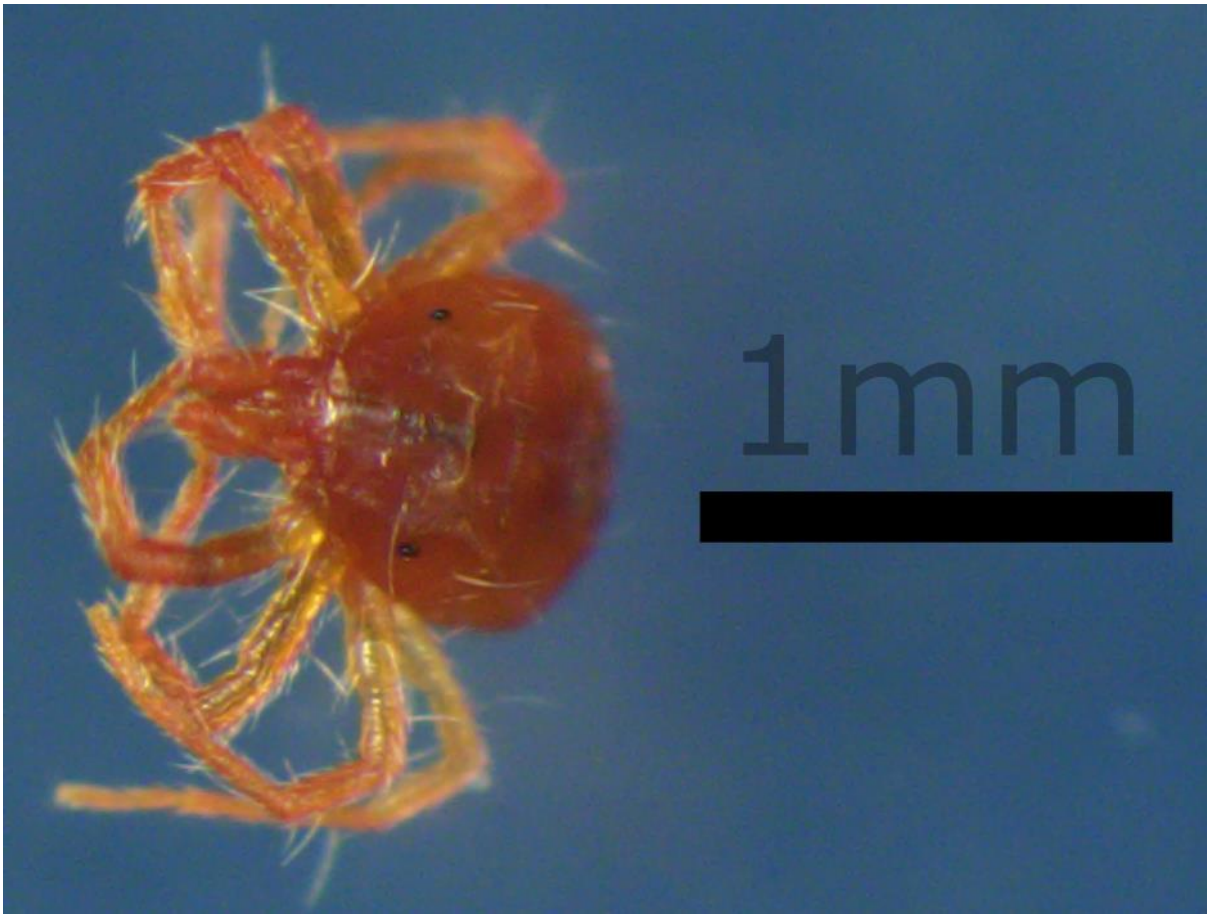

Supplement: Supplementary file 2 — Appendix S2: Registry of identified taxons and representative images. [file GCB-32-e70687-s003.pdf]
