# Supplementary material for: Heat but Not Cold Tolerance Is Phylogenetically Constrained in Greenlandic Terrestrial Arthropods Under Future Global Warming
Source: Glob Chang Biol. 2026 Jan 8;32(1):e70687. doi: 10.1111/gcb.70687 (PMC12783425; doi:10.1111/gcb.70687)
Supplement: Supplementary file 4 — Appendix S4: gcb70687‐sup‐0004‐AppendixS4.docx. [file GCB-32-e70687-s001.docx]

**Supplemental Figures:**

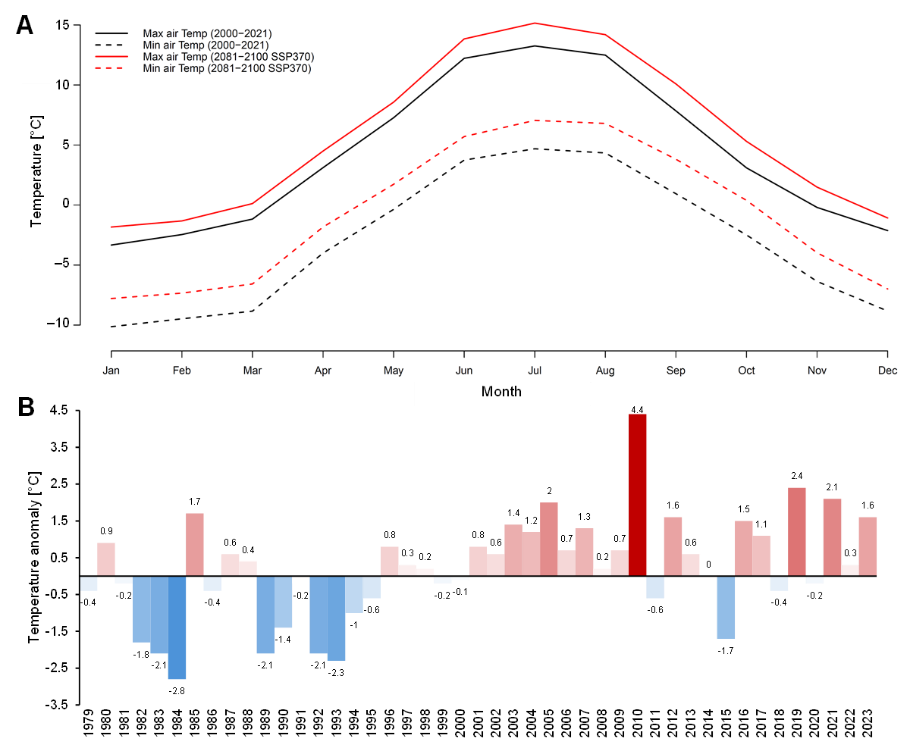


***Supplemental Figure S1.* A.** Air temperatures in Narsarsuaq, Greenland (61.160N, 45.424W) showing monthly maximum and minimum temperatures (solid and dashed lines, respectively) in recent years (2000-2021 black lines) and projected at end-of-century conditions (2081-2100 red lines) under the Shared Socio-economic Pathway SSP370 scenario. Historic temperatures are obtained from CRU-TS 4.06 ^1^ , and downscaled with WorldClim 2.1 ^2^ to a 10 arcmin resolution, while future projections are averaged across four CMIP6 Global Circulation Models: ACCESS-CM2, CMCC-ESM2, MIROC6, and MPI-ESM1-2-HR, see references ^3–6^ . **B.** Mean yearly air temperature anomalies, showing yearly deviations from the 1979–2023 baseline with colours ranging from colder than average (blue) to warmer than average (red). Data are from the ECMWF Reanalysis v5 (ERA5) produced by the Copernicus Climate Change Service ^7^

***Supplemental Table S1:*** *Microclimatic temperature profiles for Narsarsuaq sampling area. Method indicates the method of data collection,* ***Air Temp:*** *Meterological Air Temperature, measured at 2 m* ^1^*.* ***Tomst Logger:*** *TMS4-loggers (TOMST) located in sampling area.* ***Tomst Logger Mean:*** *Average of*

*Tomst Logger A and Tomst Logger B, averaged per hour.* ***Easylog data:*** *Temperature data from Noer et. al. 2022*^11^*, recorded using Easylog EL-USB-1 loggers (Lascar Electronics) in sampling area.* ***In silico:*** *Simulated temperature data based on either climatic conditions (NicheMapR) or collected microclimatic measurements (SSP370 projection). Height indicates the height at which data is collected or the height at which data used in models were collected. Max and Min Temp. indicate the highest and lowest temperature measured using the indicated method, while Mean temp. Is the average temperature* **±** *the standard deviation of the measurements for the indicated method.*

| **Year** | **Method** | **Height** | **Mean temp. ± SD (˚C)** | **Min. Temp. (˚C)** | **Max. Temp. (˚C)** | **Reference** |
| --- | --- | --- | --- | --- | --- | --- |
| 2023 | Air Temp | 200 cm | 8.64 ± 4.01 | -2.82 | 14.63 | Unpublished |
| 2023 | Tomst Logger A (Shade) | 15 cm | 13.78 ± 7.46 | 1 | 33.34 | Unpublished |
| 2023 | Tomst Logger A (Shade) | 2 cm | 14.28 ± 7.19 | 2 | 33.13 | Unpublished |
| 2023 | Tomst Logger A (Shade) | -6 cm | 15.83 ± 3.07 | 10.19 | 37.44 | Unpublished |
| 2023 | Tomst Logger B (Shade) | 15 cm | 12.71 ± 9.48 | -3 | 33.75 | Unpublished |
| 2023 | Tomst Logger B (Shade) | 2 cm | 13.16 ± 10.58 | -3.75 | 37.75 | Unpublished |
| 2023 | Tomst Logger B (Shade) | -6 cm | 12.9 ± 3.92 | 3.38 | 29.38 | Unpublished |
| 2023 | Tomst logger - Mean (Shade) | 15 cm | 13.24 | -0.47 | 32.43 | Unpublished |
| 2018 | Easylog data A (Sun) | 15 cm | 12.03 ± 8.1 | -2 | 41 | Noer et. al. 2022^11^ |
| 2018 | Easylog data B (Sun) | 5 cm | 12.01 ± 8.29 | -2.5 | 41 | Noer et. al. 2022^11^ |
| 2018 | Easylog data C (Shade) | 15 cm | 10.85 ± 5.94 | -1 | 33 | Noer et. al. 2022^11^ |
| 2018 | Easylog data D (Shade) | 5 cm | 10.87 ± 6.65 | -1 | 41.5 | Noer et. al. 2022^11^ |
| NicheMapper | In silico | na | 11.07 ± 6.39 | -4.53 | 27.53 | Kearny et. al. 2017^10^ |
| SSP370 - Projection | In silico | 15 cm | 18.44 ± 9.34 | 3.5 | 39.16 | Unpublished |


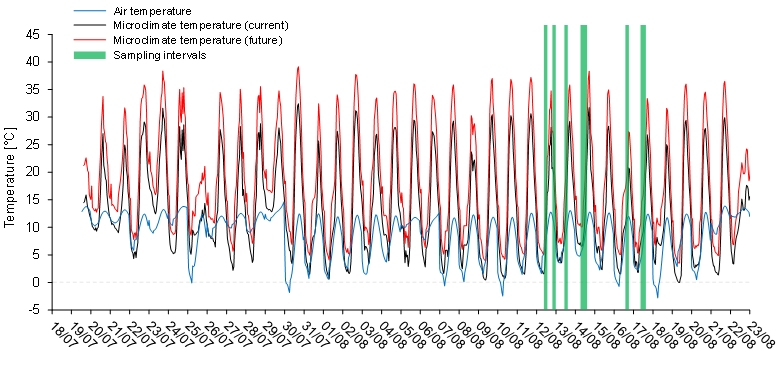
***Supplemental Figure S2.*** Air temperatures (blue line) and microclimate temperatures under current conditions (black line: measured with TOMST loggers 5 min interval, here averaged per hour) and projected future conditions (red line) in the period 19 July to 23 August 2023 prior and post sampling of insects in the present study (sampling intervals are highlighted in green vertical bars). Using weather data from the National Centers for Environmental Prediction ^8^ ^,^ hourly air temperatures were estimated at the sampling location 2 m above the surface with the ‘NicheMapR’ package v3.2.1 considering solar radiation, topology, soil properties, air and surface temperatures, and windspeed and -direction^9,10^ . To obtain hourly future microclimate temperatures (T_micro_future_), first we calculated the difference in air temperatures between recent years (average monthly maximum and minimum temperatures 2000-2021) and projected at end-of-century conditions (2081-2100) under the SSP370 scenario (see Figure S1 for details). This difference, ΔT_air_, i.e. future T_max_-current T_max_ during daytime (8AM-8PM) and future T_min_-current T_min_ during night, was added to the measured current microclimate (T_micro_current_) based on the exponential relationship between air and microclimate temperatures for day and night (see main text and Figure S3).


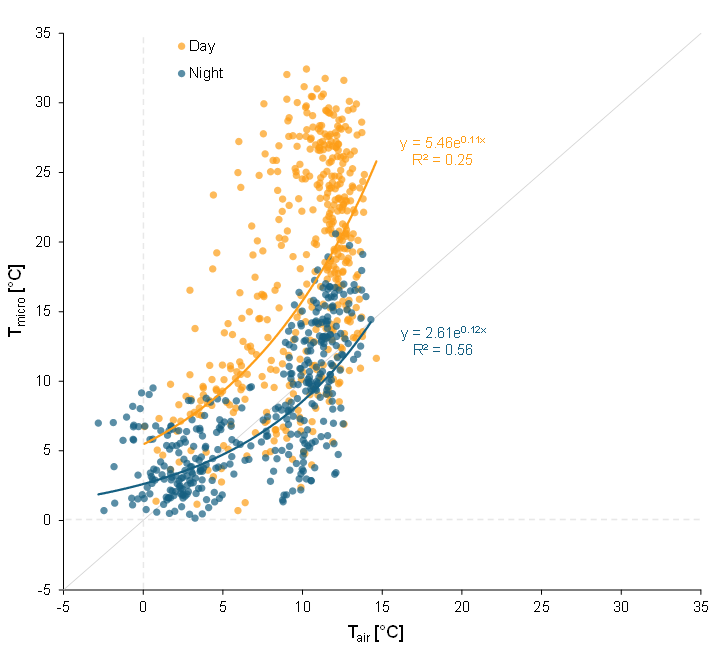
***Supplemental Figure S3.*** Relationship between hourly air temperature (T_air_) and measured microclimate temperatures (T_micro_; TOMST loggers) for day (8AM-8PM; orange dots and exponential fit), and night (8PM-8AM; blue dots and exponential fit). The solid grey line represents the line of unity (1:1).

***Supplemental Table S2:*** *Periods of time in which microclimate temperature exceeds recorded CT_max_ values for recorded microclimate data (2023) and end of the century projection (SSP370) within the 37 day-span (864 hours) between 18/07-2023 and 23/08-2023. Data is only presented for species which will exceed their CT_max_ at least one time in one of the scenarios. % indicate percentage of days/hours in the highlighted period.*

| **Species** | **2023**  **(days)** | **SSP370**  **(days)** | **2023**  **(hours)** | **SSP370**  **(hours)** |
| --- | --- | --- | --- | --- |
| *Delia platura* | 31 (89 %) | 33 (94 %) | 184 (22 %) | 298 (35 %) |
| *Calliphora uralensis* | 27 (77 %) | 32 (91 %) | 118 (14 %) | 241 (29 %) |
| *Eupeodes sp.* | 0 | 27 (77 %) | 0 | 119 (14 %) |
| *Spilogona arctica* | 0 | 25 (71 %) | 0 | 93 (11 %) |
| *Macrocera sp.* | 0 | 22 (63 %) | 0 | 82 (10 %) |
| *Protophormia terranovae* | 0 | 17 (49 %) | 0 | 58 (7 %) |
| *Mycetophila sp.* | 0 | 15 (43 %) | 0 | 49 (6 %) |
| *Forcipomyia sp.* | 0 | 15 (43 %) | 0 | 49 (6 %) |
| *Exechia sp.* | 0 | 13 (37 %) | 0 | 42 (5 %) |
| *Sciaridae sp.* | 0 | 5 (14 %) | 0 | 14 (2 %) |
| *Halocladius variabilis* | 0 | 5 (14 %) | 0 | 14 (2 %) |
| *Gyrinus opacus* | 0 | 4 (11 %) | 0 | 11 (1 %) |
| *Pegomya sp.* | 0 | 4 (11 %) | 0 | 11 (1 %) |
| *Scathophagidae sp.* | 0 | 3 (9 %) | 0 | 8 (1 %) |
| *Nebria rufescens* | 0 | 3 (9 %) | 0 | 8 (1 %) |
| *Hydrophorus morio* | 0 | 1 (3 %) | 0 | 4 (0 %) |
| *Muscidae sp.* | 0 | 1 (3 %) | 0 | 3 (0 %) |
| *Scathophaga sp.* | 0 | 1 (3 %) | 0 | 3 (0 %) |

**Supplemental references:**

1. Harris, I., Osborn, T. J., Jones, P. & Lister, D. Version 4 of the CRU TS monthly high-resolution gridded multivariate climate dataset. *Sci Data* **7**, 109 (2020).

2. Fick, S. E. & Hijmans, R. J. WorldClim 2: new 1‐km spatial resolution climate surfaces for global land areas. *International Journal of Climatology* **37**, 4302–4315 (2017).

3. von Storch, J.-S. *et al.* MPI-M MPIESM1.2-HR model output prepared for CMIP6 HighResMIP. Version 20241209.Earth System Grid Federation. <https://doi.org/10.22033/ESGF/CMIP6.762>. Preprint at (2017).

4. Shiogama, H., Abe, M. & Tatebe, H. MIROC MIROC6 model output prepared for CMIP6 ScenarioMIP. Version 20241209.Earth System Grid Federation. <https://doi.org/10.22033/ESGF/CMIP6.898>. (2019).

5. Dix, M. *et al.* CSIRO-ARCCSS ACCESS-CM2 model output prepared for CMIP6 CMIP historical. Version 20241209].Earth System Grid Federation. <https://doi.org/10.22033/ESGF/CMIP6.4271>. (2019).

6. Peano, D., Lovato, T. & Stefano, M. CMCC CMCC-ESM2 model output prepared for CMIP6 LS3MIP. Version 20241209.Earth System Grid Federation. <https://doi.org/10.22033/ESGF/CMIP6.13165>. Preprint at (2020).

7. Sabater, M. J. ERA5-Land hourly data from 1950 to present. Copernicus Climate Change Service (C3S) Climate Data Store (CDS). DOI: 10.24381/cds.e2161bac (Accessed on 09-12-2024). Preprint at (2019).

8. Kemp, M. U., Emiel van Loon, E., Shamoun‐Baranes, J. & Bouten, W. RNCEP: global weather and climate data at your fingertips. *Methods Ecol Evol* **3**, 65–70 (2012).

9. Kearney, M. R., Isaac, A. P. & Porter, W. P. microclim: Global estimates of hourly microclimate based on long-term monthly climate averages. *Sci Data* **1**, 140006 (2014).

10. Kearney, M. R. & Porter, W. P. NicheMapR – an R package for biophysical modelling: the microclimate model. *Ecography* **40**, 664–674 (2017).

11. Noer, N. K. *et al.* Rapid Adjustments in Thermal Tolerance and the Metabolome to Daily Environmental Changes – A Field Study on the Arctic Seed Bug Nysius groenlandicus. *Front Physiol* **13**, (2022).
